# Supplementary material for: Modular gateway-ness connectivity and structural core organization in maritime network science
Source: Nat Commun. 2020 Jun 5;11:2849. doi: 10.1038/s41467-020-16619-5 (PMC7275034; doi:10.1038/s41467-020-16619-5)
Supplement: Supplementary file 1 — Supplementary Information [file 41467_2020_16619_MOESM1_ESM.pdf]

Supplementary Information

Modular gateway-ness connectivity and structural core organization in maritime network science

Xu et al.

Supplementary Figures

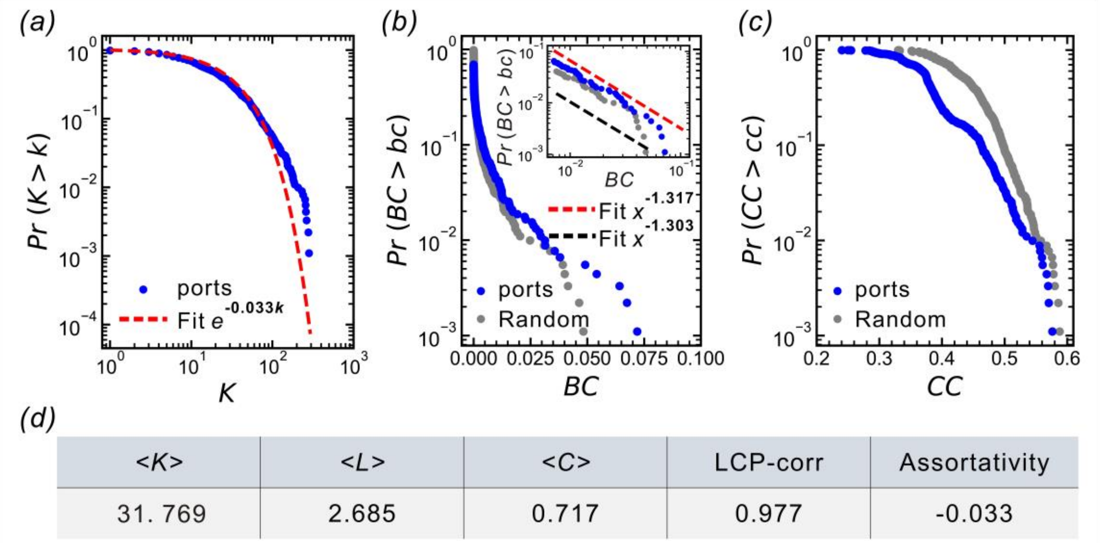

(d)

<K>	<L>	<C>	LCP-corr	Assortativity
31. 769	2.685	0.717	0.977	-0.033

Supplementary Fig. 1 Basic topological properties of the GLSN of 2017. In panel ***(a)***, complementary cumulative distribution function of degree is reported in log-log scale (dots) and fitted by an exponential function (dash line). Complementary cumulative probability distributions of betweenness centrality (*BC*) and closeness centrality (*CC*) for ports are plotted in semi-log scale in panel ***(b)*** and ***(c)***, respectively, in comparison to an equivalent random network which exactly keeps the same degree distribution as the original GLSN. The inset in panel ***(b)*** reports in log-log scale a power-law tail (red dash line) in the betweenness distribution with an exponent 1.317, corresponding to ports with *BC* ≥ 0.0071 (dots); power-law-ness is tested based on the method of Clauset et al[1]. For an equivalent random network, the betweenness distribution for nodes with *BC* ≥ 0.0071 decays with an exponent 1.303 (mean across 62.4% (624 out of 1,000) iterations passing the test) (black dash line). The average closeness centrality of the GLSN (i.e., 0.380) is close to that of an equivalent random graph (i.e., 0.438, mean value across 1,000 realizations). The bottom panel ***(d)*** presents the average port degree <*K*>, average shortest path length <*L*>, average clustering coefficient <*C*>, degree assortativity coefficient and local-community-paradigm correlation (LCP-corr). Source data are provided as a Source Data file.

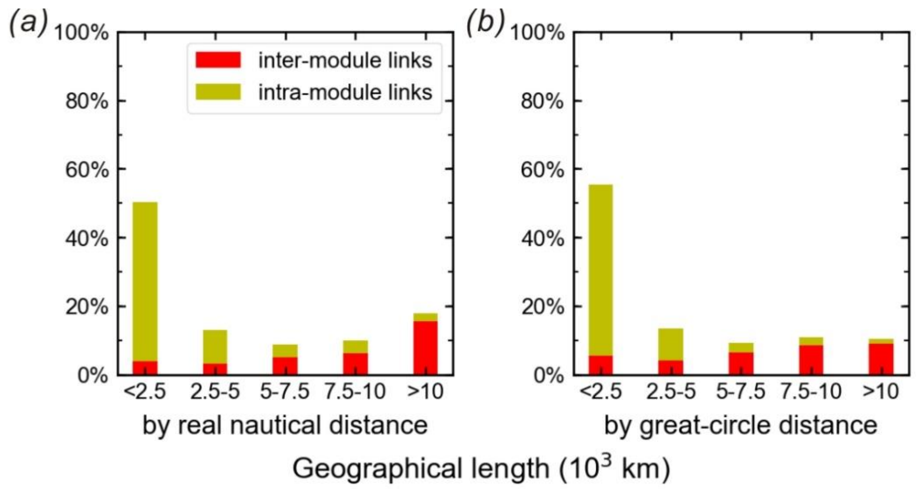

Supplementary Fig. 2 Proportional distribution of intra- and inter- module links in different range of geographical length. Specifically, we measured the geographical length of an inter-port link both as the real nautical distance and as the great-circle distance based on ports' geographical locations of latitude and longitude, finding that long-range links are relatively few and the majority of them are inter-module. The average length of inter-module links is 10,034 km (SD = 5,746 km) and that of intra-module links is 2,541 km (SD = 3,034 km), measured by real nautical distance; 7,635 km (SD = 4,405 km) and 2,090 km (SD = 2,683 km), measured by great-circle distance.

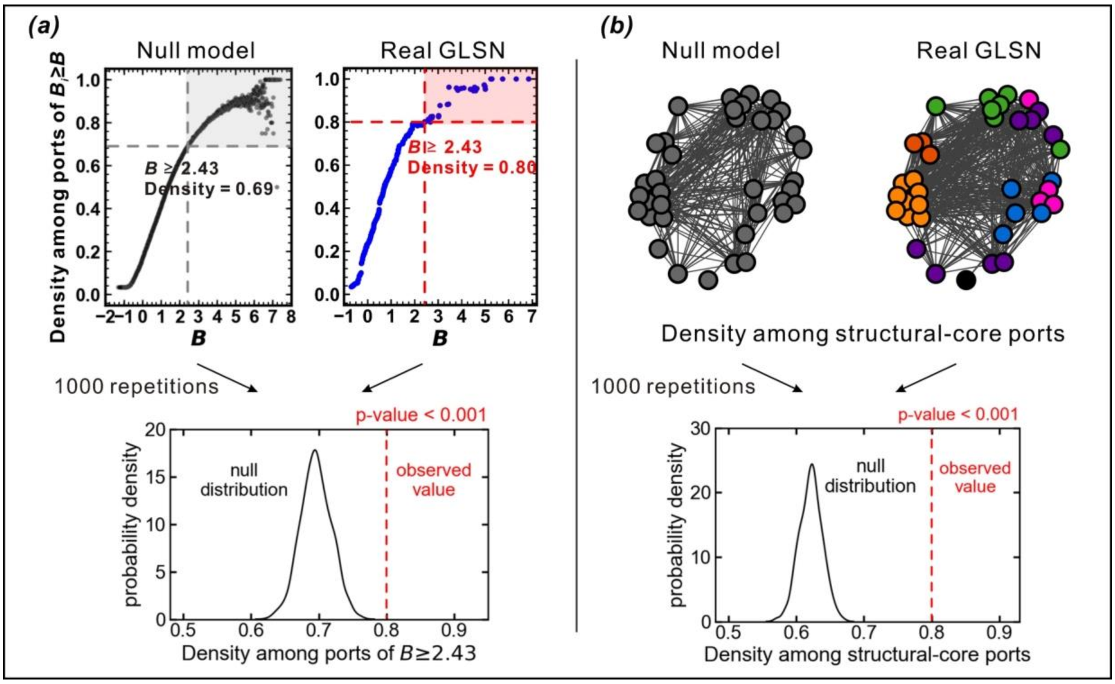

Supplementary Fig. 3 Statistical significance of the structural core in the real GLSN of 2015. A structural core that consists of 37 gateway-hub ports with $B \geq 2.43$ and forms a subgraph of density 0.80 is detected in the real GLSN, ***(a).*** Links among the structural-core ports in the real GLSN are represented in ***(b)***, with color indicating these ports' respective modules. We further evaluated the statistical significance of this structural core in the real GLSN, by accessing whether

the probability to detect the same structural core in a null configuration of the GLSN is significantly low. Specifically, two different statistical tests and respective *p*-values were computed: ***(a)***, the probability that in the null model the set of nodes with $B \geq 2.43$ form a subgraph of density larger than 0.80; ***(b)***, the probability that in the null model the same structural-core nodes of the real GLSN form a subgraph of density larger than 0.80. The result, obtained over 1,000 repetitions, is that the structural core of the real GLSN is very significant (*p*-value < 0.001), meaning that the probability to detect the same structural core at random is lower than 0.001. (See Supplementary Note 7 for pseudocode of the algorithm.)

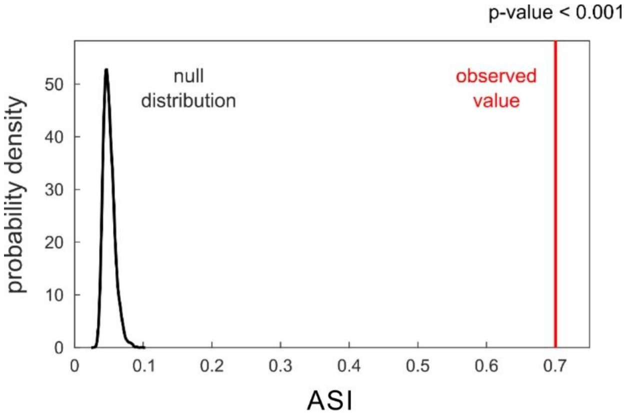

Supplementary Fig. 4 Statistical significance of the angular separation index. To compute a *p*-value that determines the significance of the angular separation index (ASI)[2] shown in Fig. 7 (in the main text), we created an ASI null distribution (reported in black) obtained by 10,000 random permutations of the nodes along the angular coordinate of the hyperbolic layout, and then computed the probability to generate at random a layout with a separation greater than or equal to 0.7 (the observed one, reported in red). The observed value of ASI is statistically significant, with *p*-value < 0.001. The plotted ASI null distribution is a probability density estimation performed using the MATLAB function ksdensity.

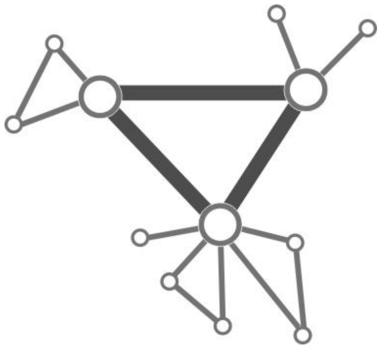

Supplementary Fig. 5 A classical hub-and-spoke service network configuration in liner shipping. The three hub ports, denoted by big dots, have very strong links (i.e., large traffic volumes) with each other. By contrast, the small feeder ports, denoted by small dots, have weak links (i.e., low traffic volumes) with those hub ports and also a few weak links between themselves. Such a

configuration allows shipping carriers to consolidate cargo in regional hub ports and transport the cargo between different regions by using larger vessels, thus lowering the transportation cost due to the economies of scale. This Figure is modified from the artwork of a previous study (Hu and Zhu, 2009)[3].

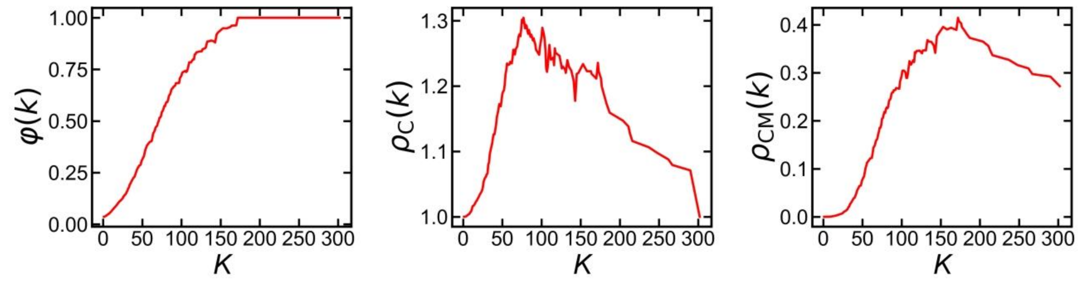

Supplementary Fig. 6 Rich-club coefficients of world ports. $\varphi(k)$ is the unnormalized version of the rich-club coefficient originally proposed in reference[4]; $\rho_{\mathrm{C}}(k)$ and $\rho_{\mathrm{CM}}(k)$, normalized versions proposed in reference[5] and in reference[6], respectively. $\varphi(k)$ is calculated as the ratio of the number of edges among the nodes with a degree larger than k to the maximum possible number of edges among these nodes. A popular normalization method of $\varphi(k)$, introduced by Colizza et al (2006), is expressed as $\rho_{\mathrm{C}}(k) = \varphi(k)/\varphi_{\mathrm{MS}}(k)$. $\varphi_{\mathrm{MS}}(k)$ is the rich-club coefficient of an equivalent random network obtained based on the randomization procedures developed by Maslov and Sneppen (2002)[7]; at any time step, it randomly selects a couple of links of the original network and exchanges two of their ending points (automatically preserving the degree distribution of the network under study). Another recent normalization method of $\varphi(k)$ was introduced by Muscoloni and Cannistraci (2017), expressed as $\rho_{\mathrm{CM}}(k) = \varphi(k) - \varphi_{\mathrm{CM}}(k)$. $\varphi_{\mathrm{CM}}(k)$ is the rich-club coefficient of an equivalent random network obtained based on the randomization procedures developed by Muscoloni and Cannistraci (2017)[6]. Their procedures are actually a generation of the Maslov and Sneppen's procedures, but at each time step the two probabilities of the two sampled swapping edges are defined separately in function of the adjacent nodes' degree and have an inverted tendency. For instance, one edge is sampled with probability directly proportional to the product of the degrees of the adjacent nodes, whereas the other edge with probability inversely proportional. We notice that the ρ_{CM} has one (and only one) peak at the degree of 172; it was here preferred to the other rich-club-ness measures because it offers a smoother trend and clear peak (at higher degree than other measures) with which the detection of a compact rich club with high degree threshold is patent. Both the φ and the ρ_{C} do not display a neat peak value.

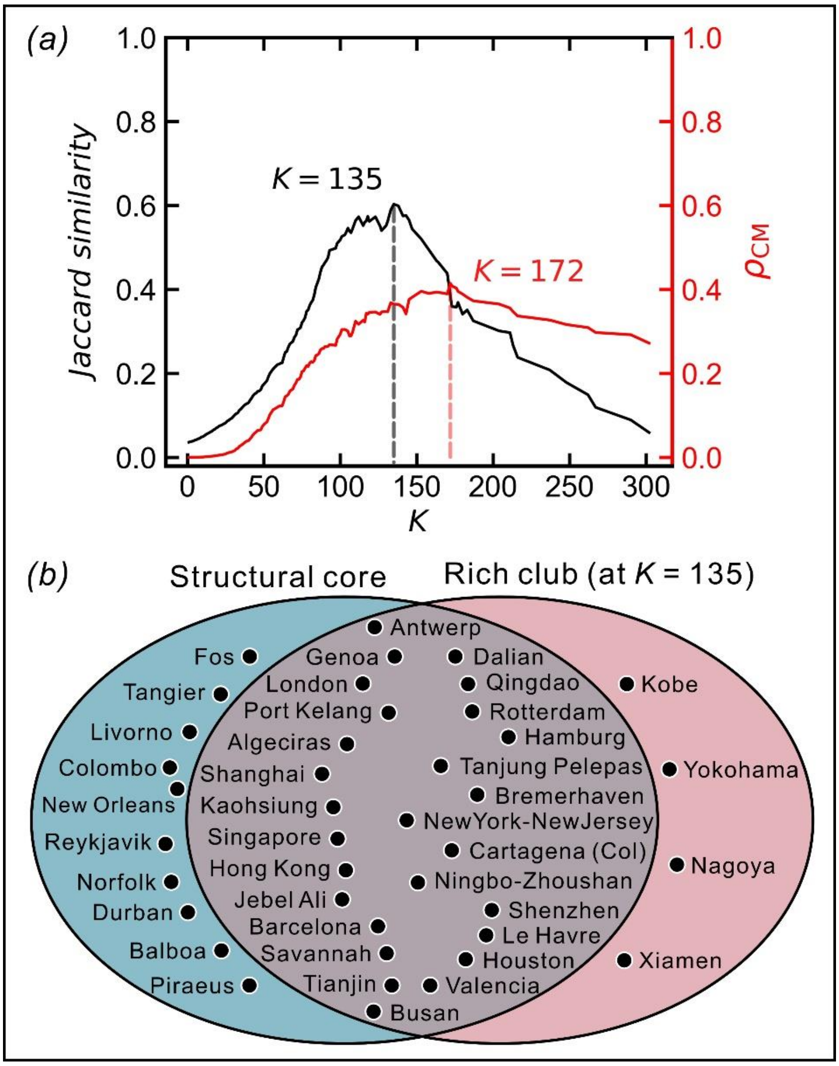

Supplementary Fig. 7 Overlap between the rich club and the structural core of the GLSN. Panel ***(a)*** shows Jaccard similarity between the gateway-hub structural-core ports and the rich-club ports of the GLSN (black line and black y-axis on the left) and ports' rich-clubness ρ_{CM} (red line and red y-axis on the right), with two vertical dash lines indicating the respective peaks of them. Jaccard similarity index is often used for comparing similarity, dissimilarity, and distance between two data sets, defined as the ratio of the number of shared nodes to the number of distinct nodes in the two data sets. It varies between 0 and 1, indicating a lowest level and a highest level of similarity, respectively. For each degree the black line indicates the average Jaccard similarity between the corresponding rich-club ports and the gateway-hub structural-core ports detected in 1,000 iterations. The detection of a gateway-hub structural core is based on the modular community division by the Louvain algorithm (Blondel et al (2008))[8], and this algorithm is non-deterministic. Therefore, we performed 1,000 times this algorithm and repeated the procedure of structural-core detection; in 921 runs, we successfully detected a structural core by using the topological indicator termed gateway-ness. We have shown in the Supplementary Note 5 that the structural-core detection is strongly robust over multiple runs of the Louvain algorithm. In ***(b)*** we show a Venn diagram presenting the rich-club set (in pink) identified at the degree value of 135 (i.e. the peak

point of Jaccard similarity), a structural-core set (in blue) represented in the present study (i.e. the one in Fig. 7b of the main article), and their overlapped components. These results here show that the structural core is not the same as any possible rich club of the GLSN. Source data are provided as a Source Data file.

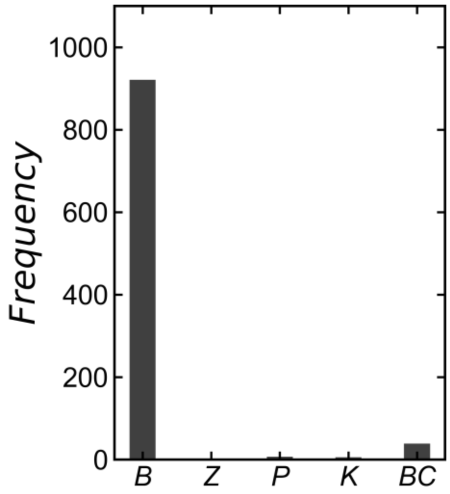

Supplementary Fig. 8 Frequency with which a structural core was detected based on a given topological indicator, over 1,000 realisations. *B*, *Z*, *P*, *K*, and *BC* indicate gatway-ness, provincial-ness, connector-ness, degree, and betweenness centrality, respectively. Note: even in very rare cases a structural core was detected by *Z*, *P*, *K*, or *BC*, it can anyway be detected by *B*.

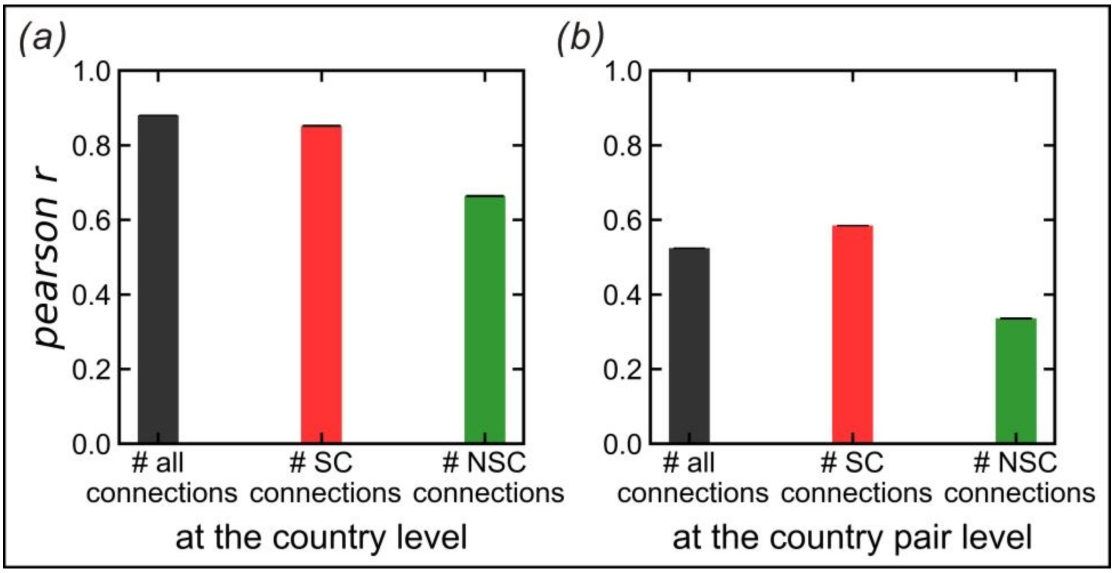

Supplementary Fig. 9 Pearson correlation coefficients between the GLSN topological indicators and international trade indicators of countries, averaged over 1,000 repetitions of the experiment. In panel ***(a)*** we show for countries Pearson correlation coefficients between international trade value (ITV) and # all (inter-port) connections with all other countries in the world (***black bar***); between ITV and # SC connections (structural-core connections, those between

a country's structural-core ports and ports of other countries), ***red bar***; and between ITV and # NSC connections (non-structural-core connections, those between a country's non-structural-core ports and ports of other countries), ***green bar.*** In panel ***(b)*** we show for country pairs the Pearson correlation coefficients between the bilateral trade value (BTV) and # all (inter-port) connections between the two countries (***black bar***); between BTV and # SC connections (structural-core connections, those with at least one end-node of the connection being a structural-core port), ***red bar***; and between BTV and # NSC connections (non-structural-core connections, those with two end-nodes of the connection being both non-structural-core ports), ***green bar***. Note: all the reported Pearson correlation coefficients are mean values over 1,000 runs of the experiment; error bars report the standard errors. Source data are provided as a Source Data file.

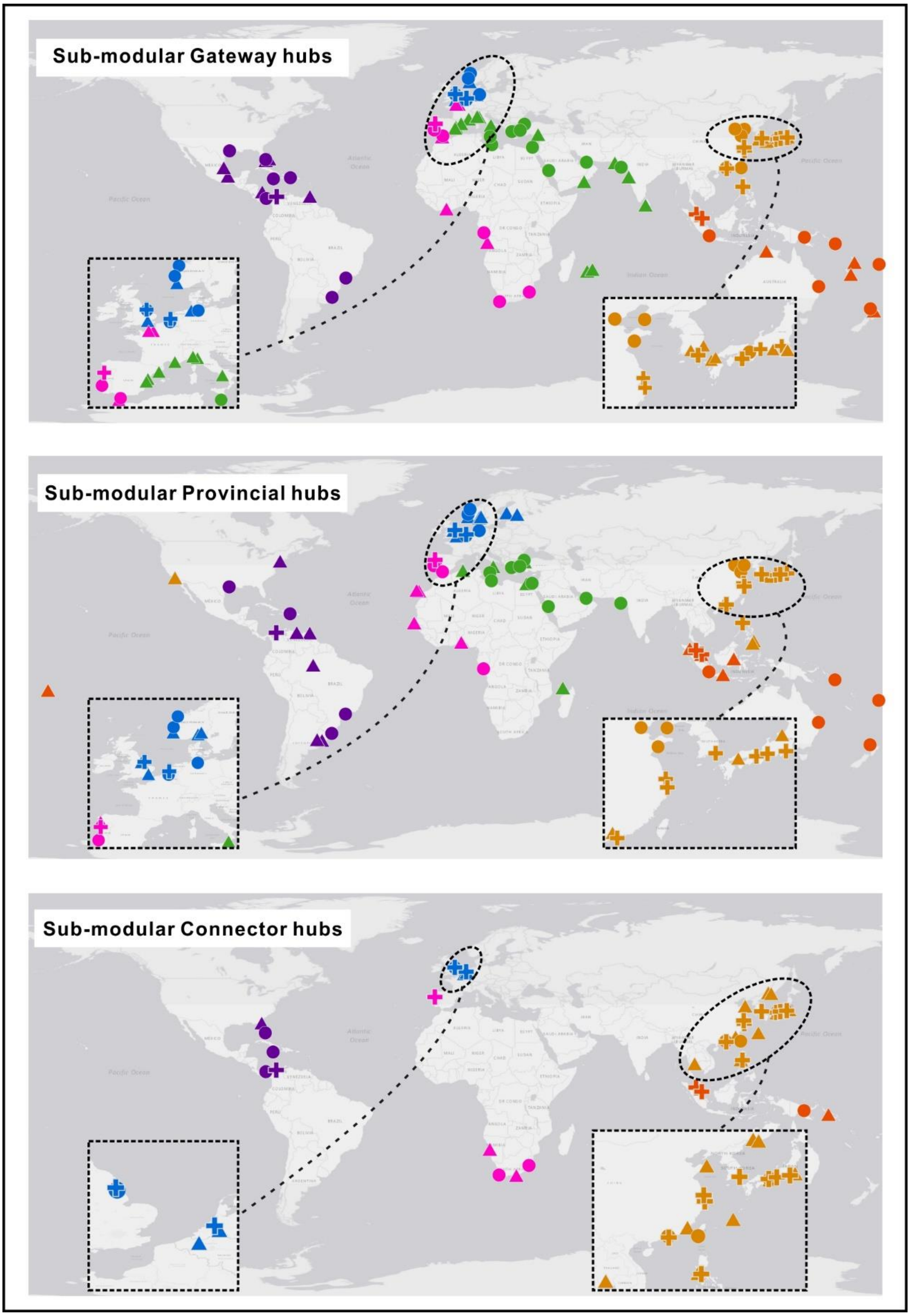

Supplementary Fig. 10 Geographical distribution of submodular gateway hubs, provincial hubs and connector hubs. We extracted from the GLSN each modular sub-network (i.e. ports in the module and connections among them), and then measured each port's submodular gateway-ness, provincial-ness and connector-ness based on its pattern of intra-submodule and inter-submodule links. Submodular provincial hubs refer to ports with inside-submodule degrees at least 1.5

standard deviations above the submodule mean; submodular gateway hubs, ports with outside-submodule degrees at least 1.5 standard deviations above the submodule mean; and submodular connector hubs, ports with modular participation coefficient of at least 0.7. Ports are colored according to modular communities. In each plot, triangles denote ports that are associated with only that particular type of hub role under investigation; circles, ports that play one additional type of hub role; crosses, ports that play all the three types of hub roles. Inside-submodule degrees, outside-submodule degrees, and modular participation coefficients of the hub ports are reported in the source data. Source data are provided as a Source Data file.

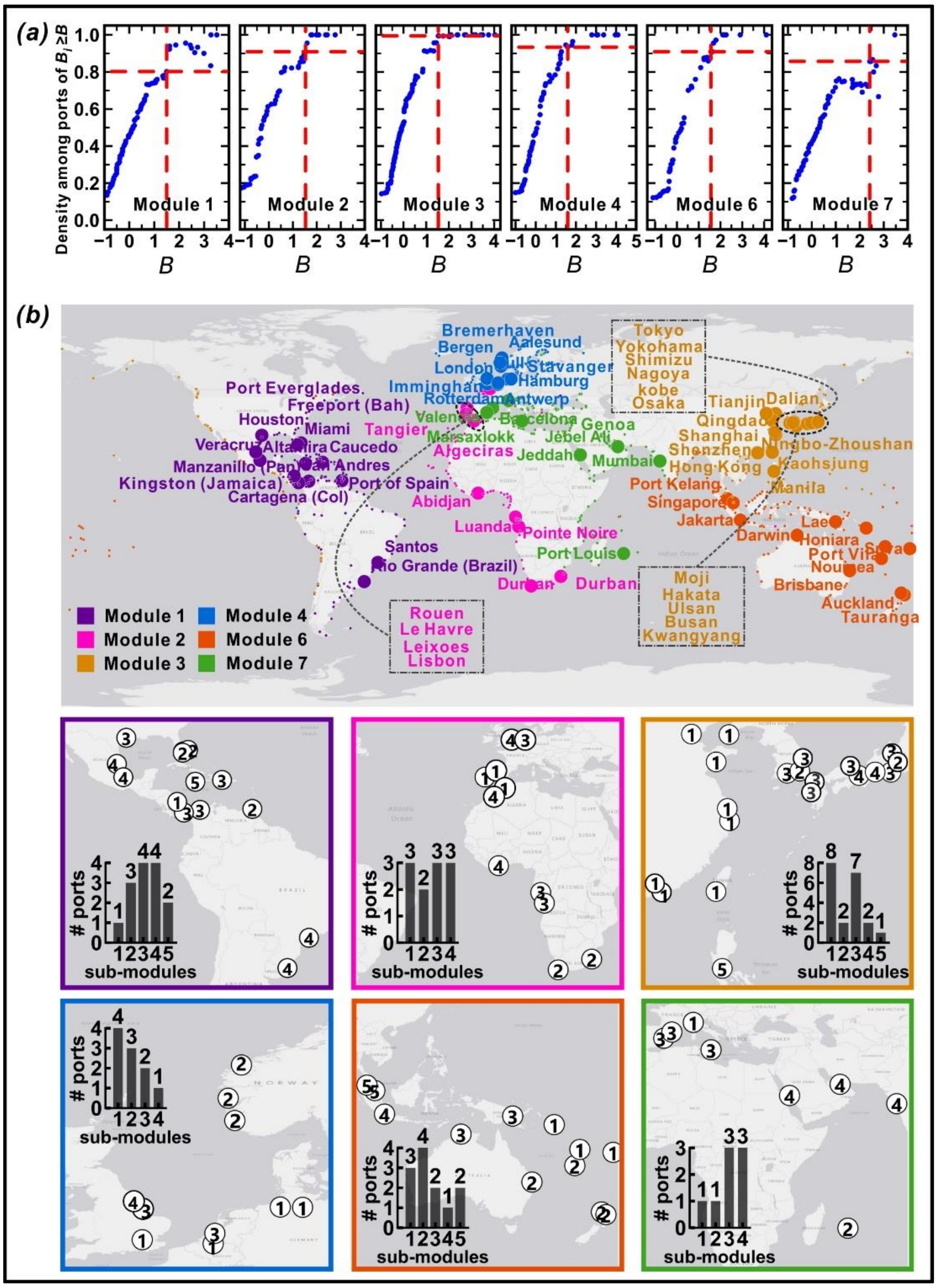

Supplementary Fig. 11 Results for the structural-core detection within individual modules, based on submodular gateway hubs. Plots in upper panel ***(a)*** show for each module of interest the

connection density among ports with largest outside-submodule degrees (*B* values). In each plot the horizontal dash line indicates the connection density among the filtered-out ports and the vertical dash line the corresponding threshold *B* value, under the definition of structural core organization (i.e. the largest number of the most important hub ports that form a subgraph of density at least 0.8). Lower panel ***(b)***: The big plot shows the geographical distribution of the filtered-out ports (big dots), and the small plots present the distribution of these ports over the individual submodules of their respective modules. Note: The smallest module (module 5), which covers the geographical area mainly consisting of Greenland and Iceland, cannot be further divided into submodules.

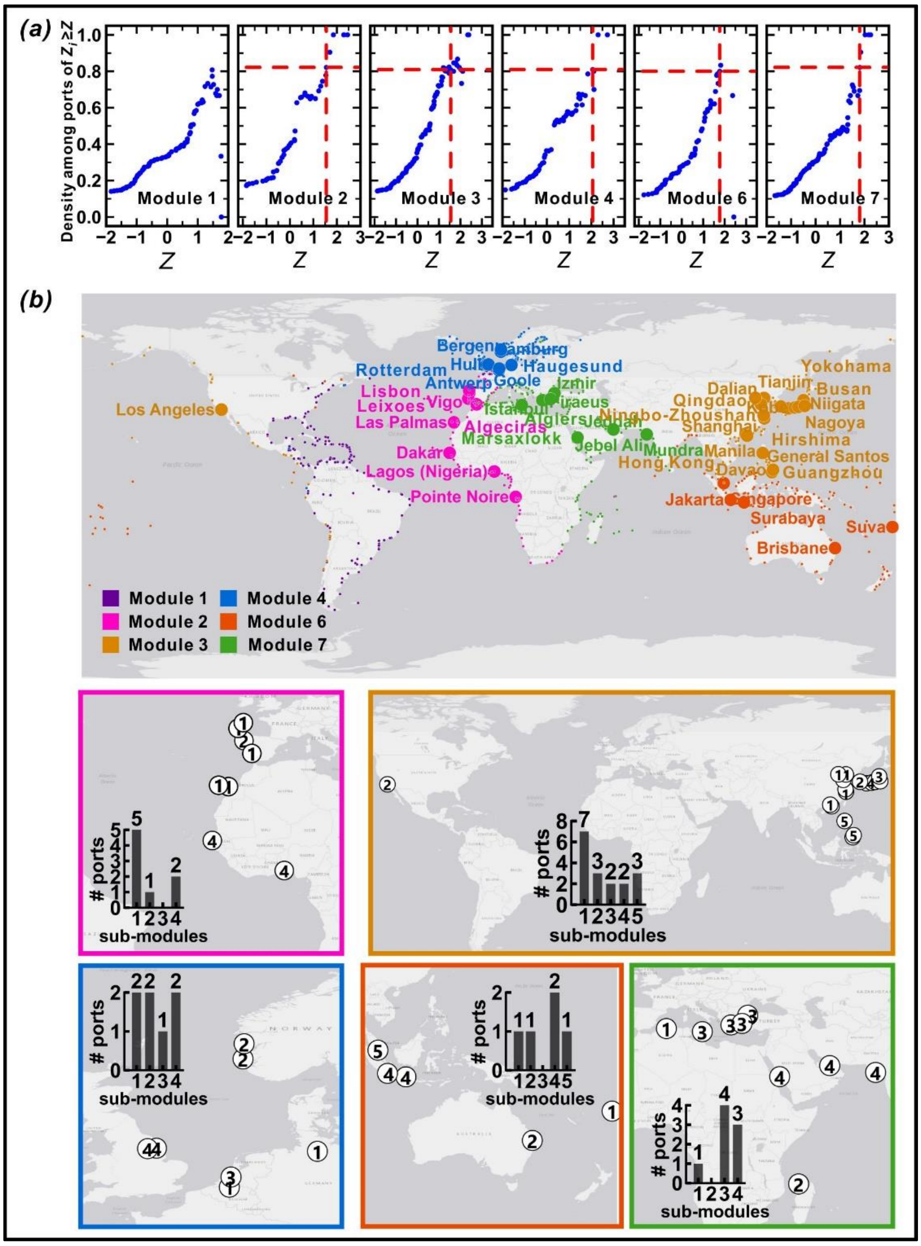

Supplementary Fig. 12 Results for the structural-core detection within individual modules, based on submodular provincial hubs. Plots in upper panel ***(a)*** show for each module of interest the

connection density among ports with largest inside-submodule degrees (*Z* values). In the plot the horizontal dash line indicates the connection density among the filtered-out ports (if exist) and the vertical dash line the corresponding threshold *Z* value, under the definition of structural core organization (i.e. the largest number of the most important hub ports that form a subgraph of density at least 0.8). Lower panel ***(b)***: The big plot shows the geographical distribution of the filtered-out ports (big dots), and the small plots present the distribution of these ports over the individual submodules of their respective modules. Note: The smallest module (module 5), which covers the geographical area mainly consisting of Greenland and Iceland, cannot be further divided into submodules.

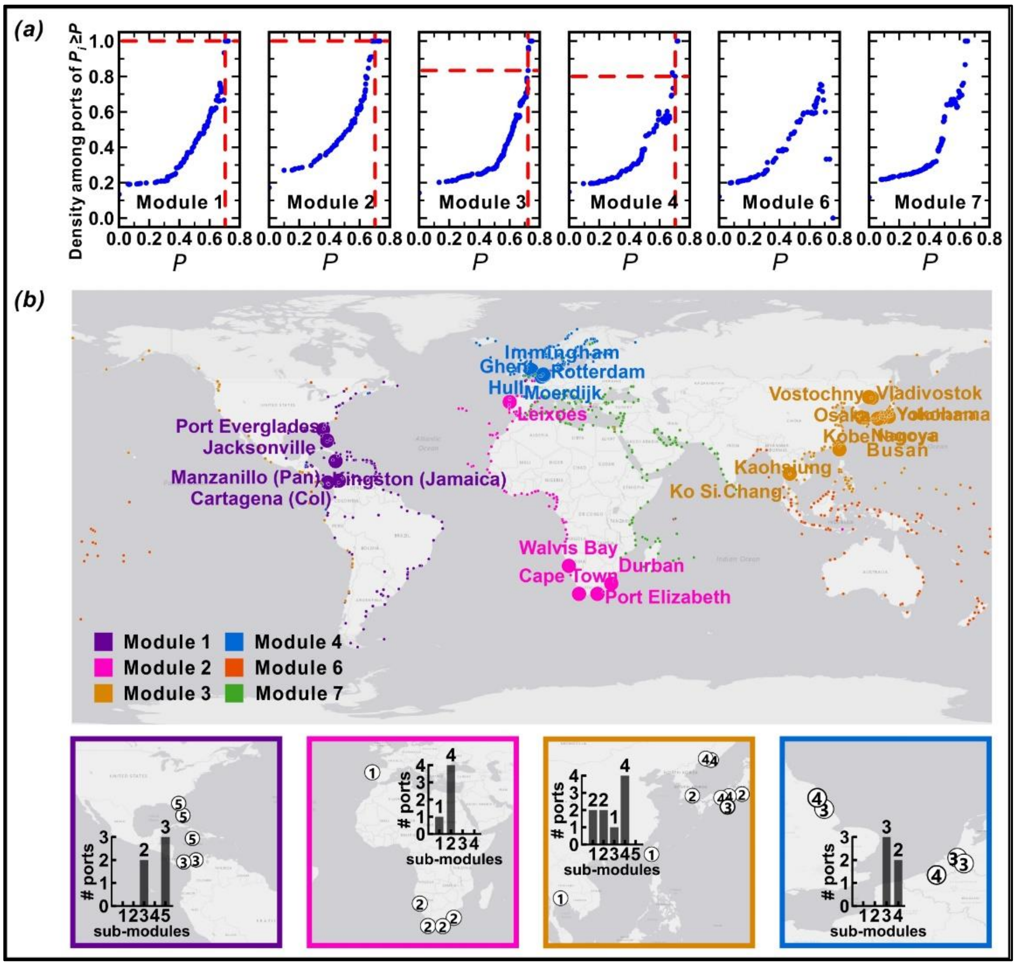

Supplementary Fig. 13 Results for the structural-core detection within individual modules, based on submodular connector hubs. Plots in upper panel ***(a)*** show for each module of interest the connection density among ports with largest modular participation coefficients (*P* values). In the plot the horizontal dash line indicates the connection density among the filtered-out ports (if exist) and the vertical dash line the corresponding threshold *P* value, under the definition of structural core organization (i.e. the largest number of the most important hub ports that form a subgraph of density at least 0.8). Lower panel ***(b)***: The big plot shows the geographical distribution of the filtered-out ports (big dots), and the small plots present the distribution of these ports over the

individual submodules of their respective modules. Note: The smallest module (module 5), which covers the geographical area mainly consisting of Greenland and Iceland, cannot be further divided into submodules.

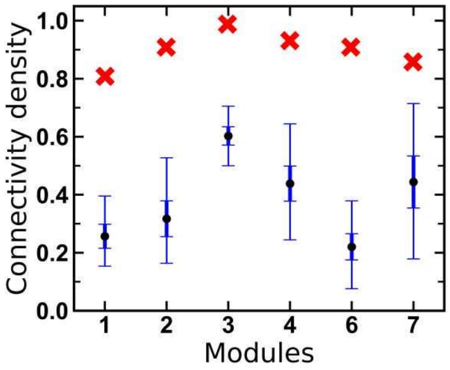

Supplementary Fig. 14 Comparison with permutated networks for the connectivity density among the respective structural-core ports in the studied modules. We introduced a configuration null model where links in the real GLSN topology were randomly rewired and ports' degree sequence were preserved, and then for each module compared the connectivity density of the identified set of submodular gateway-hub ports in the empirical network structure with that in the random case. Empirical results are indicated by red crosses, and random results by blue bars. A blue bar reports the maximum (the highest point), minimum (the lowest point), mean (the dot), and standard deviation (the bar in bold) over 1,000 random cases. Hence, regarding each empirical result for the connectivity density of the structural core ports in the respective module, we got a p-value < 0.001. Note: The smallest module (module 5), which covers the geographical area mainly consisting of Greenland and Iceland, cannot be further divided into submodules. Source data are provided as a Source Data file.

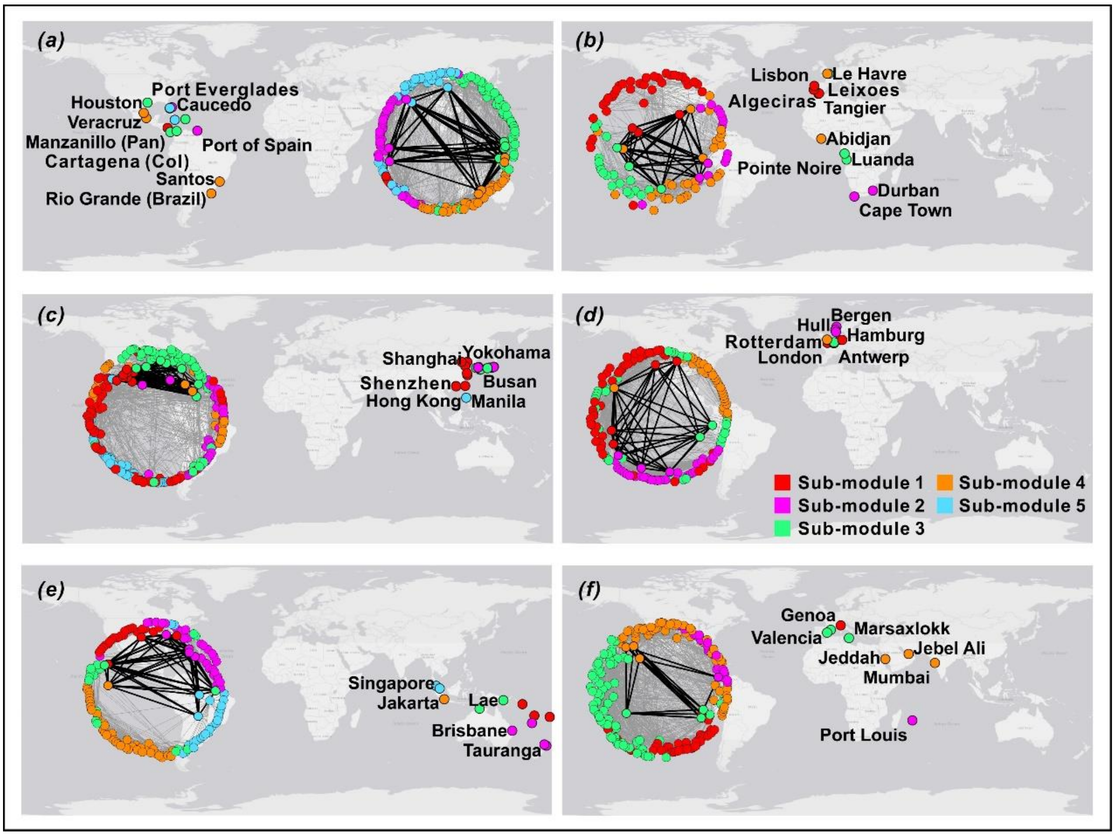

Supplementary Fig. 15 Representation of the modular communities and their respective structural cores in the hyperbolic space. Panel ***(a)***, ***(b)***, ***(c)***, ***(d)***, ***(e)***, and ***(f)*** present results for modules 1, 2, 3, 4, 6, and 7, respectively. The color of the nodes corresponds to the submodular communities. Of the hyperbolic plot in each panel, the nodes belonging to the structural core are highlighted with a thicker black border and the intra-core connections are marked in black, whereas the other connections are in grey. The geographical locations of the structural-core ports are shown in the map.

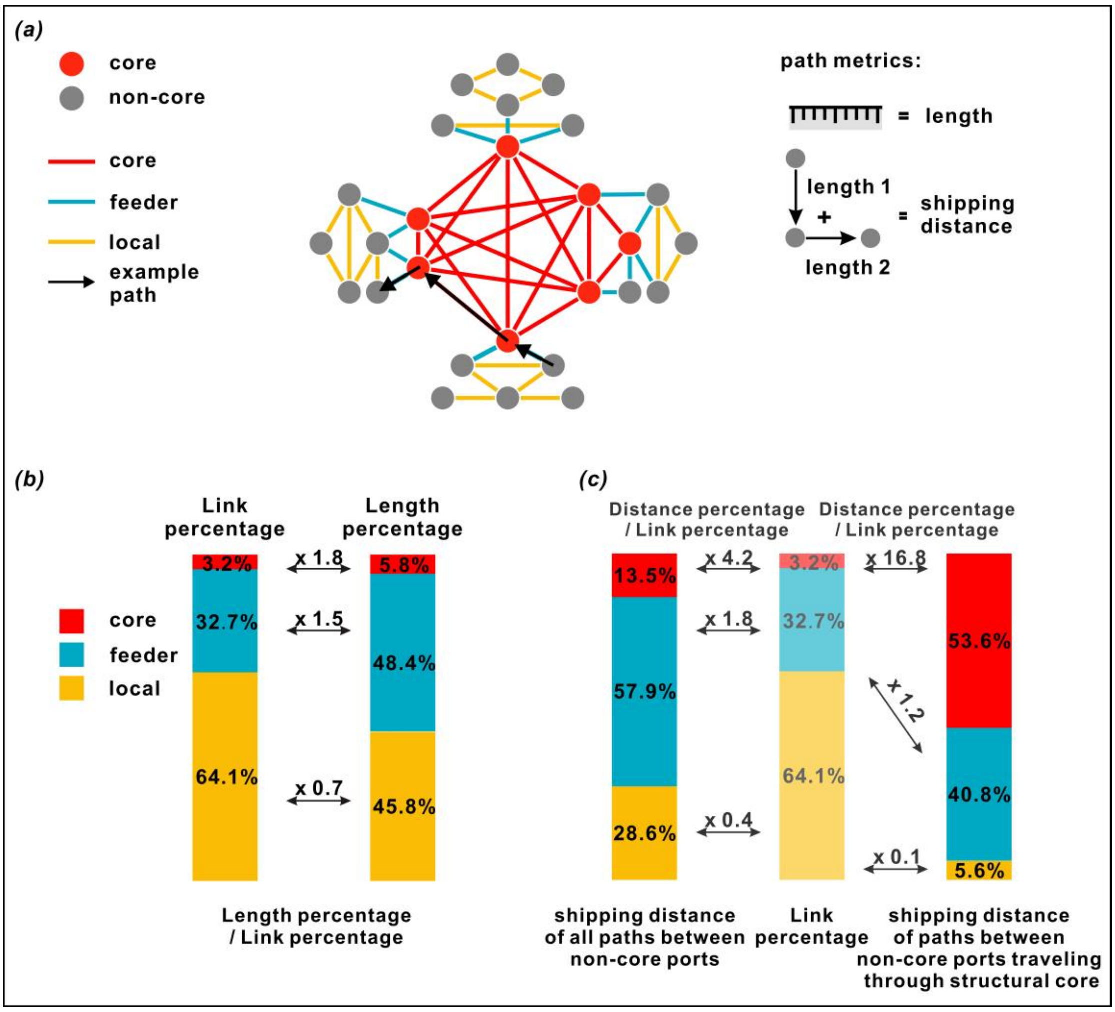

Supplementary Fig. 16 Statistics of the core, feeder and local connections. Panel ***(a)***: Schematic illustration of path metrics; the geographical length of an inter-port connection is measured as the great-circle distance between the two ports, and shipping distance of any port pair is the sum of geographical length of edges along the shortest path. Panel ***(b)***: Ratios of length percentage to link percentage for core connections, feeder connections and local connections, respectively. Panel ***(c)***: Percentages of core connections, feeder connections and local connections in the total shipping distance of all shortest paths between non-core ports (Left bar), and of shortest paths between non-core ports which travel through the structural core (Right bar).

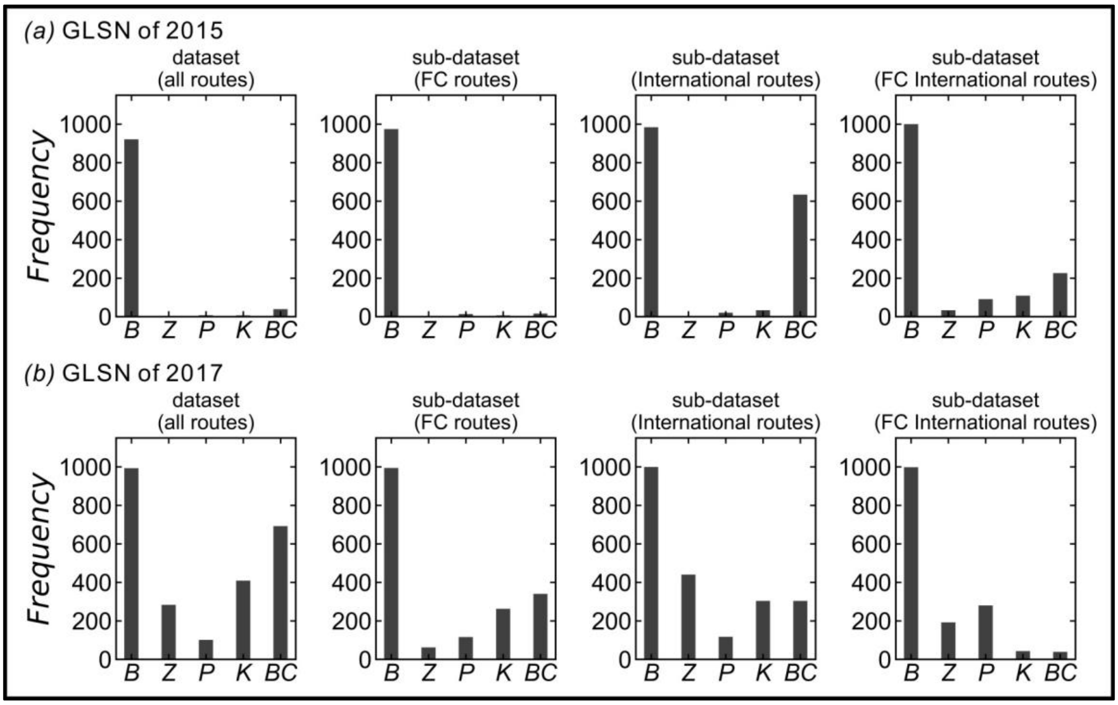

Supplementary Fig. 17 Frequency with which a structural core of the GLSN was detected based on a given topological indicator, across datasets. In ***(a)***, we show the results for the dataset of 2015 and three sub-datasets derived from it; in ***(b)***, the results for the datset of 2017 and its three sub-datasets. *B*, *Z*, *P*, *K*, and *BC* indicate gatway-ness, provincial-ness, connector-ness, degree, and betweenness centrality, respectively. For each dataset and sub-dataset, we repeated the structural-core detection experiment for 1,000 times, considering the non-detrimental property of the Louvain algorithm in modular community division. Note: even in some cases a structural core was detected by *Z*, *P*, *K*, or *BC*, it can anyway be detected by *B*.

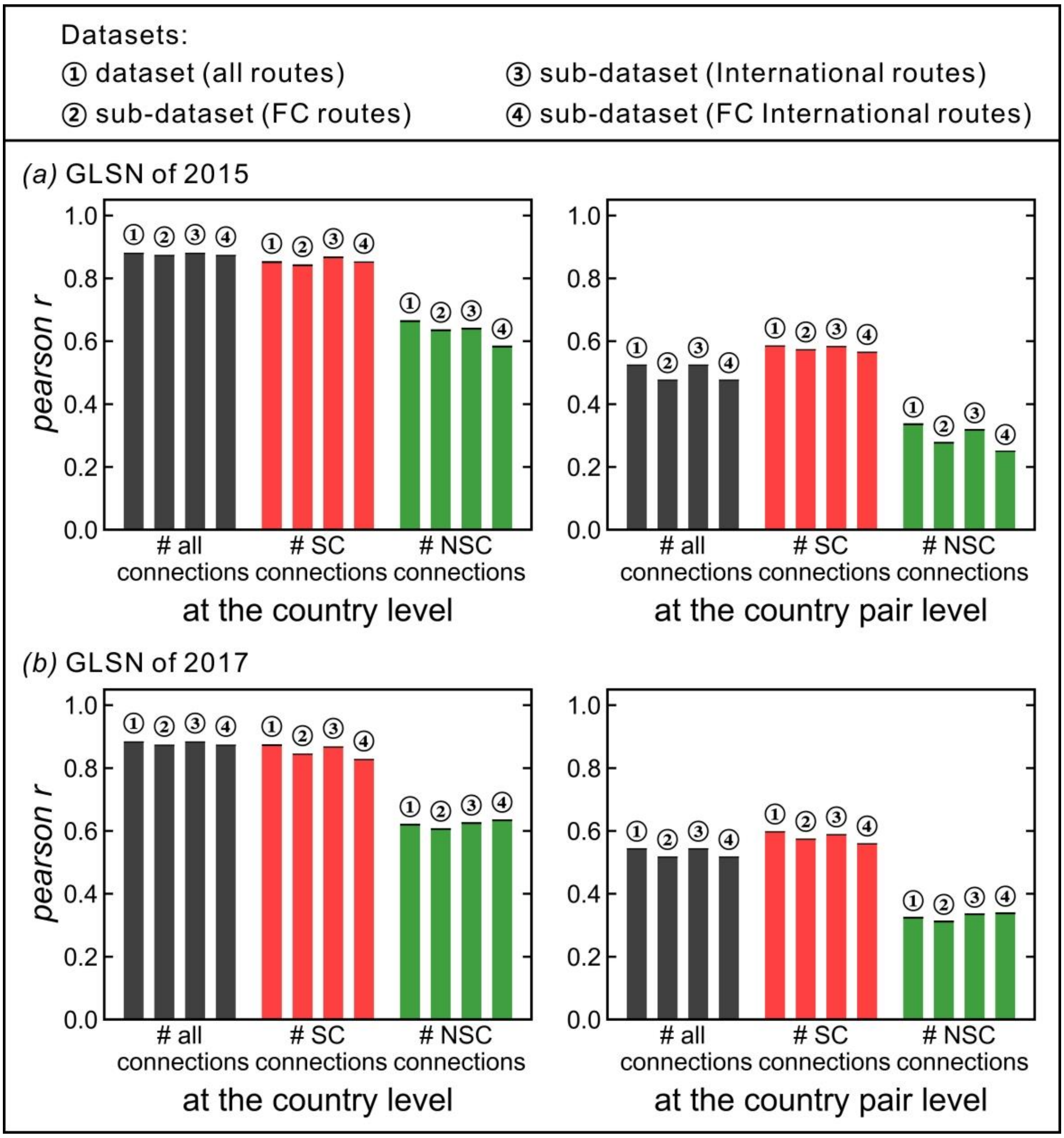

Supplementary Fig. 18 Pearson correlation coefficients between the GLSN topological indicators and international trade indicators of countries, across datasets. For each dataset, the Pearson correlation coefficients reported here are mean values over those repetitions where a structural core is detected (from 1,000 repetitions of the Louvain-algorithm-based community division experiment). Error bars report the standard errors. Panel ***(a)*** and Panel ***(b)*** are results for the GLSN of 2015 and the GLSN of 2017, respectively. In ***(a) left*** and ***(b) left,*** we show for countries Pearson correlation coefficients between international trade value (ITV) and # all (inter-port) connections with all other countries in the world ***(black bars)***; between ITV and # SC connections (structural-core connections, those between a country's structural-core ports and ports of other countries), ***red bars***; and between ITV and # NSC connections (non-structural-core connections, those between a country's non-structural-core ports and ports of other countries), ***green bars.*** In ***(a) right*** and ***(b) right:*** we show for country pairs the Pearson correlation coefficients between the bilateral trade value (BTV) and # all (inter-port) connections between the two countries ***(black bars)***;

between BTV and # SC connections (structural-core connections, those with at least one end-node of the connection being a structural-core port), ***red bars***; and between BTV and # NSC connections (non-structural-core connections, those with two end-nodes of the connection being both non-structural-core ports), ***green bars***. Source data are provided as a Source Data file.

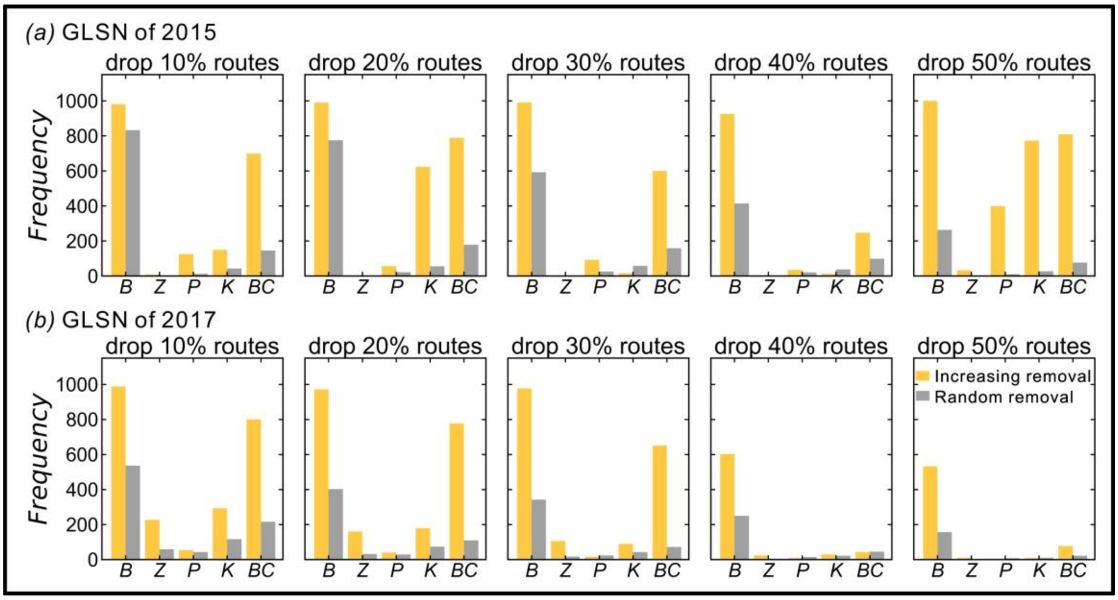

Supplementary Fig. 19 Frequency with which a structural core of the GLSN was detected based on given topological indicators, across removal methods and percent of removed routes. *B*, *Z*, *P*, *K*, and *BC* indicate gatway-ness, provincial-ness, connector-ness, degree, and betweenness centrality, respectively. Results presented in each plot were obtained by 1,000 iterations of the experiment. Specifically, for results regarding increasing removal of shipping routes with smallest traffic capacity, we repeated 1,000 times the Louvain algorithm for modular community division; for results regarding uniform random removal of shipping routes, the uniform random selection of shipping routes to be dropped was sampled 1,000 times. Note: in all cases of increasing removal and random removal, even a structural core was detected by *Z*, *P*, *K*, or *BC*, it can anyway be detected by *B*.

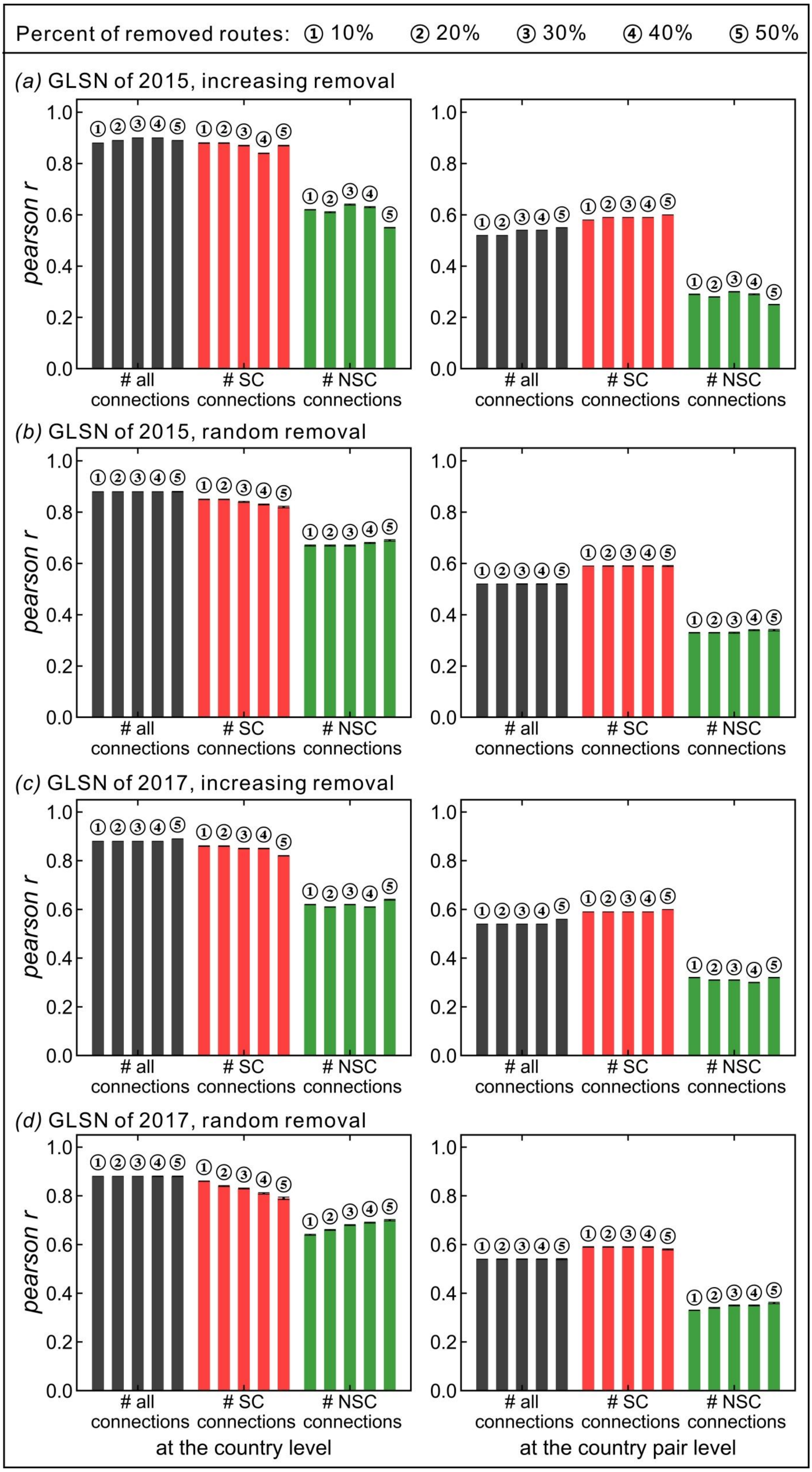

Supplementary Fig. 20 Pearson correlation coefficients between the GLSN topological indicators

and international trade indicators of countries, across removal methods and percent of removed routes. For each data point, the Pearson correlation coefficient reported here is the mean value over those runs where a structural core is detected (from 1,000 runs of the experiment of the gateway-hub structural core detection). Error bars report the standard errors. Panel ***(a)*** are results for the GLSN of 2015 when we increasingly removed between 10%, 20%, 30%, 40%, and 50% of shipping routes with smallest traffic capacity from the original dataset, and panel ***(b)*** are results for this network when we removed the same percent of shipping routes uniformly at random. Likewise, panel ***(c)*** and panel ***(d)*** are results for the GLSN of 2017. In ***(a) left, (b) left, (c) left,*** and ***(d) left,*** we show for countries Pearson correlation coefficients between international trade value (ITV) and # all (inter-port) connections with all other countries in the world ***(black bars)***; between ITV and # SC connections (structural-core connections, those between a country's structural-core ports and ports of other countries), ***red bars***; and between ITV and # NSC connections (non-structural-core connections, those between a country's non-structural-core ports and ports of other countries), ***green bars.*** In ***(a) right, (b) right, (c) right,*** and ***(d) right:*** we show for country pairs the Pearson correlation coefficients between the bilateral trade value (BTV) and # all (inter-port) connections between the two countries ***(black bars)***; between BTV and # SC connections (structural-core connections, those with at least one end-node of the connection being a structural-core port), ***red bars***; and between BTV and # NSC connections (non-structural-core connections, those with two end-nodes of the connection being both non-structural-core ports), ***green bars***. Source data are provided as a Source Data file.

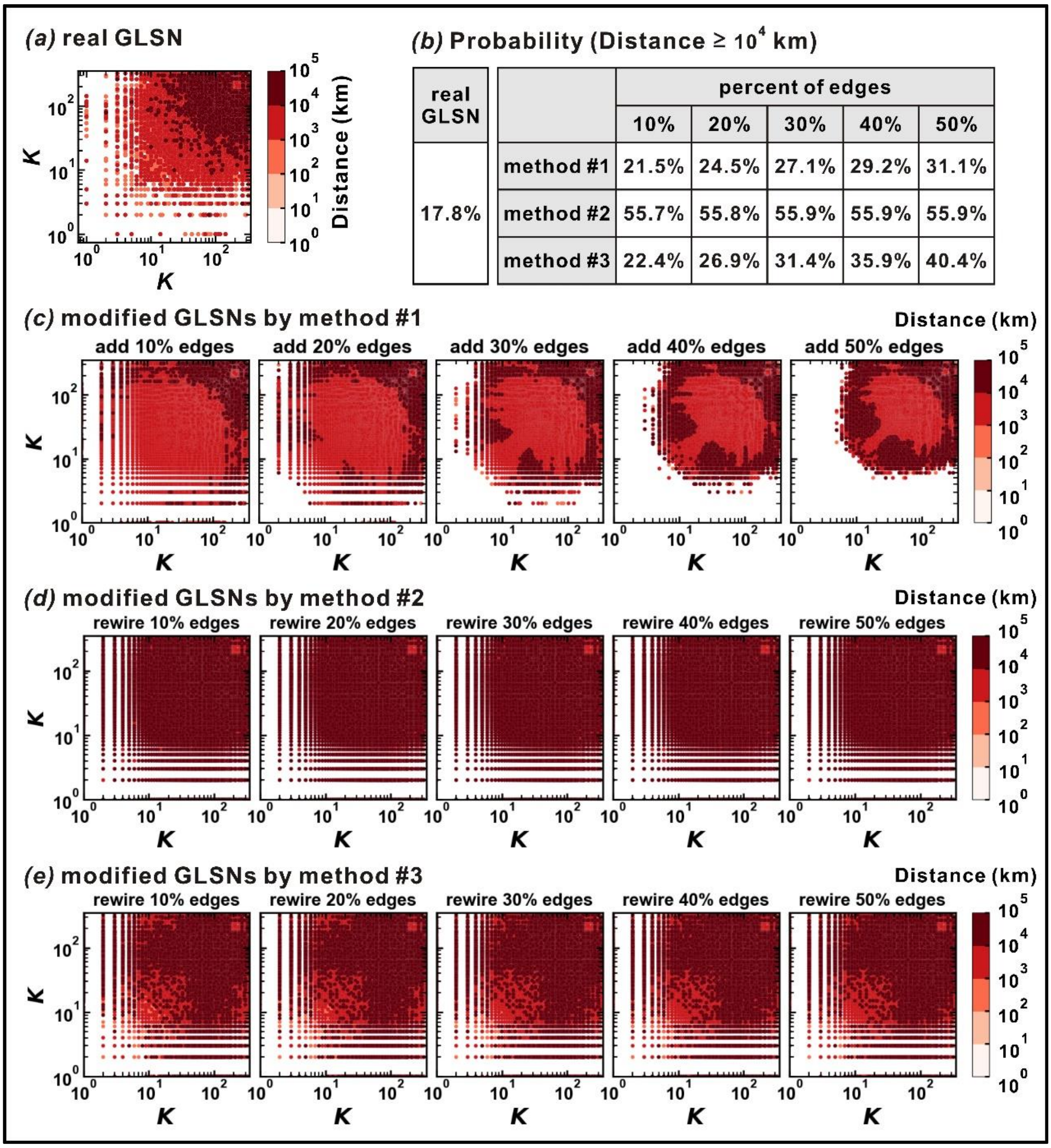

real GLSN		percent of edges				
		10%	20%	30%	40%	50%
17.8%	method #1	21.5%	24.5%	27.1%	29.2%	31.1%
	method #2	55.7%	55.8%	55.9%	55.9%	55.9%
	method #3	22.4%	26.9%	31.4%	35.9%	40.4%

Supplementary Fig. 21 Edge distributions in the real GLSN of 2015 and its modified networks. Matrix plots represent the edge distributions in the real GLSN of 2015 ***(a)*** and the modified GLSNs (***(c), (d), (e)***), with color red indicating a pair of ports are linked and color white unlinked; the stronger the red color, the longer (in geographical distance) of the edge is. In each matrix plot, ports are sorted in an ascending order of degree (*K*). For method #1 ***(c)***, we uniformly randomly added into the original GLSN between 10%, 20%, 30%, 40%, and 50% of the amount of edges in this network. For method #2 ***(d)*** and method #3 ***(e)***, we randomly rewired between 10%, 20%, 30%, 40%, and 50% of the edges in the original GLSN based on two edge-rewiring processes proposed in reference[7] and reference[6], respectively. In ***(b)*** we show the probabilities of long-distance edges (i.e., those longer than 10,000 km) in the real and modified GLSNs. With all three methods**,** the geographical restriction of the real GLSN was indeed modified in either or both of the following two ways: one is that long-haul connections would be much more common in modified networks

than in the real GLSN; another is that in modified networks long-haul connections would involve many small ports, whereas in the real GLSN they are quite restricted to big ports.

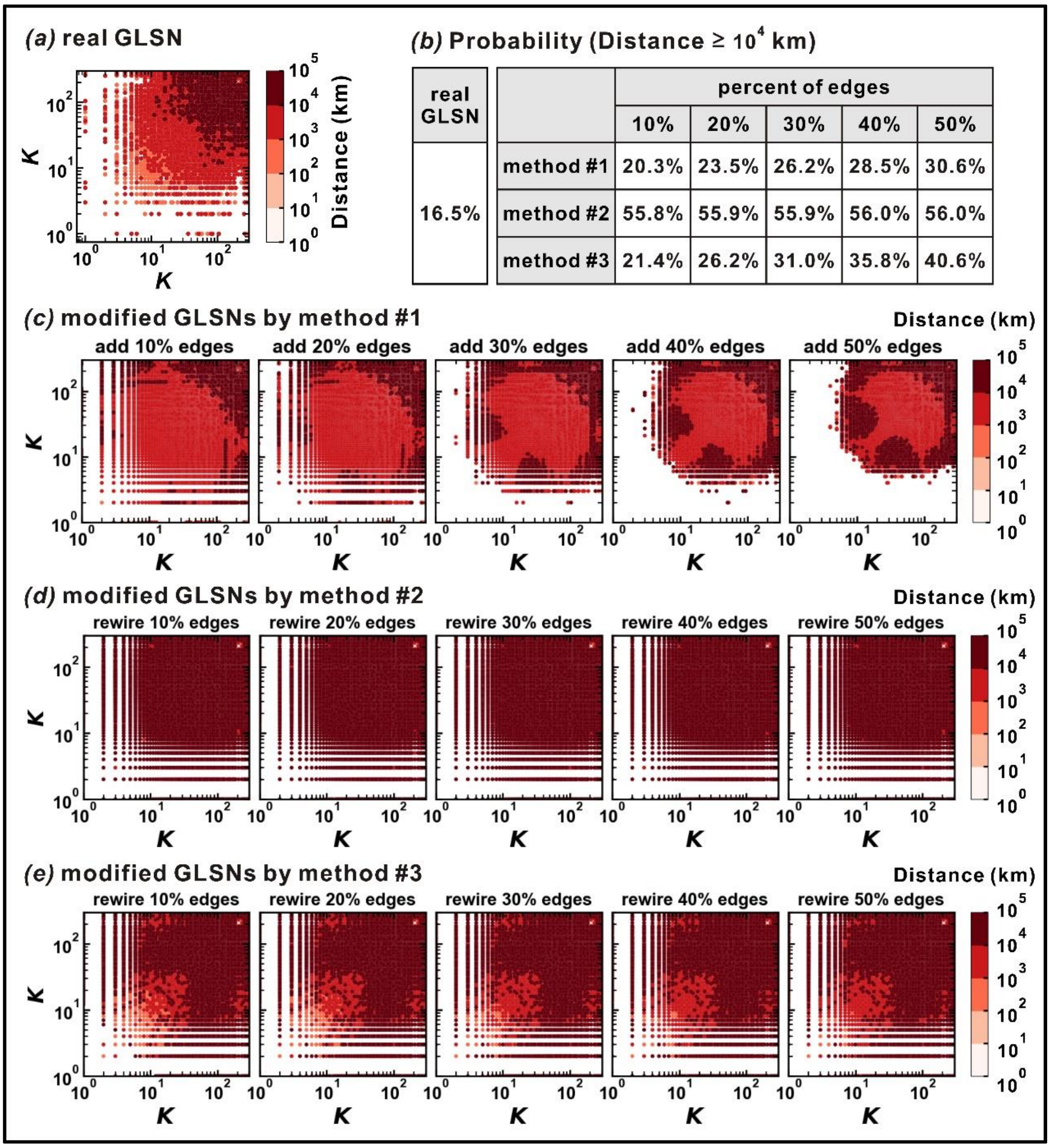

real GLSN		percent of edges				
		10%	20%	30%	40%	50%
16.5%	method #1	20.3%	23.5%	26.2%	28.5%	30.6%
	method #2	55.8%	55.9%	55.9%	56.0%	56.0%
	method #3	21.4%	26.2%	31.0%	35.8%	40.6%

Supplementary Fig. 22 Edge distributions in the real GLSN of 2017 and its modified networks. Matrix plots represent the edge distributions in the real GLSN of 2017 ***(a)*** and the modified GLSNs (***(c), (d), (e)***), with color red indicating a pair of ports are linked and color white unlinked; the stronger the red color, the longer (in geographical distance) of the edge is. In each matrix plot, ports are sorted in an ascending order of degree (*K*). For method #1 ***(c)***, we uniformly randomly added into the original GLSN between 10%, 20%, 30%, 40%, and 50% of the amount of edges in this network. For method #2 ***(d)*** and method #3 ***(e)***, we randomly rewired between 10%, 20%, 30%, 40%, and 50% of the edges in the original GLSN based on two edge-rewiring processes proposed in reference[7] and reference[6], respectively. In ***(b)*** we show the probabilities of long-distance edges

(i.e., those longer than 10,000 km) in the real and modified GLSNs. With all three methods, the geographical restriction of the real GLSN was indeed modified in either or both of the following two ways: one is that long-haul connections would be much more common in modified networks than in the real GLSN; another is that in modified networks long-haul connections would involve many small ports, whereas in the real GLSN they are quite restricted to big ports.

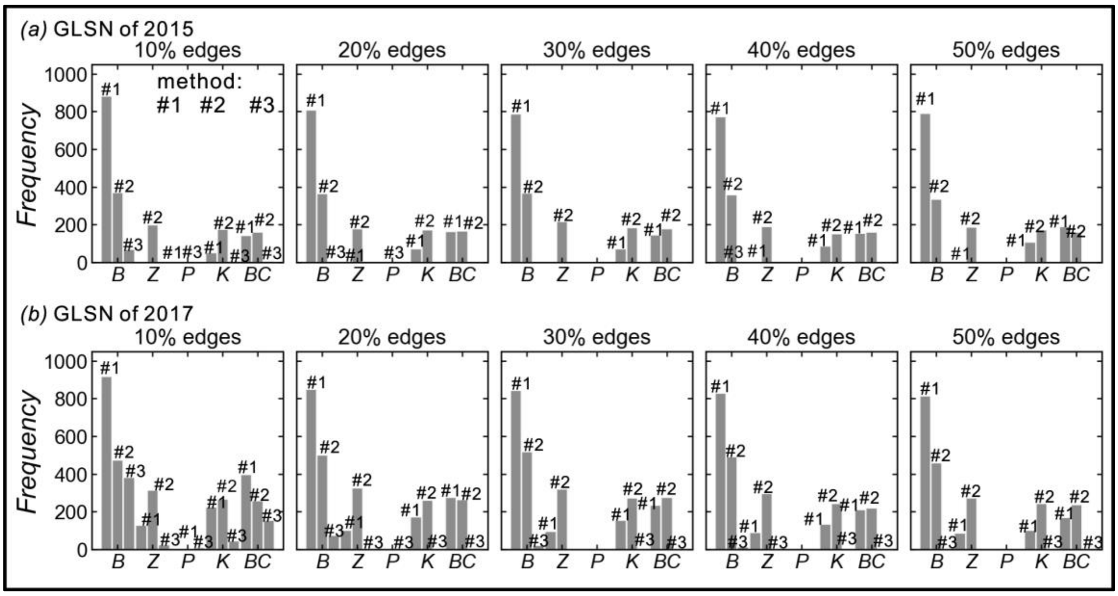

Supplementary Fig. 23 Frequency with which a structural core of the GLSN was detected based on a given topological indicator, across modification methods and percent of edges. *B*, *Z*, *P*, *K*, and *BC* indicate gatway-ness, provincial-ness, connector-ness, degree, and betweenness centrality, respectively. Results presented in each plot were obtained by 1,000 repetitions of the corresponding experiments of modification. For method #1, we uniformly randomly added into the original GLSN between 10%, 20%, 30%, 40%, and 50% of the amount of edges in this network. For method #2 and method #3, we randomly rewired between 10%, 20%, 30%, 40%, and 50% of the edges in the original GLSN based on two edge-rewiring processes proposed in reference[7] and reference[6], respectively. Note: in all cases, even a structural core was detected by *Z*, *P*, *K*, or *BC*, it can anyway be detected by *B*.

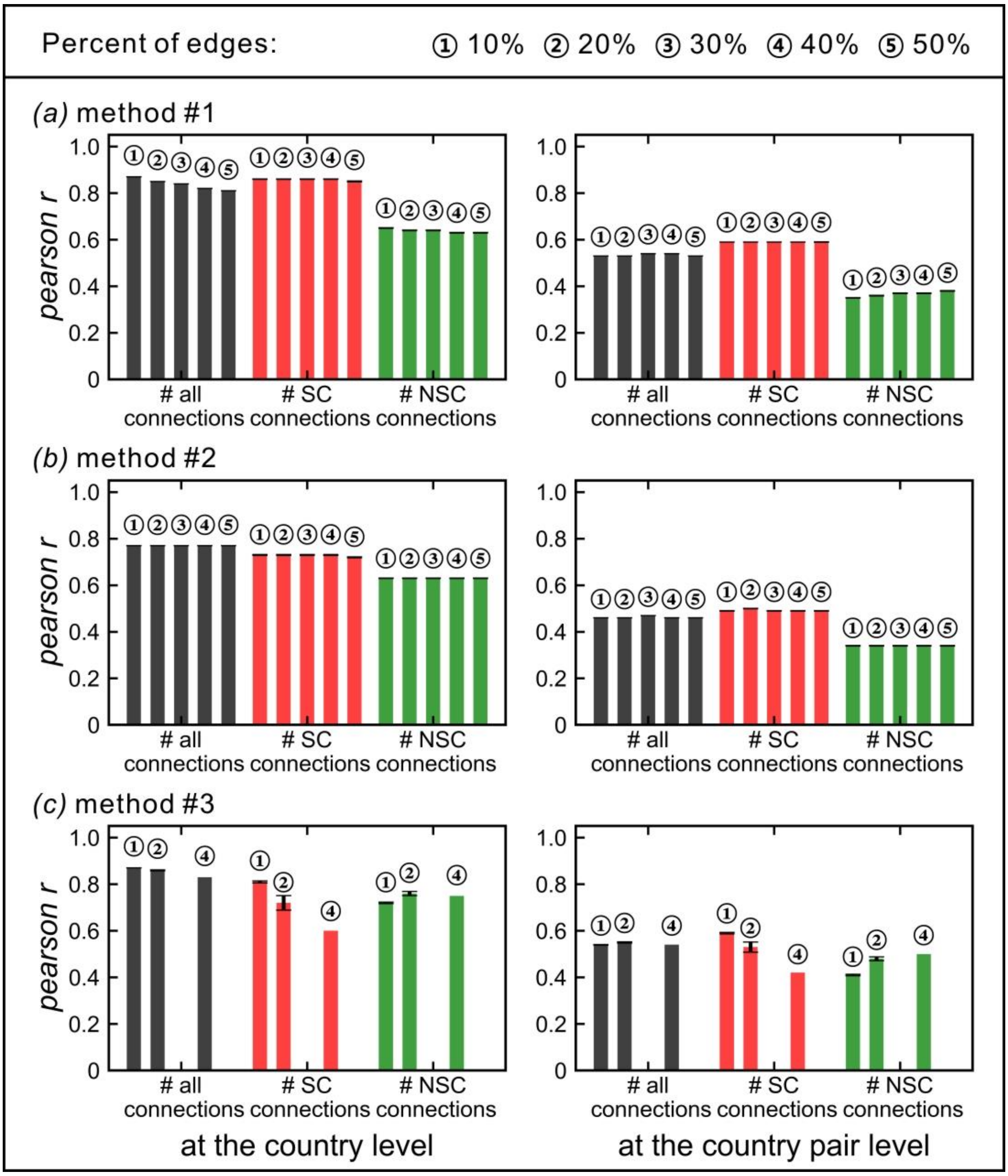

Supplementary Fig. 24 Pearson correlation coefficients between the GLSN topological indicators and international trade indicators of countries (across modification methods and percent of edges), based on 2015 dataset. For each data point, the Pearson correlation coefficient reported here is the mean value over those repetitions where a structural core is detected (from 1,000 repetitions of the corresponding experiments of modification). Error bars report the standard errors. Panel ***(a)***, ***(b)***, and ***(c)*** are results for the modification methods #1, #2, and #3, respectively. In ***(a) left***, ***(b) left***, and ***(c) left,*** we show for countries Pearson correlation coefficients between international trade value (ITV) and # all (inter-port) connections with all other countries in the world ***(black bars)***; between ITV and # SC connections (structural-core connections, those between a country's structural-core ports and ports of other countries), ***red bars***; and between ITV and # NSC connections (non-structural-core connections, those between a country's non-structural-core

ports and ports of other countries), ***green bars.*** In ***(a) right, (b) right,*** and ***(c) right***: we show for country pairs the Pearson correlation coefficients between the bilateral trade value (BTV) and # all (inter-port) connections between the two countries ***(black bars)***; between BTV and # SC connections (structural-core connections, those with at least one end-node of the connection being a structural-core port), ***red bars***; and between BTV and # NSC connections (non-structural-core connections, those with two end-nodes of the connection being both non-structural-core ports), ***green bars***. Source data are provided as a Source Data file.

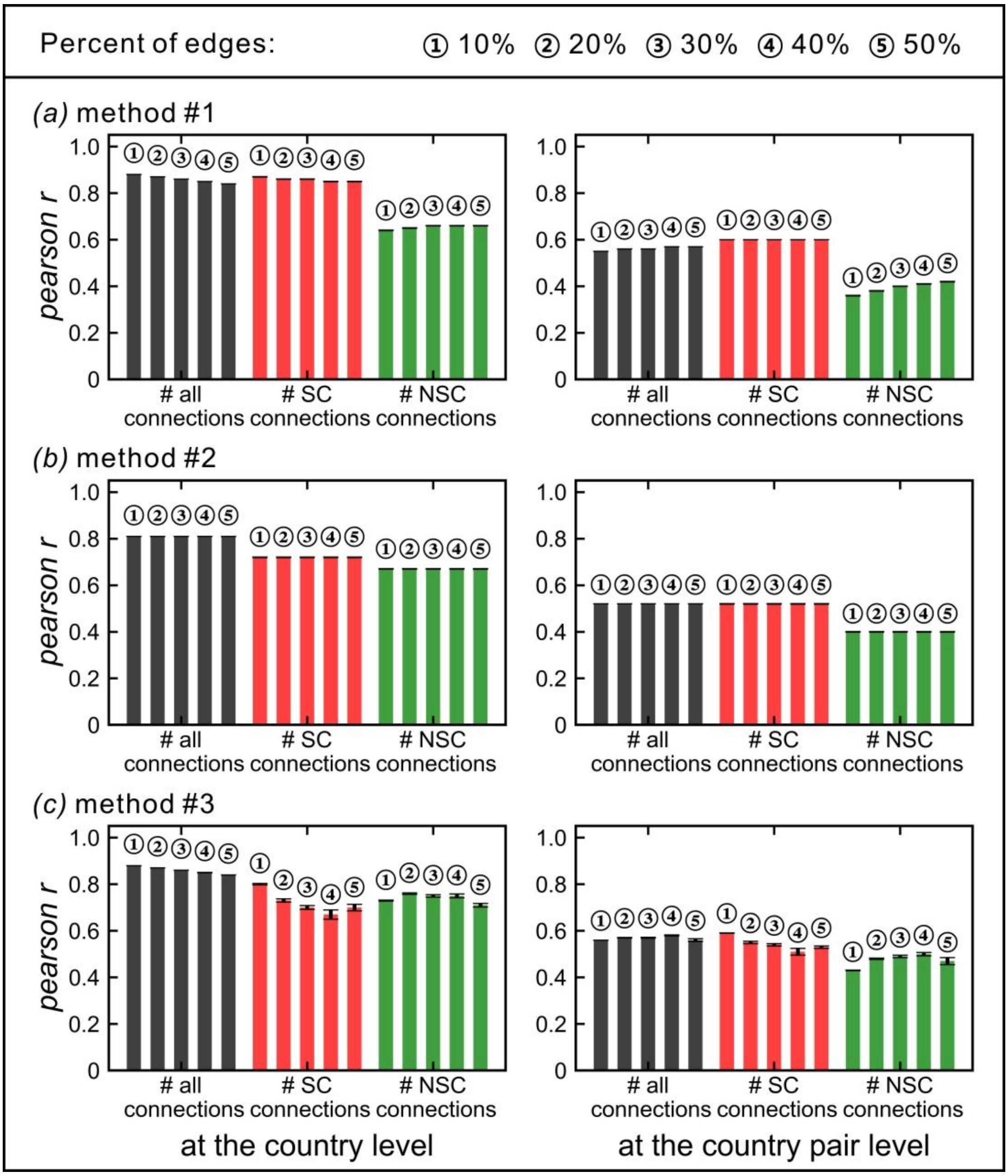

Supplementary Fig. 25 Pearson correlation coefficients between the GLSN topological indicators and international trade indicators of countries (across modification methods and percent of edges), based on 2017 dataset. For each data point, the Pearson correlation coefficient reported

here is the mean value over those repetitions where a structural core is detected (from 1,000 repetitions of the corresponding experiments of modification). Error bars report the standard errors. Panel ***(a), (b),*** and ***(c)*** are results for the modification methods #1, #2, and #3, respectively. In ***(a) left***, ***(b) left***, and ***(c) left,*** we show for countries Pearson correlation coefficients between international trade value (ITV) and # all (inter-port) connections with all other countries in the world ***(black bars)***; between ITV and # SC connections (structural-core connections, those between a country's structural-core ports and ports of other countries), ***red bars***; and between ITV and # NSC connections (non-structural-core connections, those between a country's non-structural-core ports and ports of other countries), ***green bars.*** In ***(a) right, (b) right,*** and ***(c) right:*** we show for country pairs the Pearson correlation coefficients between the bilateral trade value (BTV) and # all (inter-port) connections between the two countries ***(black bars)***; between BTV and # SC connections (structural-core connections, those with at least one end-node of the connection being a structural-core port), ***red bars***; and between BTV and # NSC connections (non-structural-core connections, those with two end-nodes of the connection being both non-structural-core ports), ***green bars***. Source data are provided as a Source Data file.

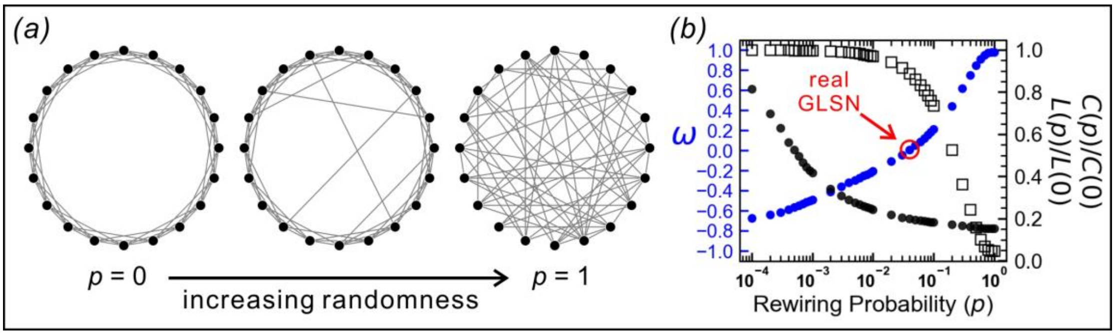

Supplementary Fig. 26 Small-world networks generated by adoption of the Watts-Strogatz (WS) model[9], corresponding to the real GLSN of 2015. Small-world-ness (ω) behaves as required on the WS model of small-world networks. ***(a)*** The WS model begins with a ring of n nodes (here n = 977, i.e. the number of nodes in the real GLSN), each node being connected to its nearest neighbors out to some range K (here K = 34, i.e. the average degree of the real GLSN). Each edge in turn is rewired to a new target node with probability p. ***(b)*** The WS model shows that p = 0 gives a regular network, with high clustering but high characteristic path length; p = 1 gives a random network, with low clustering and characteristic path length; intermediate p values give small-world networks with high clustering and low characteristic path length. Squares and circles indicate the normalized clustering coefficient and the normalized characteristic path length of rewired networks, respectively. The ω metric[10] (blue dots) traces the small-world behavior of the networks generated at varying rewiring probabilities (p): a value close to 0 represents a small-world network; a positive value, a network closer to a random one; a negative value, a network closer to a lattice. The red circle indicates the ω value of the real GLSN. Note that for each p, the numerical result obtained on each data point is the mean value over 1,000 realizations. Source data are provided as a Source Data file.

Supplementary Tables

Supplementary Table 1 Summary of data on port centrality measures for structural-core ports in the GLSN

Ports		Degree	Betweenness	Closeness
Module 1	Savannah	*	*	*
	Houston	*	*	*
	NewYork-NewJersey	*	*	*
	Livorno	*		
	New Orleans	*	*	*
	Norfolk	*		*
	Cartagena (Col)	*	*	*
	Balboa	*	*	*
Module 2	Le Havre	*	*	*
	Algeciras	*	*	*
	Tangier	*	*	*
	Durban	*	*	*
Module 3	Shanghai	*	*	*
	Ningbo-Zhoushan	*	*	*
	Busan	*	*	*
	Shenzhen	*	*	*
	Hong Kong	*	*	*
	Qingdao	*	*	*
	Tianjin	*	*	*
	Dalian	*	*	*
	Kaohsiung	*	*	*
Module 4	Antwerp	*	*	*
	Rotterdam	*	*	*
	Hamburg	*	*	*
	London	*	*	*
	Bremerhaven	*	*	*
Module 5	Reykjavik		*	
Module 6	Singapore	*	*	*
	Tanjung Pelepas	*	*	*
	Port Kelang	*	*	*
Module 7	Valencia	*	*	*
	Genoa	*	*	*
	Barcelona	*	*	*

	Colombo	*	*	*
	Jebel Ali	*	*	*
	Fos	*		*
	Piraeus	*	*	*

Note: Structural-core ports are listed by module, and their topological centralities are measured by three basic node centrality indicators—degree, betweenness and closeness. An asterisk (*) implies that the respective port ranks in the top 10th percentile among world ports on a given network measure.

Supplementary Table 2 Pearson correlation coefficients between network indicators and port capacity, averaged over 1,000 repetitions of the experiment

Network Indicators	GLSN	Communities
B	0.74<br>(0.001)	0.88<br>(0.000)
Z	0.47<br>(0.001)	0.57<br>(0.001)
P	0.39<br>(0.000)	0.46<br>(0.001)
K	0.77<br>(0.000)	0.87<br>(0.000)
BC	0.68<br>(0.000)	0.82<br>(0.001)
φ	0.62<br>(0.000)	0.80<br>(0.000)
ρ_{C}	0.26<br>(0.000)	0.55<br>(0.001)
ρ_{CM}	0.58<br>(0.000)	0.79<br>(0.001)

Note: Network indicators *B*, *Z, P, K,* and *BC* denote gateway-ness, provincial-ness, connector-ness, degree and betweenness centrality, respectively; φ, the unnormalized rich-club coefficient originally proposed in the reference[4]; ρ_{C} and ρ_{CM}, two normalized versions of the rich-club coefficient that are proposed in reference[5] and in reference[6], respectively. The Pearson correlation coefficient between each network indicator and port capacity is calculated for all ports in the GLSN, and also separately for ports in individual communities; for the latter, the result is averaged over all communities in the GLSN. For each data point, we report the average Pearson correlation coefficient over 1,000 repetitions of the experiment. Standard errors are indicated in brackets. Source data are provided as a Source Data file.

Supplementary Table 3 Summary of data on port centrality measures for structural-core ports within individual modules

Ports			Degree	Betweenness	Closeness
Module 1	Sub-module 1	San Andres			
	Sub-module 2	Freeport (Bah)	*	*	*
		Miami	*	*	*
		Port of Spain	*	*	*
	Sub-module 3	Cartagena (Col)	*	*	*
		Houston	*	*	*
		Manzanillo (Pan)	*	*	*
		Caucedo	*	*	*
	Sub-module 4	Altamira	*		*
		Veracruz			*
		Santos	*		*
		Rio Grande (Brazil)	*	*	
	Sub-module 5	Port Everglades	*	*	*
		Kingston (Jamaica)	*	*	*
Module 2	Sub-module 1	Leixoes	*	*	*
		Lisbon	*	*	*
		Algeciras	*	*	*
	Sub-module 2	Durban		*	
		Cape Town			
	Sub-module 3	Pointe Noire	*	*	*
		Luanda	*	*	*
		Rouen		*	
	Sub-module 4	Abidjan	*	*	*
		Tangier	*		*
		Le Havre		*	*
Module 3	Sub-module 1	Shanghai	*	*	*
		Ningbo-Zhoushan	*	*	*
		Hong Kong	*	*	*
		Tianjin	*	*	*
		Kaohsiung	*	*	*
		Qingdao	*	*	*
		Dalian	*		*
		Shenzhen	*	*	*
	Sub-module 2	Busan	*	*	*

		Yokohama	*	*	*
	Sub-module 3	Osaka	*	*	*
		Tokyo	*		*
		Kwangyang	*	*	*
		Ulsan	*		*
		Hakata	*		*
		Moji	*		*
		Shimizu	*		*
	Sub-module 4	Nagoya	*	*	*
		Kobe	*	*	*
	Sub-module 5	Manila		*	
Module 4	Sub-module 1	Hamburg	*	*	*
		Bremerhaven	*	*	*
		Antwerp	*	*	*
		London	*	*	*
	Sub-module 2	Stavanger	*		*
		Bergen	*	*	*
		Aalesund	*	*	*
	Sub-module 3	Rotterdam	*	*	*
		Immingham	*	*	*
	Sub-module 4	Hull	*	*	*
Module 6	Sub-module 1	Honiara	*	*	*
		Suva	*	*	*
		Port Vila			
	Sub-module 2	Auckland	*	*	*
		Brisbane	*	*	*
		Tauranga	*	*	*
		Noumea	*		*
	Sub-module 3	Lae	*	*	*
		Darwin			
	Sub-module 4	Jakarta	*	*	*
	Sub-module 5	Singapore	*	*	*
		Port Kelang	*	*	*
Module 7	Sub-module 1	Genoa	*	*	*
	Sub-module 2	Port Louis		*	
	Sub-module 3	Valencia	*	*	*
		Marsaxlokk	*	*	*

		Barcelona	*		*
	Sub-module 4	Mumbai	*		*
		Jebel Ali	*	*	*
		Jeddah	*	*	*

Note: For each studied module, we list the structural-core ports by sub-module and measure their individual topological centralities based on node centrality measures of degree, betweenness and closeness. An asterisk (*) indicates that on a given measure the respective port ranks at top 10% in the context of its own module. Note: Module 5, the smallest module that covers the geographical area mainly consisting of Greenland and Iceland, cannot be further divided into submodules.

Supplementary Table 4 Summary of data on the measurement of topological centrality of structural-core ports as a whole within individual modular communicties

	structure-core ports		1,000 random port sets							
	by node (%)	by link (%)	by node				by link			
			Mean (%)	SD (%)	Max (%)	Min (%)	Mean (%)	SD (%)	Max (%)	Min (%)
C 1	69.00*	13.67*	15.09	6.08	37.83	1.98	0.59	0.58	3.95	0.00
C 2	68.52*	13.37*	16.99	8.10	50.59	0.73	0.70	0.77	6.43	0.00
C 3	85.22*	15.39*	15.60	6.95	41.82	2.43	0.63	0.67	4.91	0.00
C 4	73.09*	11.77*	10.13	7.49	46.35	0.10	0.23	0.41	3.49	0.00
C 6	85.21*	31.58*	17.86	12.04	64.64	0.62	0.83	1.54	11.25	0.00
C 7	44.28*	4.58*	8.39	5.03	29.45	0.12	0.17	0.27	2.34	0.00

Note: The smallest modular community (C 5), which covers the geographical area mainly consisting of Greenland and Iceland, cannot be further divided into submodules. * indicates p-value < 0.001. Source data are provided as a Source Data file.

Supplementary Table 5 Pearson correlation coefficients between network indicators and port capacity, across datasets

Network Indicators	GLSN of 2015								GLSN of 2017							
	dataset (all routes)		sub-dataset (FC routes)		sub-dataset (International routes)		sub-dataset (FC International routes)		dataset (all routes)		sub-dataset (FC routes)		sub-dataset (International routes)		sub-dataset (FC International routes)	
	GLSN	C	GLSN	C	GLSN	C	GLSN	C	GLSN	C	GLSN	C	GLSN	C	GLSN	C
B	0.74	0.88	0.78	0.93	0.75	0.89	0.80	0.94	0.78	0.92	0.78	0.94	0.77	0.92	0.77	0.93
Z	0.47	0.57	0.49	0.59	0.51	0.58	0.53	0.55	0.53	0.63	0.54	0.65	0.56	0.64	0.57	0.67
P	0.39	0.46	0.44	0.57	0.38	0.47	0.44	0.57	0.43	0.54	0.45	0.58	0.42	0.56	0.44	0.61
K	0.77	0.87	0.82	0.88	0.78	0.86	0.83	0.88	0.81	0.87	0.83	0.89	0.82	0.87	0.84	0.89
BC	0.68	0.82	0.77	0.80	0.67	0.82	0.76	0.75	0.75	0.83	0.80	0.84	0.74	0.82	0.79	0.80
φ	0.62	0.80	0.69	0.83	0.62	0.79	0.69	0.81	0.66	0.79	0.67	0.82	0.66	0.79	0.67	0.82
ρ_{C}	0.26	0.55	0.31	0.53	0.26	0.53	0.30	0.46	0.29	0.44	0.30	0.51	0.29	0.43	0.29	0.50
ρ_{CM}	0.58	0.79	0.66	0.82	0.60	0.79	0.65	0.80	0.63	0.77	0.63	0.80	0.61	0.76	0.62	0.80

Note: Network indicators *B*, *Z, P, K,* and *BC* denote gateway-ness, provincial-ness, connector-ness, degree and betweenness centrality, respectively; φ, the unnormalized rich-club coefficient originally proposed in the reference[4]; ρ_{C} and ρ_{CM}, two normalized versions of the rich-club coefficient that are proposed in reference[5] and in reference[6], respectively. The Pearson correlation coefficient between each network indicator and port capacity is calculated for all ports in the GLSN, and also separately for ports in individual communities (abbreviated to C); for the latter, the result is averaged over all communities in the GLSN. For each data point, we report the mean Pearson correlation coefficient over 1,000 repetitions of the Louvain-algorithm-based community division experiment. Standard errors for all data points are smaller than 0.002. Source data are provided as a Source Data file.

Supplementary Table 6 Pearson correlation coefficients between network indicators and port capacity in the modified GLSNs of 2015, across removal methods and percent of removed routes

Network Indicators	Increasing removal										Random removal									
	10% routes		20% routes		30% routes		40% routes		50% routes		10% routes		20% routes		30% routes		40% routes		50% routes	
	GLSN	C	GLSN	C	GLSN	C	GLSN	C	GLSN	C	GLSN	C	GLSN	C	GLSN	C	GLSN	C	GLSN	C
B	0.75	0.90	0.76	0.89	0.76	0.89	0.77	0.88	0.80	0.91	0.74	0.89	0.75	0.89	0.75	0.89	0.75	0.89	0.74	0.89
Z	0.48	0.58	0.47	0.57	0.47	0.58	0.47	0.56	0.49	0.59	0.48	0.57	0.48	0.57	0.49	0.57	0.49	0.57	0.50	0.57
P	0.39	0.48	0.39	0.47	0.40	0.48	0.40	0.45	0.41	0.47	0.39	0.48	0.39	0.50	0.40	0.51	0.40	0.53	0.41	0.54
K	0.78	0.87	0.78	0.86	0.79	0.87	0.80	0.89	0.81	0.88	0.77	0.86	0.78	0.86	0.79	0.86	0.79	0.86	0.80	0.85
BC	0.69	0.82	0.71	0.82	0.71	0.82	0.72	0.84	0.77	0.86	0.68	0.81	0.69	0.81	0.70	0.80	0.70	0.80	0.71	0.80
φ	0.63	0.80	0.65	0.79	0.65	0.80	0.66	0.82	0.66	0.78	0.63	0.80	0.64	0.81	0.66	0.81	0.67	0.81	0.68	0.81
ρ_{C}	0.26	0.49	0.29	0.46	0.28	0.47	0.28	0.51	0.29	0.41	0.27	0.55	0.29	0.55	0.31	0.56	0.32	0.57	0.34	0.58
ρ_{CM}	0.58	0.78	0.61	0.77	0.60	0.77	0.60	0.79	0.61	0.75	0.58	0.78	0.59	0.78	0.61	0.78	0.63	0.79	0.64	0.79

Note: Network indicators *B*, *Z*, *P*, *K*, and *BC* denote gateway-ness, provincial-ness, connector-ness, degree and betweenness centrality, respectively; φ, the unnormalized rich-club coefficient originally proposed in the reference[4]; ρ_{C} and ρ_{CM}, two normalized versions of the rich-club coefficient that are proposed in reference[5] and in reference[6], respectively. The Pearson correlation coefficient between each network indicator and port capacity is calculated for all ports in the GLSN, and also separately for ports in individual communities (abbreviated to C); for the latter, the result is averaged over all communities in the GLSN. For each data point, we report the average Pearson correlation coefficient over 1,000 repetitions of the experiment. Specifically, for results regarding increasing removal of shipping routes with smallest traffic capacity, we repeated 1,000 times the Louvain algorithm for modular community division; for results regarding uniform random removal of shipping routes, the uniform random selection of shipping routes to be dropped was sampled 1,000 times. Standard errors for all data points are smaller than 0.003. Source data are provided as a Source Data file.

Supplementary Table 7 Pearson correlation coefficients between network indicators and port capacity in the modified GLSNs of 2017, across removal methods and percent of removed routes

Network Indicators	Increasing removal										Random removal									
	10% routes		20% routes		30% routes		40% routes		50% routes		10% routes		20% routes		30% routes		40% routes		50% routes	
	GLSN	C	GLSN	C	GLSN	C	GLSN	C	GLSN	C	GLSN	C	GLSN	C	GLSN	C	GLSN	C	GLSN	C
B	0.78	0.92	0.78	0.93	0.78	0.93	0.79	0.85	0.78	0.86	0.78	0.91	0.78	0.91	0.78	0.91	0.78	0.90	0.77	0.90
Z	0.53	0.63	0.54	0.62	0.54	0.62	0.55	0.58	0.56	0.57	0.54	0.63	0.54	0.62	0.55	0.61	0.55	0.61	0.56	0.60
P	0.42	0.52	0.43	0.53	0.42	0.53	0.41	0.49	0.41	0.50	0.43	0.54	0.43	0.54	0.43	0.56	0.43	0.56	0.44	0.57
K	0.82	0.88	0.82	0.88	0.82	0.88	0.83	0.84	0.83	0.85	0.82	0.87	0.82	0.86	0.83	0.86	0.83	0.86	0.84	0.86
BC	0.74	0.83	0.74	0.83	0.73	0.83	0.76	0.81	0.77	0.84	0.76	0.82	0.77	0.82	0.77	0.82	0.78	0.82	0.79	0.81
φ	0.66	0.80	0.66	0.80	0.66	0.80	0.66	0.75	0.68	0.76	0.67	0.80	0.68	0.80	0.69	0.80	0.70	0.81	0.71	0.82
ρ_{C}	0.27	0.47	0.26	0.48	0.27	0.49	0.26	0.47	0.28	0.46	0.29	0.48	0.29	0.50	0.30	0.52	0.31	0.53	0.32	0.55
ρ_{CM}	0.61	0.78	0.62	0.78	0.61	0.78	0.59	0.73	0.62	0.72	0.61	0.75	0.62	0.76	0.63	0.77	0.65	0.78	0.67	0.80

Note: Network indicators *B*, *Z, P, K,* and *BC* denote gateway-ness, provincial-ness, connector-ness, degree and betweenness centrality, respectively; φ, the unnormalized rich-club coefficient originally proposed in the reference[4]; ρ_{C} and ρ_{CM}, two normalized versions of the rich-club coefficient that are proposed in reference[5] and in reference[6], respectively. The Pearson correlation coefficient between each network indicator and port capacity is calculated for all ports in the GLSN, and also separately for ports in individual communities (abbreviated as C); for the latter, the result is averaged over all communities in the GLSN. For each data point, we report the average Pearson correlation coefficient over 1,000 repetitions of the experiment. Specifically, for results regarding increasing removal of shipping routes with smallest traffic capacity, we repeated 1,000 times the Louvain algorithm for modular community division; for results regarding uniform random removal of shipping routes, the uniform random selection of shipping routes to be dropped was sampled 1,000 times. Standard errors for all data points are smaller than 0.003. Source data are provided as a Source Data file.

Supplementary Table 8 Pearson correlation coefficients between network indicators and port capacity (across modification methods and percent of edges), based on 2015 dataset

Network Indicators	Method #1									
	10%		20%		30%		40%		50%	
	GLSN	C	GLSN	C	GLSN	C	GLSN	C	GLSN	C
B	0.73	0.87	0.73	0.86	0.72	0.86	0.72	0.85	0.71	0.85
Z	0.47	0.56	0.47	0.56	0.47	0.56	0.47	0.56	0.47	0.56
P	0.29	0.33	0.22	0.25	0.17	0.18	0.13	0.13	0.10	0.09
K	0.77	0.85	0.77	0.84	0.77	0.83	0.77	0.83	0.77	0.83
BC	0.70	0.83	0.71	0.83	0.72	0.82	0.72	0.82	0.73	0.81
φ	0.62	0.79	0.62	0.79	0.62	0.78	0.62	0.78	0.62	0.77
ρ_{C}	0.26	0.53	0.26	0.52	0.26	0.51	0.26	0.51	0.27	0.51
ρ_{CM}	0.58	0.77	0.57	0.77	0.57	0.76	0.57	0.76	0.57	0.75

Network Indicators	Method #2									
	10%		20%		30%		40%		50%	
	GLSN	C	GLSN	C	GLSN	C	GLSN	C	GLSN	C
B	0.76	0.82	0.76	0.82	0.76	0.82	0.76	0.82	0.76	0.82
Z	0.71	0.77	0.71	0.77	0.71	0.77	0.71	0.77	0.71	0.77
P	0.15	0.19	0.15	0.19	0.15	0.19	0.15	0.19	0.15	0.19
K	0.77	0.82	0.77	0.82	0.77	0.82	0.77	0.82	0.77	0.82
BC	0.83	0.88	0.83	0.88	0.83	0.88	0.83	0.88	0.83	0.88
φ	0.65	0.73	0.65	0.74	0.65	0.74	0.65	0.73	0.65	0.74
ρ_{C}	-0.33	-0.39	-0.30	-0.37	-0.30	-0.38	-0.31	-0.39	-0.31	-0.38
ρ_{CM}	0.57	0.66	0.58	0.67	0.58	0.67	0.58	0.67	0.59	0.68

Network Indicators	Method #3									
	10%		20%		30%		40%		50%	
	GLSN	C	GLSN	C	GLSN	C	GLSN	C	GLSN	C
B	0.74	0.89	0.74	0.89	0.74	0.88	0.75	0.87	0.76	0.86
Z	0.47	0.55	0.48	0.54	0.50	0.54	0.52	0.55	0.56	0.58
P	036	0.44	0.33	0.40	0.30	0.37	0.26	0.33	0.23	0.29
K	0.77	0.86	0.77	0.86	0.77	0.86	0.77	0.85	0.77	0.84
BC	0.74	0.85	0.77	0.86	0.79	0.87	0.80	0.88	0.82	0.88
φ	0.59	0.78	0.59	0.77	0.60	0.76	0.61	0.75	0.61	0.74
ρ_{C}	-0.06	0.28	-0.33	-0.03	-0.54	-0.35	-0.61	-0.55	-0.61	-0.68
ρ_{CM}	0.45	0.67	0.40	0.62	0.31	0.56	0.27	0.50	0.20	0.41

Note: Network indicators *B*, *Z, P, K,* and *BC* denote gateway-ness, provincial-ness, connector-ness, degree and betweenness centrality, respectively; φ, the unnormalized rich-club coefficient originally proposed in the reference[4]; ρ_{C} and ρ_{CM}, two normalized versions of the rich-club coefficient that are proposed in reference[5] and in reference[6], respectively. The Pearson correlation coefficient between each network indicator and port capacity is calculated for all ports

in the GLSN, and also separately for ports in individual communities (abbreviated to C); for the latter, the result is averaged over all communities in the GLSN. For method #1, we uniformly randomly added into the original GLSN between 10%, 20%, 30%, 40%, and 50% of the amount of edges in this network. For method #2 and method #3, we randomly rewired between 10%, 20%, 30%, 40%, and 50% of the edges in the original GLSN based on two edge-rewiring processes proposed in reference[7] and reference[6], respectively. For each data point, we report the average Pearson correlation coefficient over 1,000 repetitions of the corresponding experiments of modification. Standard errors for all data points are smaller than 0.012. Source data are provided as a Source Data file.

Supplementary Table 9 Pearson correlation coefficients between network indicators and port capacity (across modification methods and percent of edges), based on 2017 dataset

Network Indicators	Method #1									
	10%		20%		30%		40%		50%	
	GLSN	C	GLSN	C	GLSN	C	GLSN	C	GLSN	C
B	0.77	0.90	0.76	0.89	0.76	0.88	0.75	0.87	0.74	0.87
Z	0.53	0.62	0.54	0.62	0.54	0.62	0.53	0.61	0.53	0.61
P	0.30	0.37	0.22	0.27	0.17	0.19	0.12	0.14	0.08	0.09
K	0.81	0.86	0.81	0.85	0.81	0.85	0.81	0.84	0.81	0.84
BC	0.76	0.83	0.77	0.83	0.78	0.83	0.79	0.83	0.80	0.82
φ	0.66	0.79	0.66	0.78	0.66	0.78	0.66	0.78	0.66	0.78
ρ_{C}	0.28	0.46	0.28	0.46	0.29	0.47	0.29	0.48	0.30	0.49
ρ_{CM}	0.61	0.74	0.61	0.75	0.61	0.75	0.61	0.75	0.61	0.75

Network Indicators	Method #2									
	10%		20%		30%		40%		50%	
	GLSN	C	GLSN	C	GLSN	C	GLSN	C	GLSN	C
B	0.80	0.86	0.80	0.86	0.80	0.86	0.80	0.86	0.80	0.86
Z	0.75	0.81	0.74	0.80	0.75	0.80	0.74	0.80	0.75	0.81
P	0.17	0.21	0.17	0.20	0.17	0.20	0.17	0.21	0.17	0.21
K	0.81	0.86	0.81	0.86	0.81	0.86	0.81	0.86	0.81	0.86
BC	0.90	0.92	0.90	0.92	0.90	0.92	0.90	0.92	0.90	0.92
φ	0.69	0.78	0.69	0.77	0.69	0.77	0.69	0.77	0.69	0.77
ρ_{C}	-0.31	-0.41	-0.34	-0.43	-0.32	-0.41	-0.33	-0.42	-0.33	-0.43
ρ_{CM}	0.61	0.72	0.60	0.71	0.60	0.72	0.61	0.72	0.61	0.72

Network Indicators	Method #3									
	10%		20%		30%		40%		50%	
	GLSN	C	GLSN	C	GLSN	C	GLSN	C	GLSN	C
B	0.78	0.92	0.78	0.91	0.78	0.91	0.78	0.90	0.79	0.89
Z	0.54	0.63	0.56	0.64	0.57	0.64	0.59	0.65	0.63	0.67

P	038	0.48	0.34	0.43	0.30	0.38	0.27	0.34	0.23	0.29
K	0.81	0.87	0.81	0.88	0.81	0.88	0.81	0.88	0.81	0.87
BC	0.80	0.85	0.83	0.88	0.85	0.90	0.87	0.91	0.88	0.91
φ	0.64	0.77	0.63	0.77	0.64	0.77	0.65	0.77	0.65	0.76
ρ_{C}	-0.07	0.14	-0.36	-0.17	−0.55	-0.49	-0.63	-0.70	-0.63	-0.74
ρ_{CM}	0.48	0.64	0.40	0.58	0.34	0.53	0.28	0.47	0.22	0.40

Note: Network indicators *B*, *Z, P, K,* and *BC* denote gateway-ness, provincial-ness, connector-ness, degree and betweenness centrality, respectively; φ, the unnormalized rich-club coefficient originally proposed in the reference[4]; ρ_{C} and ρ_{CM}, two normalized versions of the rich-club coefficient that are proposed in reference[5] and in reference[6], respectively. The Pearson correlation coefficient between each network indicator and port capacity is calculated for all ports in the GLSN, and also separately for ports in individual communities (abbreviated to C); for the latter, the result is averaged over all communities in the GLSN. For method #1, we uniformly randomly added into the original GLSN between 10%, 20%, 30%, 40%, and 50% of the amount of edges in this network. For method #2 and method #3, we randomly rewired between 10%, 20%, 30%, 40%, and 50% of the edges in the original GLSN based on two edge-rewiring processes proposed in reference[7] and reference[6], respectively. For each data point, we report the average Pearson correlation coefficient over 1,000 repetitions of the corresponding experiments of modification. Standard errors for all data points are smaller than 0.013. Source data are provided as a Source Data file.

Supplementary Table 10 Pearson correlation coefficients between topological indicators and port capacity in the modified GLSNs of 2015 and 2017

Network Indicators	modified GLSN of 2015		modified GLSN of 2017	
	GLSN	C	GLSN	C
B	0.58	0.74	0.63	0.75
Z	0.59	0.74	0.61	0.73
P	0.14	0.27	0.16	0.29
K	0.75	0.82	0.79	0.82
BC	0.63	0.70	0.71	0.73
φ	0.67	0.80	0.77	0.82
ρ_{C}	0.18	0.57	0.48	0.72
ρ_{CM}	0.63	0.77	0.80	0.81

Note: Network indicators *B*, *Z, P, K,* and *BC* denote gateway-ness, provincial-ness, connector-ness, degree and betweenness centrality, respectively; φ, the unnormalized rich-club coefficient originally proposed in the reference[4]; ρ_{C} and ρ_{CM}, two normalized versions of the rich-club coefficient that are proposed in reference[5] and in reference[6], respectively. The Pearson correlation coefficient between each network indicator and port capacity is calculated for all ports in the GLSN, and also separately for ports in individual communities (abbreviated to C); for the latter, the result is averaged over all communities in the GLSN. In the modified GLSNs edges are

restricted to link only consecutive ports along same shipping routes. For each data point, we report the average Pearson correlation coefficient over 1,000 repetitions of the Louvain-algorithm-based experiment of community division. Standard errors for all data points are smaller than 0.002. Source data are provided as a Source Data file.

Supplementary Notes

Supplementary note 1: Statistical significance of the economic small-world-ness of the GLSN

In this note, we demonstrate the statistical significance of the economic small-world-ness of the GLSN, by using both the real nautical distance between ports and the approximate great-circle distance between ports.

(1) Calculation based on the real nautical distance between ports

To validate the reliability of our results for the economic small-world properties of the GLSN that are obtained by the adoption of real nautical distance between ports—a high global efficiency of 0.827, a high local efficiency of 0.932 and a low cost of 0.015, we introduce a configuration null mode where links in the real network topology are randomly rewired with nodes' degree sequence being preserved[7]. Then, one-side *p*-values for the empirical results of global and local efficiency are computed as the respective percentage of permuted networks greater than or equal to the empirical case, and a one-side *p*-value for the empirical result of cost as the percentage of permuted networks smaller than or equal to the empirical case. By performing 1,000 permuted experiments, we get for the global efficiency a maximum of 0.543, a minimum of 0.499, and a mean of 0.517 (standard deviation = 0.006); for the local efficiency, a maximum of 0.459, a minimum of 0.400, and a mean of 0.433 (standard deviation = 0.009); and for the cost, a maximum of 0.035, a minimum of 0.034, and a mean of 0.035 (standard deviation = 0.000). Thus, we get for the global efficiency, local efficiency and cost of the empirical structure the corresponding *p*-values smaller than 0.001, suggesting the architecture of the GLSN is significantly an economic small world.

(2) Calculation based on the great-circle distance between ports

We noticed that if the physical length of an inter-port connection was approximated as the great-circle distance based on ports' geographical locations of latitude and longitude, the reported results for the economic small-world properties of the GLSN remain almost invariant. Details are as follows.

To validate the reliability of our results for the economic small-world properties of the GLSN that are obtained by the adoption of great-circle distance between ports—a high global efficiency of 0.818, a high local efficiency of 0.934 and a low cost of 0.016, we introduce a configuration null mode where links in the real network topology are randomly rewired with nodes' degree sequence being preserved[7]. Then, one-side *p*-values for the empirical results of global and local efficiency are computed as the respective percentage of permuted networks greater than or equal to the empirical case, and a one-side *p*-value for the empirical result of cost as the percentage of permuted networks smaller than or equal to the empirical case. By performing 1,000 permuted experiments, we get for the global efficiency a maximum of 0.521, a minimum of 0.485, and a mean of 0.502 (standard deviation = 0.006); for the local efficiency, a maximum of 0.467, a minimum of 0.408, and a mean of 0.440 (standard deviation = 0.010); and for the cost, a maximum of 0.035, a minimum of 0.034, and a mean of 0.034 (standard deviation = 0.000). Thus, we get for

the global efficiency, local efficiency and cost of the empirical structure the corresponding *p*-values smaller than 0.001, suggesting the architecture of the GLSN is significantly an economic small world.

Supplementary note 2: More discussions about the GLSN's basic topological properties and economic small-world-ness

This study follows the meaningful attempts of previous studies[11,12] to quantitatively explore the influence of containerized shipping on international trade flows; for instance, reference[11] adopted a gravity model to analyse the influence of shipping distance and frequency of shipping services on containerized trade flows between countries. This study for the first time investigates how the topological structure of a modern GLSN is associated to international trade. To this end, we first performed a careful analysis of its basic topological properties and economic small-world-ness properties, as reported in the main article. This note provides more discussions about these properties of the GLSN.

(1) Degree centrality distribution and assortativity

Degree (i.e. the number of connections that a node possesses in the network) is the most fundamental network measure to which most other connectivity measures are ultimately linked, and thus investigating degree distribution is generally a first step to detect whether a network is structurally heterogeneous. We report the cumulative probability distribution of port degree is well fitted by an exponential function (Fig. 2a in the main article), instead of power-law (tests on the power-law distribution of the data failed based on the method of Clauset et al[1] and the method of Voitalov et al[13] as well). Our result suggests the non-scale-free behavior of port connections in maritime shipping practices and is consistent with the finding of previous work of Deng et al (2009)[14] and Kaluza et al (2010)[15]. Another study that was based on cargo ships' trajectories data reported that ports' degree distribution was best fitted by a lognormal function in early years (around 1890-1960) and by a Weibull distribution in later years (around 1960-2008)[16]. Indeed, unlike many real-word networks where the vertex connectivities follow a scale-free power-law distribution[17], spatial networks where nodes are embedded into real space usually face certain spatial constraints restricting the appearance of so large degree nodes that would facilitate scale-free behaviors of node degree distribution[18]. While not scale-free, node degrees for single ports can vary over a great significant range of two-digit magnitude. Specifically, ports of Rotterdam and Antwerp have the highest degree of 324, while a lot of small ports are of the lowest degree of 1. Indeed, world liner shipping activities concentrate heavily on a few big ports; for instance, the top 30 ports account for over half of the total global container throughput in the year 2015. Most of those big ports are intermediate hubs, whose emergence was mainly fostered by world liner shipping companies in the pursuit of economies of scale (Rodrigue and Notteboom, 2010)[19]. Since the mid-1990s, the world has seen a fast increase of vessel size, frequency of liner shipping companies' services, and the concentration of their services at a few large intermediate hubs (Zohil and Prijon, 1999)[20].

In various kinds of real-life networks nodes with high degrees may connect randomly or they may connect preferentially to one another. We examined port connection patterns of the GLSN by computing the network's assortativity[21], finding the GLSN exhibits neutral assortativity (-0.024) for port degrees. The GLSN is by no means a random network as shipping companies are very meticulous about designing their service networks for profit's sake. Such neutral assortativity for port degrees is actually caused by the degree-match complexity (i.e., the coexistence of both negative and positive patterns), which can be understood if one considers the hub-and-spoke network configuration[3] widely adopted by liner shipping companies in the service network design. In regional port systems hub ports tend to be connected with many small ports in their regions[22], in order to support high-efficiency cargo distribution at regional levels. On the other hand, seeking to serve the inter-regional commodity transportation with a relatively low cost, shipping companies usually set up inter-regional liner services among a few hub ports in individual regions, fostering preferential connections among hub ports in the GLSN. Assortativity analysis of the GLSN may be even more valuable if it can be combined with the information on feeder and shortsea service routes in the GLSN. Nevertheless, neutral assortativity of node degree is an important topological characteristic for many spatial networks, and one plausible mechanism for such phenomenon, as has been suggested by some existing research[18,23], is spatial constraints. Because there is a cost associated to the length of links, the tendency for other nodes to connect to hubs is limited by the need to use the links.

(2) Betweenness and closeness centrality

As can be seen from the complementary cumulative probability distribution of betweenness centrality for ports (Fig. 2b in the main article), in the GLSN only a few ports are with significantly larger betweenness centralities. The betweenness distribution presents a power-law tail that is with an exponent 1.171 and corresponds to ports with $BC \geq 0.0043$, according to the statistical test of Clauset et al[1]. In an equivalent random network (i.e., a random graph with the same degree distribution as the real network), the distribution of betweennesses would still decay as a power law with a slightly larger exponent value, 1.208 (mean across 80.4% (804 out of 1,000) iterations passing the test), but the top five largest betweenness values would be much smaller than the counterparts in the real GLSN. A comparison of the two cases shows the existence of some excessively large betweenness values in the GLSN. Central ports of the GLSN mainly concentrate in Europe (e.g., ports of Rotterdam, Antwerp, and Hamburg), Asia (e.g., ports of Singapore, Shanghai, and Hong Kong) and North America (e.g., port of New York-New Jersey and port of Houston). As Asia - Europe, Asia - North America, and Europe - North America are the three main inter-regional commodity flows in the world, maritime transport linking these three continents—known as the East-West trunk line—dominates world's inter-regional shipping activities. Therefore, ports located along this trunk line are more likely to develop into transshipment hubs and thus to have relatively large betweenness centralities. In the context of the hub-and-spoke service network design of liner shipping companies, trunk line services are those linking hub ports of different geographical

regions. Such hub-and-spoke routing strategies are also commonly adopted in airlines' service network configuration. And the betweenness centrality distribution of the global air transport network[24] and that of the Chinese domestic air transport network[25] had also been reported to present a power-law decay.

It is quite surprising to find some central ports are not highly connected in the GLSN. For instance, port of Reykjavik from Iceland ranks world's 31th by betweenness centrality but only ranks world's 329th by degree, and port of Surabaya from Indonesia ranks the 28th and 127th by betweenness centrality and degree, respectively. Such phenomenon of degree-betweenness anomalies has been reported in the global air transportation network[24]. In their work, air ports with anomalous centralities (i.e. large betweenness centralities but small degrees) were found to be related with the existence of regions with a high density of airports but a few connections to the outside, and thus to be ultimately related to the community structure of the network. This is also the case of the GLSN, even though the degree-betweenness anomalies in the GLSN may not be as critical as in the global air transportation network.

Most ports in the world, though unevenly developed in port technology, have quite good reachability in the GLSN measured by closeness centrality. Closeness centrality is defined as the inverse of the average shortest path length from one node to all other nodes in the network[26]. As shown in Fig. 2c in the main article, more than 80 percent ports are of closeness centrality larger than 0.333, indicating that cargo transportation between these ports and others can be realized with transshipment no more than twice, on average. The average closeness centrality of word ports in the GLSN is 0.382. In an equivalent random graph, the average closeness centrality is estimated to be 0.440 (mean value across 1,000 iterations). A comparison of closeness centrality values between the empirical and random cases shows that ports' accessibility in the GLSN is close to what would be expected to appear at random: in both cases cargo can be transported to any port in the world from any given port by transshipment within twice, on average. Hence, it would be natural to expect the GLSN as a whole is self-organized into a small-world structure.

(3) Local community paradigm organization

We measured the LCP-corr of the considered GLSN and it is 0.97. This is a confirmation that the GLSN displays a local community paradigm organization, therefore it shares a similar structure with dynamic and heterogeneous networks such as the ones derived from social and biological systems. The LCP-corr of the GLSN is very high, like airport networks[27]. Airport networks, different from merely geographically constrained networks such as road-maps, have high LCP-corr because significant socio-economic and political factors also contribute to shape their topology[27]. Therefore, this result might imply that the GLSN topology is influenced significantly also by socio-economic factors, rather than by geographical constrains alone.

(4) Small-world-ness

As we report in the main article, the GLSN is a small-world network judged by its average

shortest path length and average clustering coefficient. We note that for transportation networks based on a series of transport routes (e.g., subway networks and liner shipping networks), the space-P representation of network topology (where any two nodes in a route is assigned with an edge) will naturally give an average shortest path length shorter than and an average clustering coefficient larger than their respective counterparts given by the space-L representation of network topology (where only consecutive nodes in a route is assigned with an edge). Previous studies[3,22] had compared some basic topological properties of global maritime shipping networks observed under the two representations of network topology.

(5) Economic small-world-ness

We noticed that if the physical length of an inter-port connection was approximated as the great-circle distance based on ports' geographical locations of latitude and longitude, the reported results for the economic properties of the GLSN remain almost invariant (Supplementary Note 1).

The finding that GLSN structure displays an economical organization is very important, because in so many real cases of man-made system design we wish to build up a system with excellent structure that is able to support high efficiency in flow transfer at both global and local levels but takes a low cost for construction; think, for instance, of infrastructure systems such as power grid networks, of urban public transportation systems like subway networks and bus networks, and of service networks of transportation companies like airlines and shipping carriers. Given the hub-and-spoke strategies adopted by individual shipping carriers in the service network design, it would be a plausible conjecture that the economical behavior of the GLSN is related with the existence of a certain kind of hub ports, which may form a cohesive core facilitating the integration of individual segregated regions in the world and thus ensuring high efficiency of cargo transportation across the entire network with a relatively low cost. Therefore, to seek a comprehensive understanding of how the GLSN is structurally organized, our finding of the economic small-world-ness of the GLSN points to the need to reveal port communities in the GLSN and define individual ports' roles based on their patterns of intra-community and inter-community connections, and further, to test the existence of a cohesive core and its relevance to the GLSN's structurally integration and functional outcomes in realizing the cargo transportation of international trade.

Supplementary note 3: A brief discussion of long-distance connections across port communities in the GLSN

Indeed, in systems where nodes are embedded into physical space there is a cost associated with each link, which inevitably results in the existence of spatial constraints in the formation of long-range links[18]. Therefore, spatial systems, ranging from transportation networks[28] to brain networks[29,30], generally present features of economical trade-off between network cost and efficiency: they tend to minimize connection cost and maximize the value that topological efficiency adds to the integrative processing capacity of the whole system. In the GLSN, these long-

distance connections across port communities, though costly, considerably reduce the path-length for cargo transportation between ports in different communities, facilitating efficient cargo movement due to less transshipment.

Supplementary note 4: A discussion of the rationale behind the structural core definition

The GLSN seems to be formed by ports with network-specific roles—provincial hubs, gateway hubs and connector hubs. The relevant question is, however, how the presence of these specific roles affects the performance of the GLSN? Particularly, given the structural segregation of the GLSN as is presented by the multiscale modular structure, we are interested in investigating whether some ports with specific hub roles form a structural core and thus, as a cohesive whole, facilitate structural integration of the GLSN. Intuitively, such a network core shall be a set of ports that are highly and mutually inter-connected. Our heuristic assumption is also that, for a complex network with a modular structure, a structural core (if exists) should be expected to facilitate the integration of all the individually segregated network parts (i.e. modular communities). In this regard, we would naturally expect a valid structural core consists of core nodes covering all individual modular communities. This is particularly true in the case of the GLSN, because there is not any individual region in the world that could be self-sufficient; instead, world regions (here divided into port communities) trade with others and are integrated into the global maritime transportation system.

Supplementary note 5: Robustness of empirical findings on the structural-core organization of the GLSN to the non-detrimental property of the Louvain algorithm in community division

As written in the main text, a structural core of the GLSN is defined as a set of hub ports that meet the following two criteria: first, this set should consist of the largest number of the most important hub ports that form a subgraph of high density (i.e. the proportion of actual links in the maximum possible number of links; in the presented study it is considеted to be at least 0.8); second, this set should contain at least one hub port from each individual modular community in the network. By definition, the structural-core organization of the GLSN is related with the modular community structure of this network. Thus the detection of a structural core of the GLSN is related with the modular community partition of this network that is obtained by the Louvain algorithm. The Louvain algorithm is a greedy algorithm that attempts to maximize the modularity of a partition of the network[8]. Although modularity maximization is hard[31] and the Louvain algorithm might miss some small structures in optimizing a partition for the modularity maximumization, at large scale this algorithm can be trusted[32].

Now we show that our findings on the structural-core orgainization of the GLSN are robust to the non-detrimental property of the Louvain algorithm in community division. Specifically, we ran the louvain algothrim for 1,000 times in the GLSN of 2015 and obtained 1,000 community partitions, then replicated the findings on the structural-core organization of the GLSN. Indeed, the

modularity values are quite stable across 1,000 runs: maximum is 0.476; minimum, 0.452; mean, 0.471 (standard error is 0.0001).

First of all, the detection of the gateway-hub structural core is insensitive to the variance of Louvain algorithm performance across 1,000 runs. As Supplementary Fig. 8 shows, in 921 runs a structural core was successefully detected based on the gateway-ness (*B*), meaning a probability of 92.1% with which one can successfully detect a gateway-hub structural core of the GLSN. By contrast, such probabilities of detecting a structrual core would be almost 0%, if one uses other topological indicators including provicial-ness (*Z*), connector-ness (*P*), degree (*K*), or betweenness centrality (*BC*). Even in very rare cases a structural core can be detected by *Z*, *P*, *K*, or *BC*, it can anyway be detected by *B*. Remind that in the main text we considered three types of hub ports: provincial hubs (i.e. ports with $Z \geq 1.5$), gateway hubs (i.e. ports with $B \geq 1.5$), and connector hubs (i.e. ports with $P \geq 1.5$). Now we also consider two conventional types of hub ports: large-degree hubs (i.e. ports with *K* at least 1.5 standard deviations above the mean of all ports in the network), and large-betweenness hubs (i.e. ports with *BC* at least 1.5 standard deviations above the mean of all ports in the network).

Second, the strong association of gateway-ness to port capacity holds well, within both the context of the GLSN and the context of individual communities. Supplementary Table 2 shows the mean values of Pearson correlation coefficients between respecitve topological indicators and port capacity, over 1,000 runs. Notably, gatway-ness (*B*) is more highly correlated with port capacity than provincial-ness (*Z*) and connector-ness (*P*) are. In the context of individual communities, the Pearson correlation coefficient between gatway-ness and port capacity even reachs a level as high as the Pearson correlation coefficient between degree and port capacity.

Last but not least, the strong association between the structural-core ports and international trade is well maintained. As Supplementary Fig. 9 shows, the Pearson correlation coefficients between the GLSN topological indicators and international trade indicators of countries—calculated as mean values over 1,000 runs—remain at the same level as their counterparts calculated based on the selective community partition (which are reported in Fig. 9 in the main text). For each pairwise comparison, the variation of Pearson correlation coefficient is less than 0.1.

Supplementary note 6: Gateway-hub structural core organization of the GLSN at modular level

We report the GLSN is self-organized into a multiscale modular structure (i.e. submodules within modules), with the existence of gateway-hub structural core organization at both global and modular levels. Following the same logic of the analysis of the structural core organization of the entire network, here we present the results for the gateway-hub structural cores within the contexts of individual modules. Note that: one exceptional module that cannot be further divided into submodules is the smallest module, which covers the geographical area mainly consisting of Greenland and Iceland.

First, we identified the submodular-level gateway, provincial, and connector hubs in each

module (Supplementary Fig. 10), and then investigated for each type of hub ports if some most important ones formed a structural core organization. Under the definition of structural core proposed in the main text, we successfully filtered out for each studied module a set of submodular gateway-hub ports that form a structural core (Supplementary Fig. 11). But we did not detect any structural core based on either submodular provincial hubs or connector hubs. Specifically, we detected only in some modules the respective sets of most important hub ports that form sub-graphs of density at least 0.8 (Supplementary Fig. 12a and Fig. 13a), but these sets of hub ports do not involve all the individual submodules in their own modules (Supplementary Fig. 12b and Fig. 13b). And we further demonstrated that each module's detected structural core in the actual GLSN is statistically significant (p-value < 0.001), meaning that the probability to generate the same structural core at random is very low; see the detailed results presented in Supplementary Fig. 14. Specifically, as presented in Supplementary Fig. 14, each identified structural core forms a sub-network with a high density that is statistically very significant (p-value < 0.001, 1,000 testing against a configuration null model[7]). In other words, we evidenced for each identified set of submodular gateway-hub ports the high connectivity density among them is related with their connectivity patterns of being submodular gateway hubs but not simply because of their degree connectivities.

To further verify the gateway-hub structural core organization at modular level, we showed that the identified structural-core ports are topologically central—both individually and as a whole—in their respective modules, using the same method of validating the topological centrality of the structural-core ports of the entire network. The topological centrality of each individual core port is evaluated based on three basic node centrality indicators (i.e. degree, betweenness and closeness), and a port is considered topologically central if it ranks the top 10th percentile in its own module on a given indicator; see results in Supplementary Table 3. Topological centrality of the structural-core ports as a whole is measured by the percentage of shortest paths between any two non-core ports in the module that pass through the core by node and by link, respectively; see results in Supplementary Table 4, together with the results for statistical significance testing. Each result for the topological centrality of the related structural-core ports as a whole was shown to be statistically significant, in comparison to that of randomly selected 1,000 sets of ports corresponding in size to the number of ports constituting the structural core: we traced for each random set all shortest paths between nodes outside the set, getting the percentage of shortest paths travelling through the port set by node and by link, respectively; and then, computed a one-side p-value as the probability of the percentage of random sets greater than or equal to the empirical case. Respective p-values for all the empirical results turned out to be smaller than 0.001.

Additionally, a representation in the hyperbolic space—obtained by coalescent embedding[33]—of individual modular communities and their respective structural cores is offered in Supplementary Fig. 15. It reveals that those structural cores are indeed at the center not only of the network topology but also of the hidden network geometry. Of particular interest is module 3,

which is the largest community detected in the GLSN, consists of 213 ports, and mainly covers the geographical regions of East Asia and North American West Coast. The structural core detected in this module is located in the hyperbolic space not so centrally, and it seems that the structural-core ports are unequally distributed among the submodular communities. Indeed, the structural core is entirely composed of East Asian ports (as shown in the geographical plot), and the majority of them are world's leading container ports such as Chinese ports of Dalian, Hong Kong, Ningbo-Zhoushan, Qingdao, Shanghai, Shenzhen, and Tianjin, Korean port of Busan, and Japanese port of Tokyo. Such results suggest the dominance of East Asia in this port community, and it can be well understood if one considers the fact that world's largest seaborne trade market is the intra-Asia trade, particularly, East Asia.

Supplementary note 7: Algorithm for structural core detection and assessment of its significance

We provide here a description of the algorithm that we invented for structural core detection and assessment of its significance. In the present study, the modular community division is based on the Louvain algorithm[8]. The importance of each node can be measured by any given indicator, and we considered five topological indicators (i.e. Z, B, P, *K,* and BC) and the respective five types of hub ports that are defined by them: provincial hubs (i.e. ports with $Z \geq 1.5$), gateway hubs (i.e. ports with $B \geq 1.5$), connector hubs (i.e. ports with $P \geq 1.5$), large-degree hubs (i.e. ports with *K* at least 1.5 standard deviations above the mean of all ports in the network), and large-betweenness hubs (i.e. ports with *BC* at least 1.5 standard deviations above the mean of all ports in the network). We considered a density threshold of 0.8, and a configuration null model proposed in reference[7] that rewires links uniformly at random while keeping nodes' degrees in the real network.

If a structural core of a real network is detected, the algorithm returns the detected *SC* (i.e. a structural-core set, which means a set of structural-core nodes), α (i.e. density of the subgraph formed by the structural-core nodes and the links among them), and b (i.e. the smallest node-importance value among the structural-core nodes).

We evaluate the statistical significance of a detected structural core in the real GLSN, by accessing whether the probability to detect the same structural core in a null configuration of the GLSN is significantly low. Specifically, either of the following two different probabilities can be computed: *pval*_1, the probability that, in the null model, there exist a set of nodes corresponding to node-importance values $\geq b$ and the density of the subgraph formed by these nodes (denoted as β) $\geq \alpha$; *pval*_2, the probability that in the null model the density of the subgraph formed by the same structural-core nodes of the real GLSN (denoted as γ) $\geq \alpha$.

A. Pseudocode of the algorithm for structural core detection

INPUT: original network
OUTPUT: structural-core set, *SC;* the density of the subgraph formed by the nodes in *SC* and the links among them, α; the smallest node-importance value among the structural-core nodes, *b*.

Modular community division.
Measure the importance of each node by a given indicator.
if (there exists a set consisting of the largest number of the most important hub nodes that form a subgraph of density at least 0.8)
    if (this set contains at least one hub node from each modular community in the network)
        return *SC*, α, *b*
    else
        print('*SC* is not detected')
else
    print('*SC* is not detected')

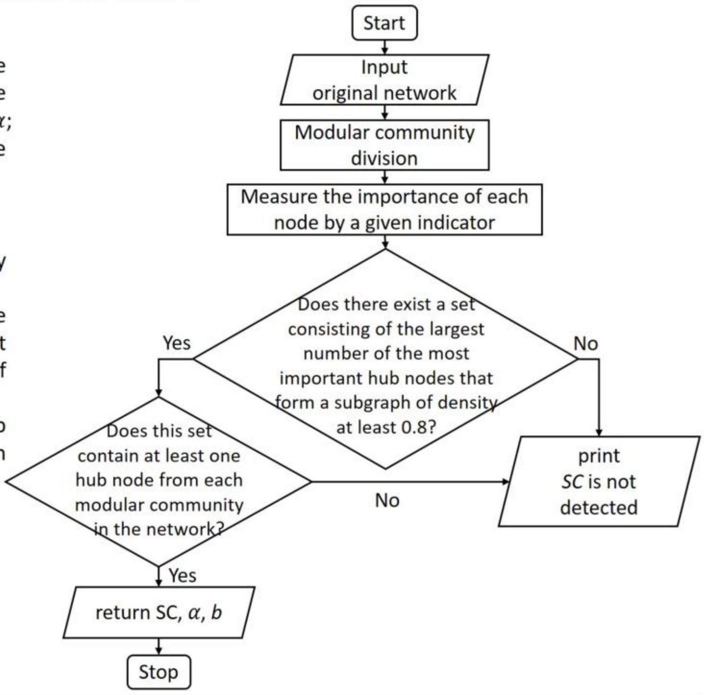

B. Pseudocode of the algorithm for assessing the statistical significance of a structural-core set, *pval*_1

INPUT: original network, a structural-core set *SC*, α, *b*, iterations *M*
OUTPUT: *pval*_1
DEFINE:
The density of the subgraph formed by the nodes with node-importance values $\geq b$ and the links among them in null model, β;
A p-value calculated as the probability of $\beta \geq \alpha$, *pval*_1.

$i = 0$;
if $i < M$
    generate a null model;
    calculate β_i;
    $i = i + 1$;
calculate *pval*_1.
print(*pval*_1)

Note: In this study we fixed *M* = 1000.

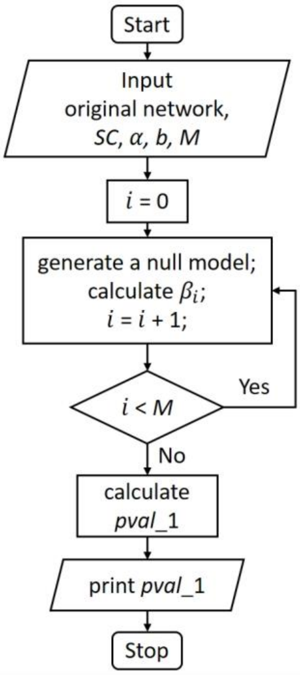

C. Pseudocode of the algorithm for assessing the statistical significance of a structural-core set, *pval*_2

INPUT: original network, a structural-core set *SC*, α, *b*, iterations *M*
OUTPUT: *pval*_2
DEFINE:
The density of the subgraph formed by the nodes in *SC* and the links among them in null model, γ;
A p-value calculated as the probability of $\gamma \geq \alpha$, *pval*_2.

```
i = 0;
if i < M
      generate a null model;
      calculate γ_i;
      i = i + 1;
calculate pval_2.
print(pval_2)
```

Note: In this study we fixed M = 1000.

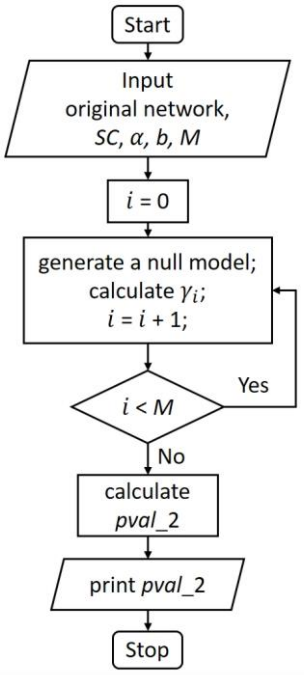

Supplementary note 8: Significant importance of core connections in supporting long-distance maritime transportation; calculations are based on great-circle distance

Here, we estimated the physical length of an inter-port connection as the great-circle distance based on ports' geographical locations of latitude and longitude, and then repeated the analysis. We found the results based on great-circle distance (which are reported in the following text) remain almost the same as those results based on real-nautical distance (which are reported in the main manuscript text).

As ports of the GLSN were divided into structural-core and non-structural-core ports, edges were naturally categorized into three topological types (Supplementary Fig. 16): core connections linking structural-core ports, feeder connections linking structural-core ports and non-structural-core ports, and local connections linking non-structural-core ports. Statistical analyses revealed the significant importance of core connections in supporting long-distance maritime transportation. First, core connections themselves tend to be longer than feeder and local connections (Supplementary Fig. 16b). Measured by great-circle distance (hereafter referred to as distance), the average length of core connections (average = 7,219 km, SD = 4,397 km) is 1.8 times of the average over all inter-port connections (average = 3,996 km, SD = 4,281 km); feeder connections (average= 5,910 km, SD = 4,730 km), 1.5 times; local connections (average = 2,857 km, SD = 3,539 km), 0.7 times.

When looking at the shipping distance for cargo transportation among all the non-core ports in the GLSN (Supplementary Fig. 16c), we found 13.5% of the total shipping distance—measured by the distance along the edges of their shortest paths—is taken up by core connections. As core connections only account for 3.2% of the total number of inter-port connections in the GLSN, it

makes a ratio of distance fraction to the connection fraction to be 4.2, relative to 1.8 and 0.4 for feeder connections and local connections, respectively. When considering those shortest paths that pass through the structural core (i.e. travelling across at least one core connection), the proportion of shipping distance taken up by core connections reaches 53.6%, and that by feeder and local connections 40.8% and 5.6%, respectively.

Supplementary note 9: Robustness of the structural-core organization of the GLSN across multiple datasets

Here we used the following new datasets and replicated the key findings on the structural-core organization of the GLSN—the existence of a (gateway-hub) structural core, the strong association between gateway-ness and port capacity, and the strong association between the structural core and international trade. Specifically, we now have used the dataset of world liner shipping service routes in the year 2017 as well as the previous one in the year 2015. In addition, we derived from each dataset 3 sub-datasets that consist of three different types of liner shipping service routes: full-container routes (i.e., routes deployed with full-container vessels; denoted as FC routes), international routes (i.e., routes including ports from at least two countries), full-container international routes (i.e., routes that are deployed with full-container vessels and include ports from at least two countries; denoted as FC International routes). Note that in practice liner shipping service routes can be categorized into different types by shipping companies, according to the type of vessels deployed and the number of countries involved. The dataset of GLSN 2015 contains 1622 liner shipping service routes, including 1449 full-container routes, 1472 international routes, and 1316 full-container international routes. The dataset of GLSN 2017 contains 1604 liner shipping service routes, including 1518 full-container routes, 1469 international routes, and 1393 full-container international routes. Detailed results are as follows.

First of all, the detection of a gateway-hub structural core proves robust across multiple datasets. For all datasets and sub-datasets, one can detect a structural core of the GLSN based on the topological indicator gateway-ness with a probability close to 100% (Supplementary Fig. 17). The probability of detection was estimated based on 1,000 iterations of the Louvain algorithm; for detailed analyses regarding the robustness of empirical findings on the structural-core organization of the GLSN to the non-detrimental property of the Louvain algorithm in community division, see Supplementary Note 5. Specifically, we performed 1,000 times the Louvain algorithm for modular community division, repeated the structural-core detection experiment in the obtained 1,000 community partitions, and counted the frequency with which a structural core was detected by using any given topological indicator.

Moreover, the strong association between gateway-ness and port capacity and strong association between the structural-core ports and international trade hold well, across datasets. For all datasets and sub-datasets, the Pearson correlation coefficients between gateway-ness and port capacity are at the same level as their respective counterparts based on the previous dataset

of world liner shipping service routes in 2015 (Supplementary Table 5). Likewise, the Pearson correlation coefficients between the GLSN topological indicators and international trade indicators of countries remain at the same level as those results based on the previous dataset of world liner shipping service routes in 2015 (Supplementary Fig. 18).

Supplementary note 10: Influence of the constraints on the structural-core organization of the GLSN

The GLSN, as an ensemble of world liner shipping companies' service networks, is essentially a complex spatial network constrained by the number of liner shipping routes, the physical geography of the earth, and the economy of liner shipping service network configuration. To evaluate the extent to which the structural-core organization of the GLSN was affected by these constraints, we repeated the analysis of structural-core detection in various modified networks where the constraints of the real GLSNs of 2015 and 2017 were modified in different ways and at different levels.

(1) Constraints of the number of shipping routes

There are many ports in the world which have just a few liner shipping routes. Most of them are either located at geographically remote areas or at countries poorly participating in international trade, while some are just feeder ports mainly connected to adjacent hub ports. Although these ports and the related shipping routes are negligible at the global level, they nonetheless are indispensable to the trade development of the regions they serve; they simply facilitate the integration of these regions into the wider global markets. To cover as many ports and geographical regions in the world as possible, we therefore initially constructed a GLSN using all liner shipping routes provided by the database.

Now, to evaluate the extent to which the structural-core organization of the GLSN is influenced by the constraint of the number of shipping routes, we have repeated the analysis in two types of modified GLSNs where we take control for the number of liner shipping service routes adopted. For the first type of modified networks, we increasingly removed between 10%, 20%, 30%, 40%, and 50% of shipping routes with smallest traffic capacity from the original datasets. For the second type of modified networks, in 1,000 iterations, we uniformly randomly removed between 10%, 20%, 30%, 40%, and 50% of shipping routes from the original datasets. The experiments were conducted in the GLSN datasets of 2015 and 2017.

Results show that the detection of a gateway-hub structural core was not subject to the number of shipping routes with small traffic capacity, while being sensitve to the number of shipping routes with large traffic capacity. As Supplementary Fig. 19 shows, when 10%, 20%, 30%, 40%, and 50% of shipping routes with smallest traffic capacity were removed, the probabilities with which one could detect a structural core by using gateway-ness (*B*) were estimated to be always close to 100% (yellows bars); probability estimation was based on 1,000 repeatitions of the Lovain

algorithm for modular community partition. For instance, for the GLSN of 2015, such probabilities were 98%, 99%, 99%, 93%, and 100%, respectively. However, when the same percent of shipping routes were uniformly randomly removed, such probabilities would obviously decrease (gray bars); probability estimation was based on 1,000 iterations of the uniform random removal of shipping routes. Nevertheless, they were still significantly greater than their counterparts based on provicial-ness (*Z*), connector-ness (*P*), degree (*K*), or betweenness centrality (*BC*). Note: in cases of uniform random removal, the topology of the GLSN was inevitably damaged due to loss of some shipping routes with large traffic capacity; in pracitce, such routes are generally trunk routes linking big ports in different geographical regions and thus are indispensable for the integration of the global liner shipping system.

Nevertheless, the strong association between gateway-ness and port capacity and the strong association between the structural-core ports and international trade hold well, across removal methods and percent of removed routes. In the modified GLSNs, the Pearson correlation coefficients between gateway-ness and port capacity remain at the same level as their counterparts in their respective original networks (Supplementary Tables 6 and 7); it is also the case for the Pearson correlation coefficients between the GLSN topological indicators and international trade indicators of countries (Supplementary Fig. 20).

(2) Geographical constraints

The GLSN is naturally constrained by the geography of the earth, that the longer an inter-port connection is, the more transportation cost it will need. Consequently, long-haul inter-port connections are much fewer than short-haul connections, and are available mainly for cargo transportation between a few hub ports of different geographical regions rather than between small ports of different regions. That is, due to such geographical constraints, liner shipping carriers tend to adopt the so-called hub-and-spoke service network configuration that allows them to consolidate cargo in a few hub ports of world regions and thus provide inter-regional transportation between regional hubs via larger vessels. The hub-and-spoke service network configuration proves effective in lowering the overall transport cost of a service network, pursuing the economies of scale[19].

Now, to evaluate the extent to which the structural-core organization of the GLSN is influenced by the geographical constraint, we now have repeated the analysis in three modified GLSNs where the geographical constraint of the real GLSNs of 2015 and 2017 is altered based on three different methods. For the first modified network, in 1,000 iterations, we uniformly randomly added into the original GLSN between 10%, 20%, 30%, 40%, and 50% of the amount of edges in an original GLSN. For the second and the third modified networks, in 1,000 iterations, we randomly rewired between 10%, 20%, 30%, 40%, and 50% of the edges in an original GLSN based on two different edge-rewiring processes. One was developed by Maslov and Sneppen[7] (abbreviated to MS null model), where two edges to swap are sampled with a uniform probability. The other was developed by Muscoloni and Cannistraci[6] (abbreviated to CM null model), where

the two probabilities of the two sampled swapping edges are defined separately in function of the adjacent nodes' degree and have an inverted tendency: one edge is sampled with a probability directly proportional to the product of the degrees of the adjacent nodes, whereas the other edge with a probability inversely proportional. With all these three methods, the geographical restriction of the real global liner shipping system was indeed altered, as can be seen from either or both of the following two phenomena. One is that long-haul connections would be much more common in modified networks than in the real GLSN; the larger amount of the rewired edges, the greater extent of the modification is (Supplementary Figs. 21(b) and 22(b)). Another is that in modified networks long-haul connections would involve many small ports (i.e., low degrees), whereas in the real GLSN they are quite restricted to big ports (Supplementary Figs. 21(a, c-e) and 22(a, c-e)).

It turned out that the existence of a gateway-hub structural core in such modified GLSNs was apparently much rarer—especially in the ones based on permutation methods of edge rewiring—than that in the original GLSN. Supplementary Fig. 23 presents for each modified GLSN the probabilities with which one could detect a structural core based on the gateway-ness and other topological indicators; in each case, such a detection probability for any given topological indicator was estimated based on 1,000 repetitions of the modification process. Moreover, in such modified networks the association between port capacity and gateway-ness would become less outstanding, relative to the association between port capacity and other topological indicators (Supplementary Tables 8 and 9). Similarly, regarding the association between the GLSN topological indicators and international trade indicators of countries, the performance of structural-core ports (if detected) would be less outstanding, relative to the performance of non-structural-core ports (Supplementary Figs. 24 and 25). To sum up, if the geographical constraints of the real GLSN were altered, a structural core of this network and its strong association with international trade would be less likely (if not impossible) to exist. These analyses demonstrate that the geographical constraints indeed play a crucial role in the formation of the structural-core organization of the GLSN.

(3) The constraints of the economy of liner shipping network

The hub-and-spoke network configuration has long been adopted by world liner shipping companies as well as by air lines, for maximizing the economy of their service networks[34,35]. With such a service network configuration, the economy of a liner shipping service network is obtained by the adoption of pre-fixed liner shipping service routes with multiple ports of call. That is, ships go back and forth among a series of ports along pre-fixed shipping routes, so that cargo between any two ports of a same route can be directly transported via a single ship with a relatively low cost. But the economy of an air transportation network is different; an air transportation route typically consists of only two end-point airports, and aircrafts simply fly back and forth between them. Such difference between liner shipping and air transportation is rooted in the different service aims of the two industries: the primary goal of an air company is to deliver air freights as

fast as possible, whereas the goal of a liner carrier is to achieve a good trade-off between operating cost and time efficiency. Hence, freight transportation by sea is much cheaper than by air. Considering that the multiple-ports-of-call property of liner shipping service routes is curial for the economy of liner shipping network, we constructed the original GLSN by assigning edges to any two ports of a same shipping route.

Now, to evaluate the extent to which the structural-core organization of the GLSN is influenced by the constraint of the economy of liner shipping network, we have repeated the analysis on the modified GLSNs of 2015 and 2017 where edges are restricted to link only consecutive ports along same shipping routes. In such modified GLSNs the economy of the original GLSNs is altered, in the sense that the multiple-ports-of-call property of liner shipping service routes is neglected.

Results show that in such a modified GLSN one completely cannot find a structural core (in 1,000 iterations) by using whichever of the five topological indicators, indicating that the economy of liner shipping network is an important factor which facilitates the structural-core organization of the GLSN. Specifically, we performed 1,000 times the Louvain algorithm for modular community division, repeated the structural-core detection experiment in the obtained 1,000 community partitions, and counted the frequency with which a structural core was detected. And this frequency turned out to be 0, for any of the five topological indicators. Moreover, the association of port capacity to gateway-ness is also weakened (Supplementary Table 10). Note that, when applied to such a modified GLSN, gateway-ness's power in capturing the gateway-hub role of a port in cargo transportation between its host region and the rest of the world in the real global liner shipping system would inevitably be damaged, due to the following reason. To a certain extent, the topology of the modified GLSN—which does not have edges linking any non-consecutive ports of a same shipping route—contains only partial information about the real global liner shipping system, since in reality there exist a considerable amount of cargo transportation between non-consecutive ports of a liner shipping service route via a single vessel.

Supplementary note 11: Existence of a structural core of the GLSN is not the same as small-world distance scaling

The existence of a structural core of the GLSN is not the same as the existence of small-world distance scaling. By definition, the gateway-ness and the associated gateway-hub structural core are dependent on the community structure of a network; to the best of our knowledge, the small-world-ness can be defined regardless of the community structure. To further clarify the difference between the structural-core organization and the small-world distance scaling, we now have conducted the following experiment. And we show that one cannot find a structural-core in networks simply with a small-world property, regardless of the level of small-world-ness.

We used an equivalent WS (Watts-Strogatz) model[9] of small world generation to generate a family of small-world networks, which keep the number of nodes and the average degree of the

real GLSN and as well as the small-world-ness property. Specifically, the WS model begins with a ring of n nodes (here n = 977, i.e. the number of nodes in the real GLSN), with each node being connected to its nearest neighbors out to some range K (here K = 34, i.e. the average degree of the real GLSN). Each edge in turn is re-wired to a new target node with probability p, ranging from 0 to 1 (Supplementary Fig. 26 (a)). Values of p = 0 and p = 1 give regular and random networks, respectively, with intermediate p values resulting in small-world networks.

Then, using the measure of small-world-ness (denoted as ω) developed by Telesford et al[10], we quantified the level of small-world-ness of the rewired network obtained in 1,000 iterations under each rewiring probability p, as well as the small-world-ness level of the real GLSN. The coefficient ω of a network is calculated based on the comparison of the characteristic path length L to L_{rand} of an equivalent random network (that preserves nodes' degrees of the network under study), and the comparison of the average clustering coefficient C to C_{latt} of an equivalent lattice network; for more details about ω, *see methods.* As measured by ω, the WS model indeed generated for the real GLSN a family of small-world networks that include not only ones with the same level of small-world-ness as the GLSN but also many others with different levels of small-world-ness (Supplementary Fig. 26 (b)).

Finally, we repeated the procedure of structural-core detection in the networks generated under each re-wiring probability p. We report that one cannot find a structural-core in any of these networks, regardless of the level of small-world-ness. Such analyses demonstrate that the existence of a structural core of the GLSN is not the same as the small-world distance scaling of the network.

Supplementary References

1. Clauset, A., Shalizi, C. R. & Newman, M. E. J. Power-law distributions in empirical data. *SIAM Rev.* **51**, 661–703 (2009).
2. Muscoloni, A. & Cannistraci, C. V. Angular separability of data clusters or network communities in geometrical space and its relevance to hyperbolic embedding. Preprint at https://arxiv.org/abs/1907.00025 (2019).
3. Hu, Y. & Zhu, D. Empirical analysis of the worldwide maritime transportation network. *Phys. A Stat. Mech. its Appl.* **388**, 2061–2071 (2009).
4. Zhou, S. & Mondragón, R. J. The rich-club phenomenon in the internet topology. *IEEE Commun. Lett.* **8**, 180–182 (2004).
5. Colizza, V., Flammini, A., Serrano, M. A. & Vespignani, A. Detecting rich-club ordering in complex networks. *Nat. Phys.* **2**, 110–115 (2006).
6. Muscoloni, A. & Cannistraci, C. V. Rich-clubness test: How to determine whether a complex network has or doesn't have a rich-club? Preprint at https://arxiv.org/abs/1704.03526 (2017).
7. Maslov, S. & Sneppen, K. Specificity and stability in topology of protein networks. *Science* **296**, 910–913 (2002).
8. Blondel, V. D., Guillaume, J.-L., Lambiotte, R. & Lefebvre, E. Fast unfolding of communities in large networks. *J. Stat. Mech. Theory Exp.* **2008**, P10008 (2008).
9. Watts, D. J. & Strogatz, S. H. Collective dynamics of 'small-world' networks. *Nature* **393**, 440–

442 (1998).

10. Telesford, Q. K., Joyce, K. E., Hayasaka, S., Burdette, J. H. & Laurienti, P. J. The ubiquity of small-world networks. *Brain Connect.* **1**, 367–375 (2011).
11. Guerrero, D., Grasland, C. & Ducruet, C. Explaining international trade flows with shipping-based distances. in *Maritime Networks: Spatial Structures and Time Dynamics* (ed. Ducruet, C.) 303–321 (Routledge, New York, NY, 2015).
12. Bernhofen, D. M., El-Sahli, Z. & Kneller, R. Estimating the effects of the container revolution on world trade. *J. Int. Econ.* **98**, 36–50 (2016).
13. Voitalov, I., van der Hoorn, P., van der Hofstad, R. & Krioukov, D. Scale-free networks well done. *Phys. Rev. Res.* **1**, 033034 (2019).
14. Deng, W.-B., Guo, L., Li, W. & Cai, X. Worldwide marine transportation network: efficiency and container throughput. *Chinese Phys. Lett.* **26**, 242–245 (2009).
15. Kaluza, P., Kölzsch, A., Gastner, M. T. & Blasius, B. The complex network of global cargo ship movements. *J. R. Soc. Interface* **7**, 1093–1103 (2010).
16. Gastner, M. T. & Ducruet, C. The distribution functions of vessel calls and port connectivity in the global cargo ship network. in *Maritime Networks: Spatial Structures and Time Dynamics* 242–261 (Routledge, New York, NY, 2015).
17. Barabási, A.-L. & Albert, R. Emergence of scaling in random networks. *Science* **286**, 509–512 (1999).
18. Barthélemy, M. Spatial networks. *Phys. Rep.* **499**, 1–101 (2011).
19. Rodrigue, J.-P. & Notteboom, T. Foreland-based regionalization: integrating intermediate hubs with port hinterlands. *Res. Transp. Econ.* **27**, 19–29 (2010).
20. Zohil, J. & Prijon, M. The MED rule: the interdependence of container throughput and transhipment volumes in the Mediterranean ports. *Marit. Policy Manag.* **26**, 175–193 (1999).
21. Newman, M. E. J. Assortative mixing in networks. *Phys. Rev. Lett.* **89**, 2087011–2087014 (2002).
22. Ducruet, C. & Notteboom, T. The worldwide maritime network of container shipping: spatial structure and regional dynamics. *Glob. Networks* **12**, 395–423 (2012).
23. Barrat, A., Barthélemy, M. & Vespignani, A. The effects of spatial constraints on the evolution of weighted complex networks. *J. Stat. Mech. Theory Exp.* **2005**, P05003 (2005).
24. Guimerà, R., Mossa, S., Turtschi, A. & Amaral, L. A. N. The worldwide air transportation network: anomalous centrality, community structure, and cities' global roles. *Proc. Natl. Acad. Sci. USA* **102**, 7794–7799 (2005).
25. Wang, J., Mo, H., Wang, F. & Jin, F. Exploring the network structure and nodal centrality of China's air transport network: a complex network approach. *J. Transp. Geogr.* **19**, 712–721 (2011).
26. Freeman, L. C. Centrality in social networks conceptual clarification. *Soc. Networks* **1**, 215–239 (1978).
27. Cannistraci, C. V., Alanis-Lobato, G. & Ravasi, T. From link-prediction in brain connectomes and protein interactomes to the local-community-paradigm in complex networks. *Sci. Rep.* **3**, 1613 (2013).
28. Latora, V. & Marchiori, M. Is the Boston subway a small-world network? *Phys. A Stat. Mech. its Appl.* **314**, 109–113 (2002).
29. Achard, S. & Bullmore, E. Efficiency and cost of economical brain functional networks. *PLoS*

Comput. Biol. **3**, 174–183 (2007).

30. Bullmore, E. T. & Sporns, O. The economy of brain network organization. *Nat. Rev. Neurosci.* **13**, 336–349 (2012).
31. Brandes, U. *et al.* On modularity clustering. *IEEE Trans. Knowl. Data Eng.* **20**, 172–188 (2008).
32. Fortunato, S. & Hric, D. Community detection in networks: a user guide. *Phys. Rep.* **659**, 1–44 (2016).
33. Muscoloni, A., Thomas, J. M., Ciucci, S., Bianconi, G. & Cannistraci, C. V. Machine learning meets complex networks via coalescent embedding in the hyperbolic space. *Nat. Commun.* **8**, 1615 (2017).
34. Robinson, R. Asian hub/feeder nets: the dynamics of restructuring. *Marit. Policy Manag.* **25**, 21–40 (1998).
35. O'Kelly, M. E. A geographer's analysis of hub-and-spoke networks. *J. Transp. Geogr.* **6**, 171–186 (1998).
