# Supplementary material for: Modular gateway-ness connectivity and structural core organization in maritime network science
Source: Nat Commun. 2020 Jun 5;11:2849. doi: 10.1038/s41467-020-16619-5 (PMC7275034; doi:10.1038/s41467-020-16619-5)
Supplement: Supplementary file 3 — Reporting Summary [file 41467_2020_16619_MOESM3_ESM.pdf]

nature research

Corresponding author(s): Mengqiao Xu, Haoxiang Xia, Carlo Vittorio Cannistraci

Last updated by author(s): April 15, 2020

Reporting Summary

Nature Research wishes to improve the reproducibility of the work that we publish. This form provides structure for consistency and transparency in reporting. For further information on Nature Research policies, see Authors & Referees and the Editorial Policy Checklist.

Statistics

For all statistical analyses, confirm that the following items are present in the figure legend, table legend, main text, or Methods section.

n/a	Confirmed	
☐	☒	The exact sample size (*n*) for each experimental group/condition, given as a discrete number and unit of measurement
☐	☒	A statement on whether measurements were taken from distinct samples or whether the same sample was measured repeatedly
☐	☒	The statistical test(s) used AND whether they are one- or two-sided<br>*Only common tests should be described solely by name; describe more complex techniques in the Methods section.*
☒	☐	A description of all covariates tested
☒	☐	A description of any assumptions or corrections, such as tests of normality and adjustment for multiple comparisons
☐	☒	A full description of the statistical parameters including central tendency (e.g. means) or other basic estimates (e.g. regression coefficient) AND variation (e.g. standard deviation) or associated estimates of uncertainty (e.g. confidence intervals)
☒	☐	For null hypothesis testing, the test statistic (e.g. *F*, *t*, *r*) with confidence intervals, effect sizes, degrees of freedom and *P* value noted<br>*Give P values as exact values whenever suitable.*
☒	☐	For Bayesian analysis, information on the choice of priors and Markov chain Monte Carlo settings
☒	☐	For hierarchical and complex designs, identification of the appropriate level for tests and full reporting of outcomes
☒	☐	Estimates of effect sizes (e.g. Cohen's *d*, Pearson's *r*), indicating how they were calculated

Our web collection on statistics for biologists contains articles on many of the points above.

Software and code

Policy information about availability of computer code

Data collection	No software was used
Data analysis	Most of the code for the present manuscript is based on Python 3.6 for Windows 10, and a few is given as MATLAB code based on MATLAB R2015a for Windows 10. All code for the reproduction of the quantitative results reported in the present study is available in: https://github.com/Network-Maritime-Complexity/Structural-core.

For manuscripts utilizing custom algorithms or software that are central to the research but not yet described in published literature, software must be made available to editors/reviewers. We strongly encourage code deposition in a community repository (e.g. GitHub). See the Nature Research guidelines for submitting code & software for further information.

Data

Policy information about availability of data

All manuscripts must include a data availability statement. This statement should provide the following information, where applicable:

- Accession codes, unique identifiers, or web links for publicly available datasets
- A list of figures that have associated raw data
- A description of any restrictions on data availability

The GLSN data and all other data supporting the findings of this study are available in: https://github.com/Network-Maritime-Complexity/Structural-core. This GitHub link contains the source data for Figs 1d, 2b-c, 5, and 9, Supplementary Figs 1b-c, 7a, 9, 10, 14, 18, 20, 24-25, 26b, Supplementary Tables 2, 4-10. Note that the source data for Fig 1d is a dataset on the GLSN topology of the year 2015. Raw data on world liner shipping services were provided by a third-party commercial database (Alphaliner, https://www.alphaliner.com/, one of the world's leading databases in the liner shipping industry) and were used under the license for the current study, and so are not publicly available. The dataset on the GLSN topology of the year 2017 is too recent and is not publicly available for the sake of the business of the database. All data generated during this study are however available from the corresponding authors on reasonable request. Data on the nautical distance between ports are publicly available in: https://www.searates.com/services/distances-time. Data on countries' international trade value and country pairs' bilateral trade value are publicly available in: https://comtrade.un.org/data.

Field-specific reporting

Please select the one below that is the best fit for your research. If you are not sure, read the appropriate sections before making your selection.

☐ Life sciences ☒ Behavioural & social sciences ☐ Ecological, evolutionary & environmental sciences

For a reference copy of the document with all sections, see nature.com/documents/nr-reporting-summary-flat.pdf

Behavioural & social sciences study design

All studies must disclose on these points even when the disclosure is negative.

Study description	Quantitative study of large empirical datasets on world maritime transportation (liner shipping)
Research sample	Two samples of the global liner shipping service routes were provided by a leading commercial database in liner shipping industry: Alphaliner, https://www.alphaliner.com/. The adopted samples include the liner shipping service routes of at least world's top 100 liner shipping companies (in terms of the liner shipping cargo carrying capacity), which altogether account for more than 92% of the world's total cargo carrying capacity in liner shipping.
Sampling strategy	The two samples contain 1622 and 1604 liner shipping service routes for the year of 2015 and 2017, respectively, and were all used in the analysis of the present study. For more details, see the Methods section.
Data collection	Mengqiao Xu collected the data samples of global liner shipping service routes from the Alphaliner database. Qian Pan collected the data on the international trade value of countries and the bilateral trade value of country pairs, from the UN Comtrade database (which is publicly available in the website link: https://comtrade.un.org/data/).
Timing	We obtained the two datasets on world's liner shipping service routes of the year 2015 and 2017 in April 2015 and November 2017, respectively. These two datasets are representative samples of world's liner shipping service routes in the respective years, as liner shipping service routes are regular and are prefixed by shipping companies. We downloaded the international trade datasets of the year 2015 and 2017 from the UN Comtrade database in July 2018 and March 2019, respectively.
Data exclusions	No data were excluded from the analyses.
Non-participation	No liner shipping service routes were dropped.
Randomization	Liner shipping service routes were not allocated into groups.

Reporting for specific materials, systems and methods

We require information from authors about some types of materials, experimental systems and methods used in many studies. Here, indicate whether each material, system or method listed is relevant to your study. If you are not sure if a list item applies to your research, read the appropriate section before selecting a response.

Materials & experimental systems

n/a	Involved in the study	
☒	☐	Antibodies
☒	☐	Eukaryotic cell lines
☒	☐	Palaeontology
☒	☐	Animals and other organisms
☒	☐	Human research participants
☒	☐	Clinical data

Methods

n/a	Involved in the study	
☒	☐	ChIP-seq
☒	☐	Flow cytometry
☒	☐	MRI-based neuroimaging
