# Supplementary figures and images for: Modular gateway-ness connectivity and structural core organization in maritime network science
Source: Nat Commun. 2020 Jun 5;11:2849. doi: 10.1038/s41467-020-16619-5 (PMC7275034; doi:10.1038/s41467-020-16619-5)

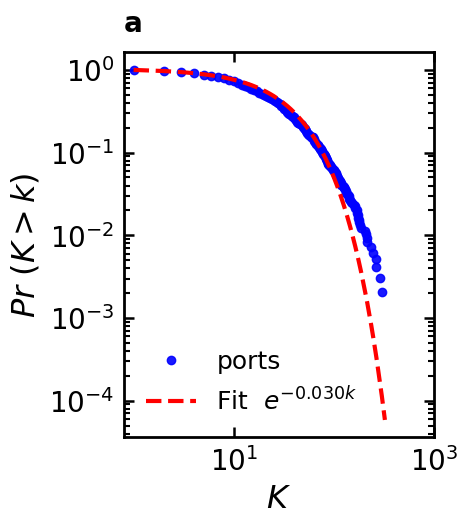

Supplement: Supplementary file 4 — Source data [file 41467_2020_16619_MOESM4_ESM.zip › Structural-core-master/code/Article code/Expected output/Basic_topological_properties_and_economic_small_world_ness/Fig. 2 Basic topological properties of the GLSN (a).png]

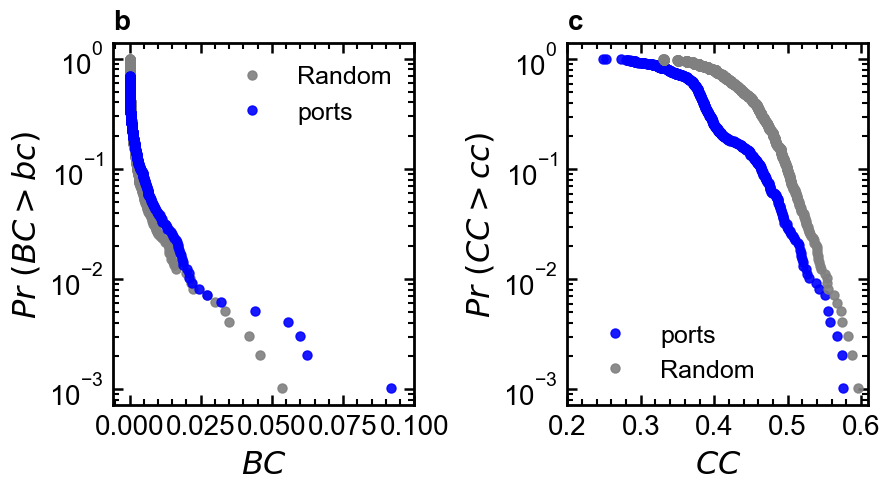

Supplement: Supplementary file 4 — Source data [file 41467_2020_16619_MOESM4_ESM.zip › Structural-core-master/code/Article code/Expected output/Basic_topological_properties_and_economic_small_world_ness/Fig. 2 Basic topological properties of the GLSN (b) and (c).png]

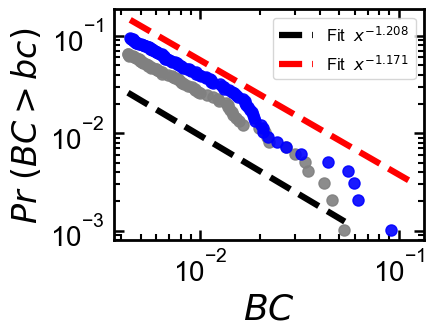

Supplement: Supplementary file 4 — Source data [file 41467_2020_16619_MOESM4_ESM.zip › Structural-core-master/code/Article code/Expected output/Basic_topological_properties_and_economic_small_world_ness/Fig. 2 Basic topological properties of the GLSN (b) subplot.png]

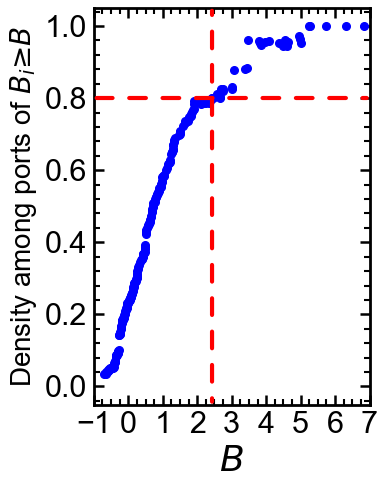

Supplement: Supplementary file 4 — Source data [file 41467_2020_16619_MOESM4_ESM.zip › Structural-core-master/code/Article code/Expected output/Gateway_hub_structural_core/Fig. 6 Structural core detection of the GLSN (a).png]

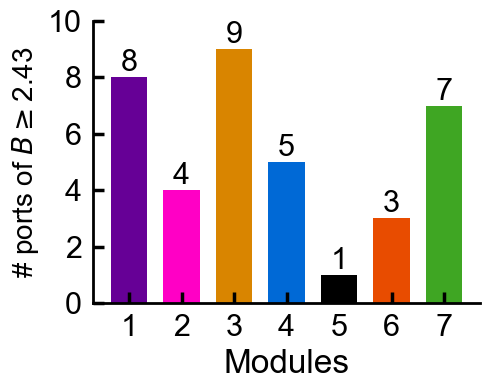

Supplement: Supplementary file 4 — Source data [file 41467_2020_16619_MOESM4_ESM.zip › Structural-core-master/code/Article code/Expected output/Gateway_hub_structural_core/Fig. 6 Structural core detection of the GLSN (b).png]

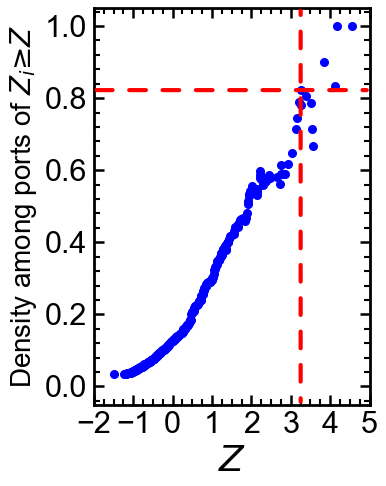

Supplement: Supplementary file 4 — Source data [file 41467_2020_16619_MOESM4_ESM.zip › Structural-core-master/code/Article code/Expected output/Gateway_hub_structural_core/Fig. 6 Structural core detection of the GLSN (c).png]

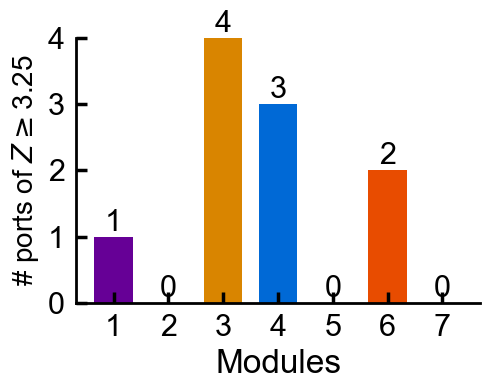

Supplement: Supplementary file 4 — Source data [file 41467_2020_16619_MOESM4_ESM.zip › Structural-core-master/code/Article code/Expected output/Gateway_hub_structural_core/Fig. 6 Structural core detection of the GLSN (d).png]

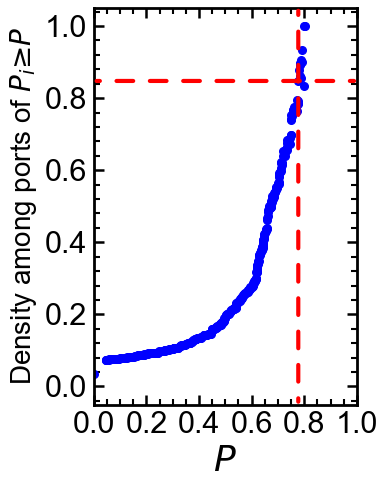

Supplement: Supplementary file 4 — Source data [file 41467_2020_16619_MOESM4_ESM.zip › Structural-core-master/code/Article code/Expected output/Gateway_hub_structural_core/Fig. 6 Structural core detection of the GLSN (e).png]

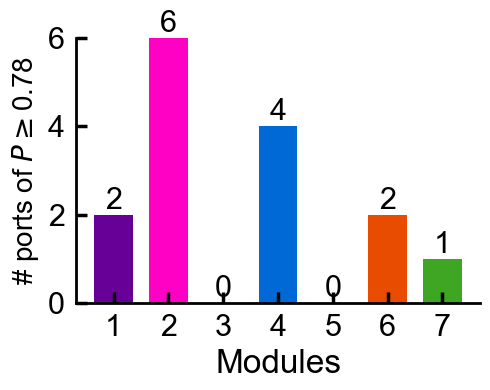

Supplement: Supplementary file 4 — Source data [file 41467_2020_16619_MOESM4_ESM.zip › Structural-core-master/code/Article code/Expected output/Gateway_hub_structural_core/Fig. 6 Structural core detection of the GLSN (f).png]

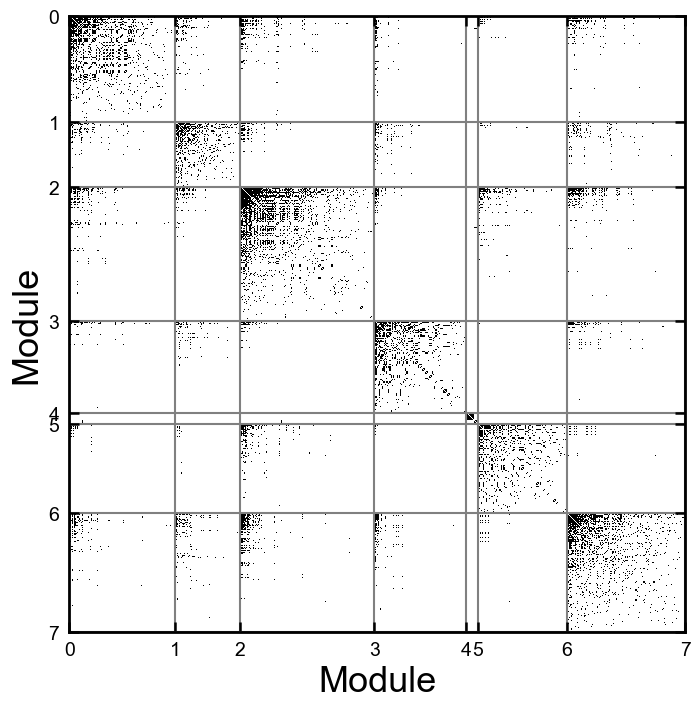

Supplement: Supplementary file 4 — Source data [file 41467_2020_16619_MOESM4_ESM.zip › Structural-core-master/code/Article code/Expected output/Multiscale_modularity_and_hubs_diversity/Fig. 3 Multiscale modular communities in the GLSN (e).png]

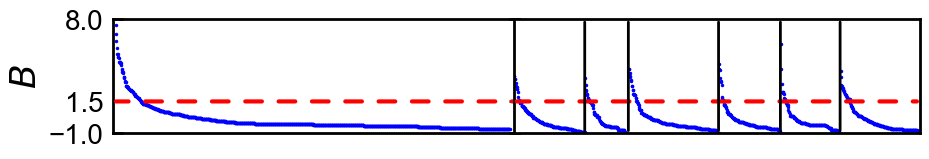

Supplement: Supplementary file 4 — Source data [file 41467_2020_16619_MOESM4_ESM.zip › Structural-core-master/code/Article code/Expected output/Multiscale_modularity_and_hubs_diversity/Fig. 4 Ports' outside-module degree... B.png]

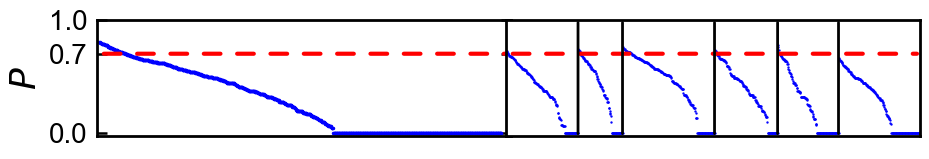

Supplement: Supplementary file 4 — Source data [file 41467_2020_16619_MOESM4_ESM.zip › Structural-core-master/code/Article code/Expected output/Multiscale_modularity_and_hubs_diversity/Fig. 4 Ports' outside-module degree... P.png]

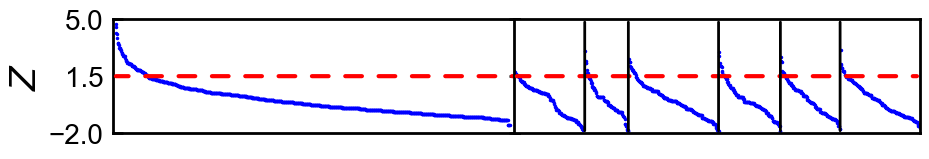

Supplement: Supplementary file 4 — Source data [file 41467_2020_16619_MOESM4_ESM.zip › Structural-core-master/code/Article code/Expected output/Multiscale_modularity_and_hubs_diversity/Fig. 4 Ports' outside-module degree... Z.png]

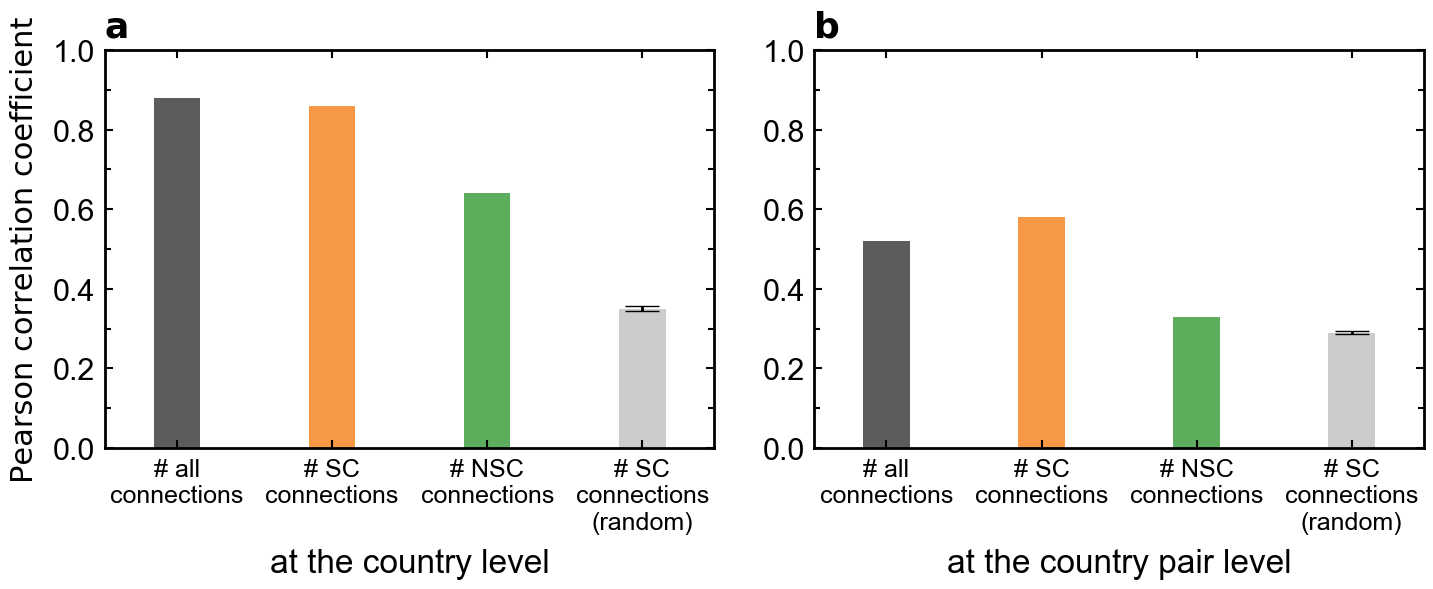

Supplement: Supplementary file 4 — Source data [file 41467_2020_16619_MOESM4_ESM.zip › Structural-core-master/code/Article code/Expected output/Structural_core_and_international_trade/Fig. 9 Correlation between the GLSN topological indicators and international trade indicators of countries.png]

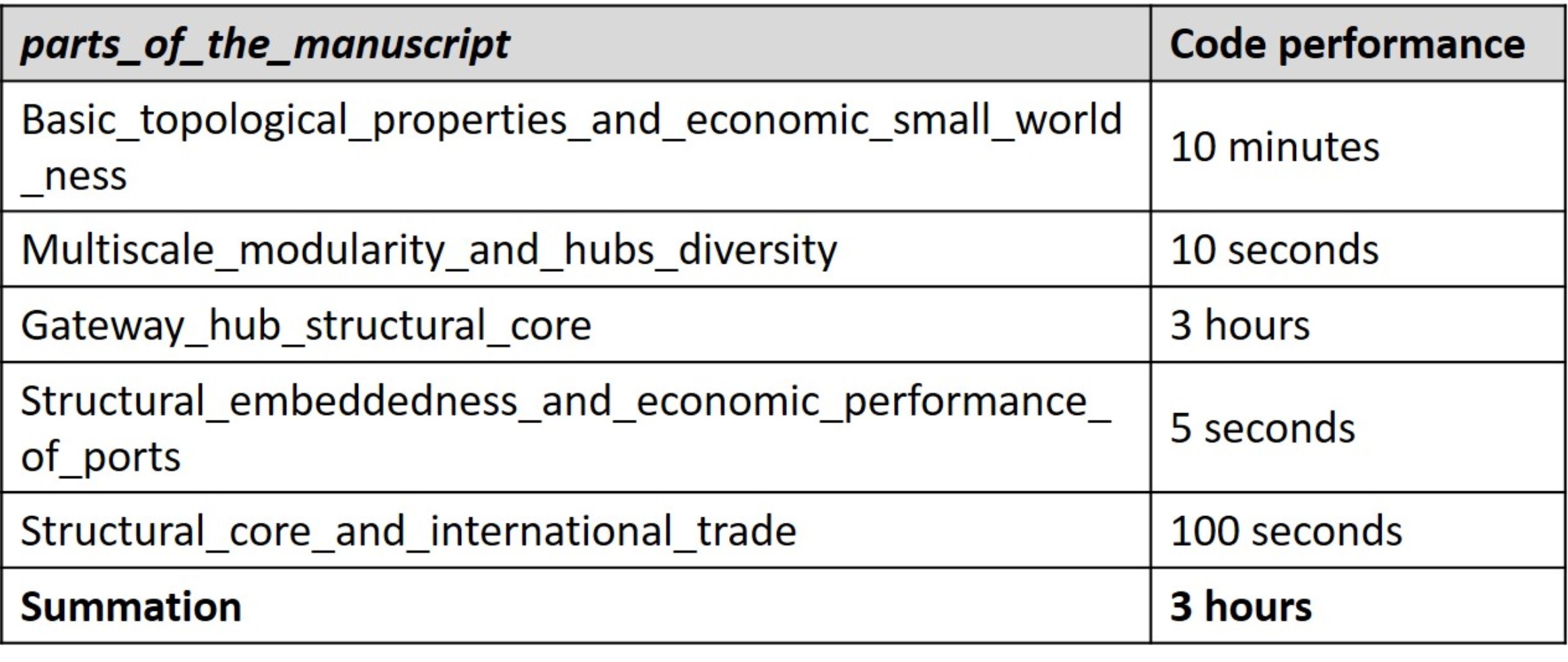

Supplement: Supplementary file 4 — Source data [file 41467_2020_16619_MOESM4_ESM.zip › Structural-core-master/code/Code performance (Article).jpg]

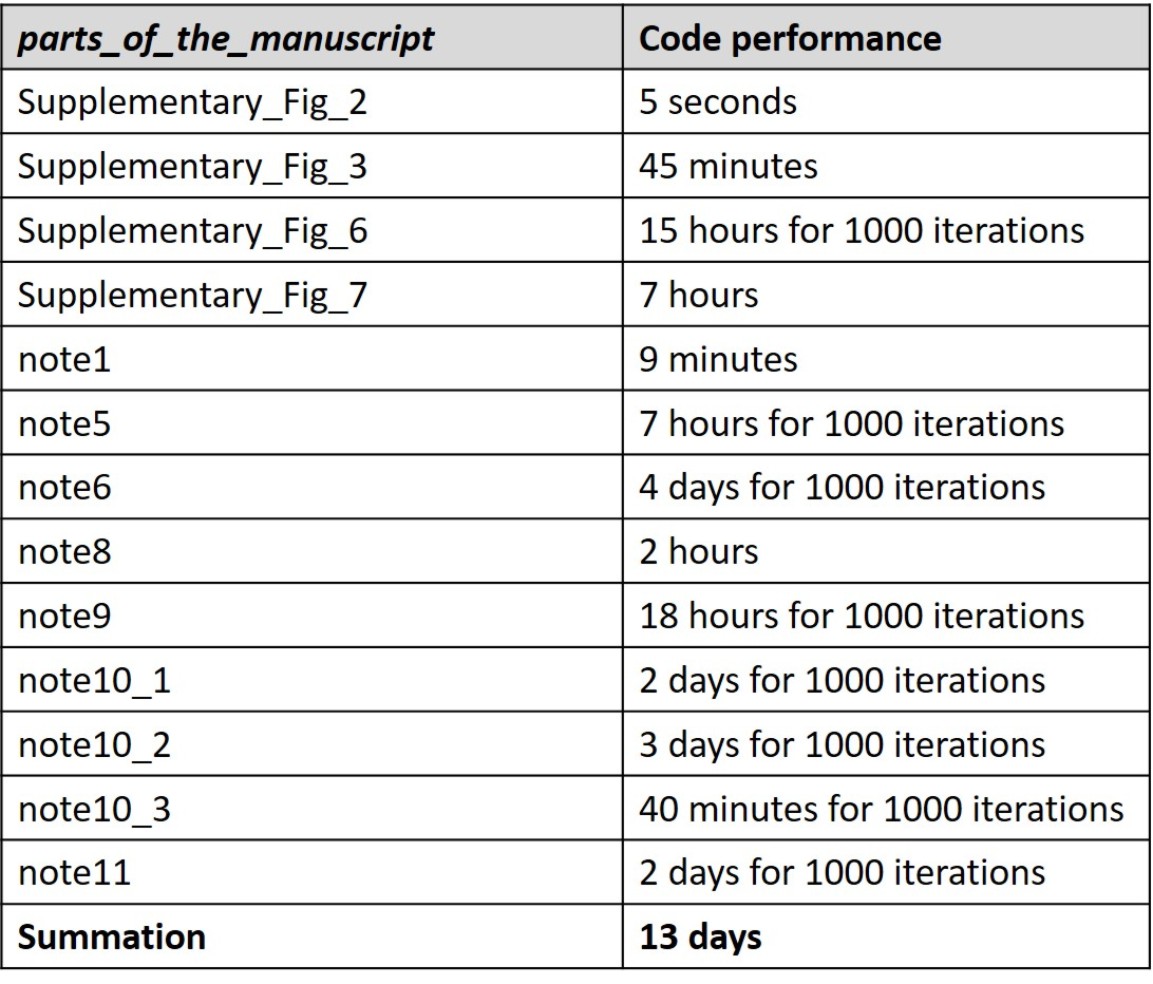

Supplement: Supplementary file 4 — Source data [file 41467_2020_16619_MOESM4_ESM.zip › Structural-core-master/code/Code performance (Supplementary information).jpg]

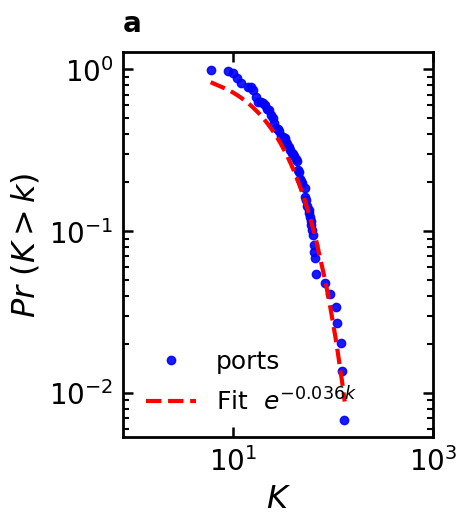

Supplement: Supplementary file 4 — Source data [file 41467_2020_16619_MOESM4_ESM.zip › Structural-core-master/code/Demo/Expected output/Basic_topological_properties_and_economic_small_world_ness/Fig. 2 Basic topological properties of the GLSN (a).png]

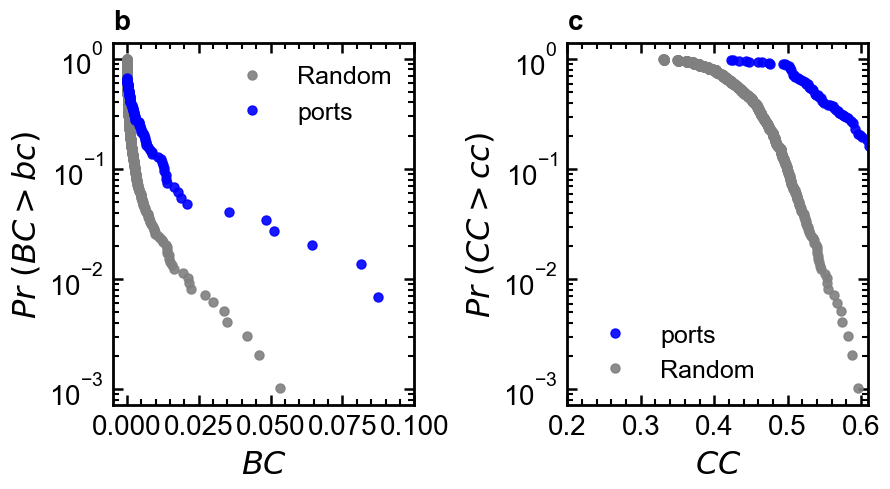

Supplement: Supplementary file 4 — Source data [file 41467_2020_16619_MOESM4_ESM.zip › Structural-core-master/code/Demo/Expected output/Basic_topological_properties_and_economic_small_world_ness/Fig. 2 Basic topological properties of the GLSN (b) and (c).png]

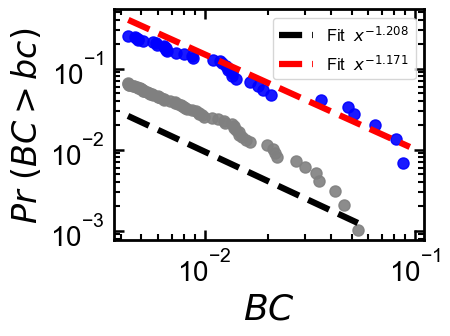

Supplement: Supplementary file 4 — Source data [file 41467_2020_16619_MOESM4_ESM.zip › Structural-core-master/code/Demo/Expected output/Basic_topological_properties_and_economic_small_world_ness/Fig. 2 Basic topological properties of the GLSN (b) subplot.png]

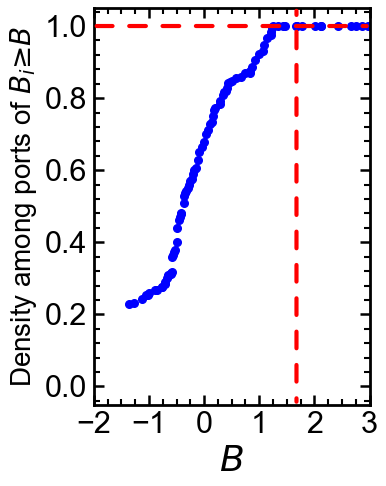

Supplement: Supplementary file 4 — Source data [file 41467_2020_16619_MOESM4_ESM.zip › Structural-core-master/code/Demo/Expected output/Gateway_hub_structural_core/Fig. 6 Structural core detection of the GLSN (a).png]

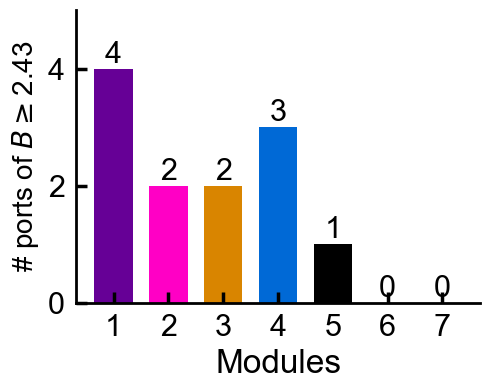

Supplement: Supplementary file 4 — Source data [file 41467_2020_16619_MOESM4_ESM.zip › Structural-core-master/code/Demo/Expected output/Gateway_hub_structural_core/Fig. 6 Structural core detection of the GLSN (b).png]

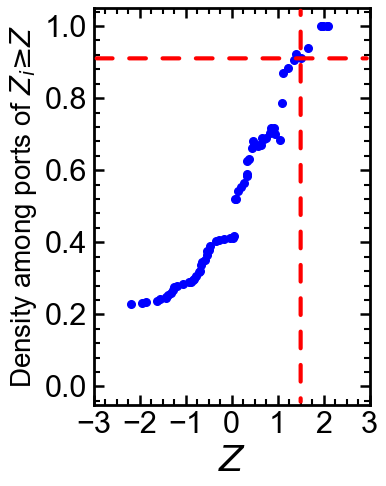

Supplement: Supplementary file 4 — Source data [file 41467_2020_16619_MOESM4_ESM.zip › Structural-core-master/code/Demo/Expected output/Gateway_hub_structural_core/Fig. 6 Structural core detection of the GLSN (c).png]

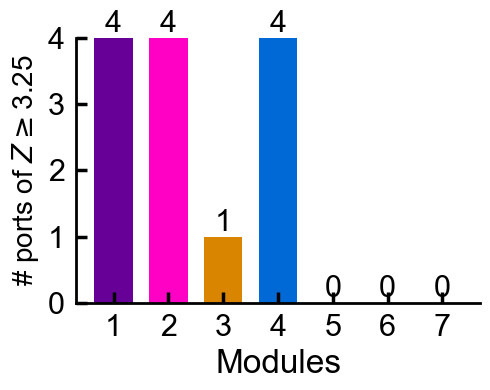

Supplement: Supplementary file 4 — Source data [file 41467_2020_16619_MOESM4_ESM.zip › Structural-core-master/code/Demo/Expected output/Gateway_hub_structural_core/Fig. 6 Structural core detection of the GLSN (d).png]

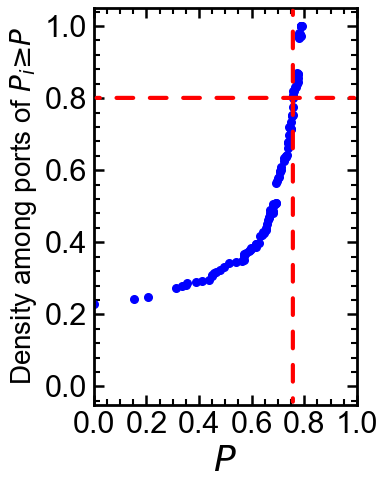

Supplement: Supplementary file 4 — Source data [file 41467_2020_16619_MOESM4_ESM.zip › Structural-core-master/code/Demo/Expected output/Gateway_hub_structural_core/Fig. 6 Structural core detection of the GLSN (e).png]

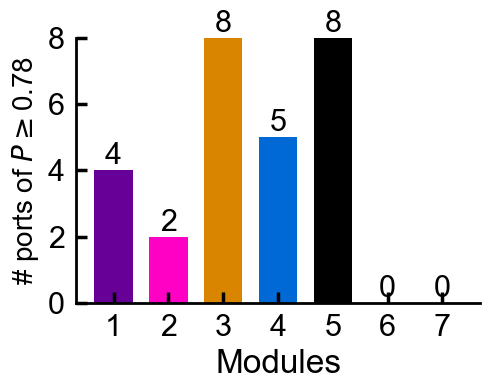

Supplement: Supplementary file 4 — Source data [file 41467_2020_16619_MOESM4_ESM.zip › Structural-core-master/code/Demo/Expected output/Gateway_hub_structural_core/Fig. 6 Structural core detection of the GLSN (f).png]

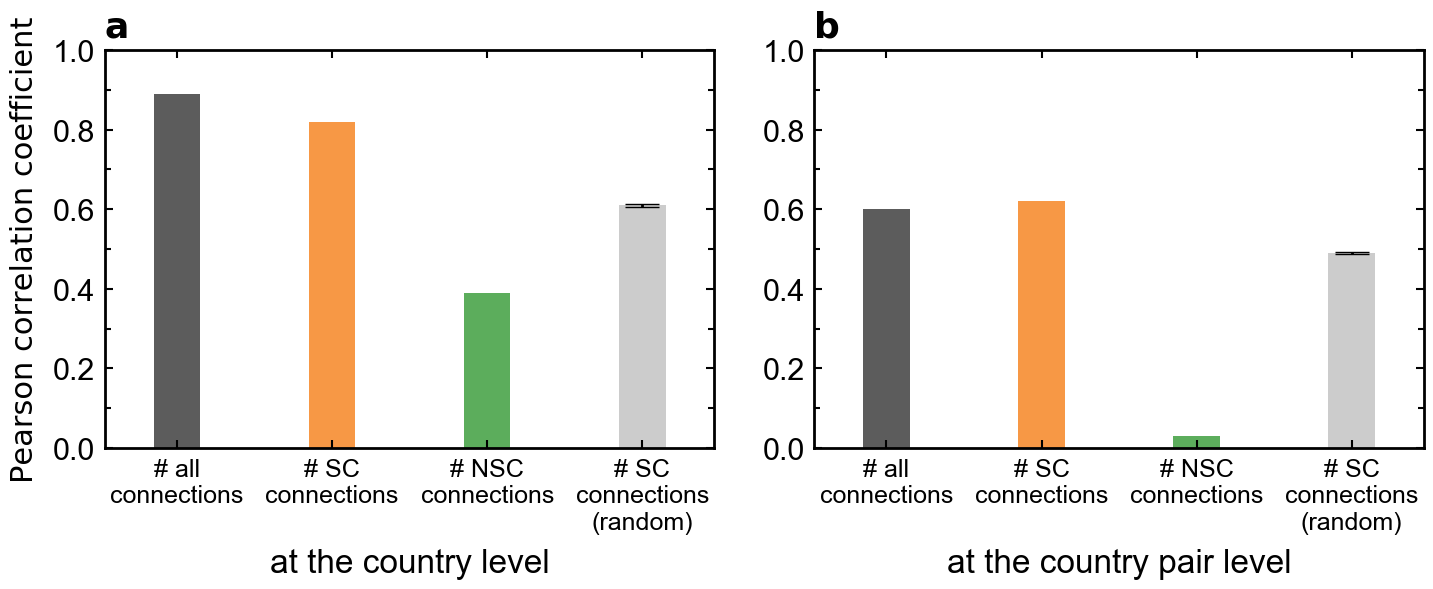

Supplement: Supplementary file 4 — Source data [file 41467_2020_16619_MOESM4_ESM.zip › Structural-core-master/code/Demo/Expected output/Structural_core_and_international_trade/Fig. 9 Correlation between the GLSN topological indicators and international trade indicators of countries.png]

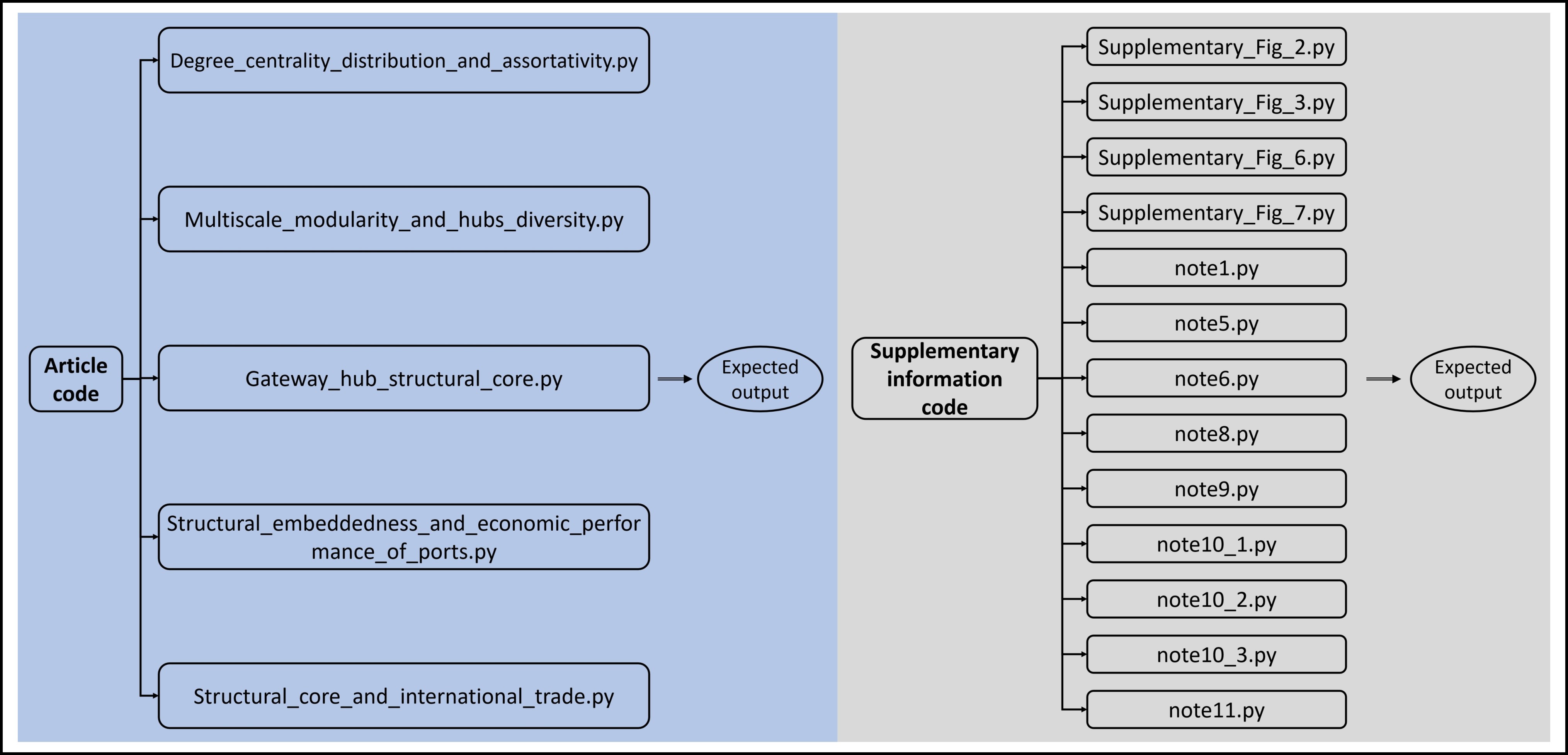

Supplement: Supplementary file 4 — Source data [file 41467_2020_16619_MOESM4_ESM.zip › Structural-core-master/code/Overview.jpg]

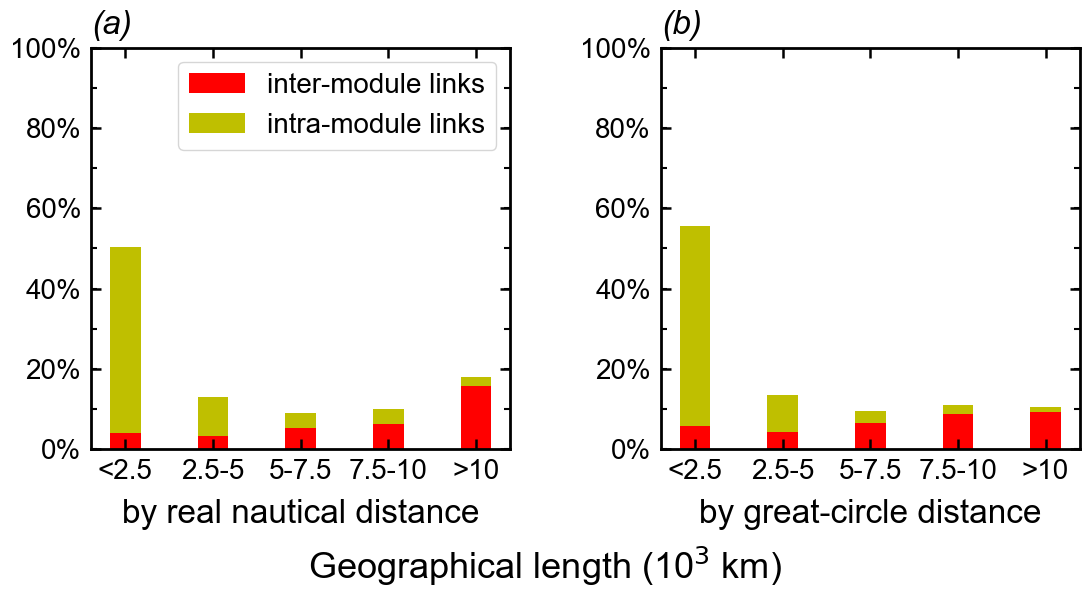

Supplement: Supplementary file 4 — Source data [file 41467_2020_16619_MOESM4_ESM.zip › Structural-core-master/code/Supplementary information code/Expected output/Supplementary Fig. 2 Proportional distribution of intra- and inter- module links in different range of geographical length.png]

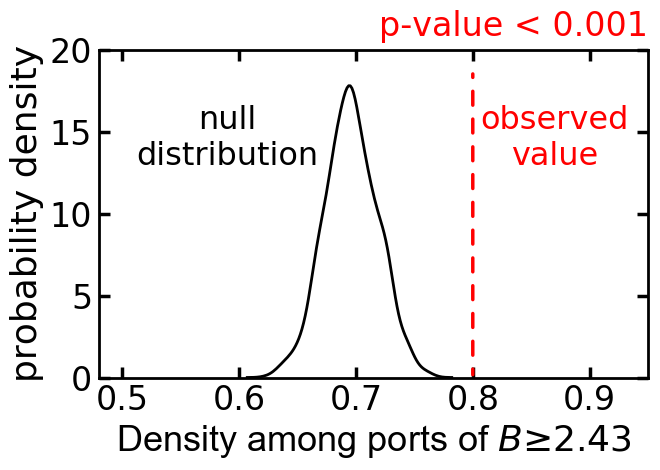

Supplement: Supplementary file 4 — Source data [file 41467_2020_16619_MOESM4_ESM.zip › Structural-core-master/code/Supplementary information code/Expected output/Supplementary Fig. 3 Statistical significance of the structural core in the real GLSN of 2015 (a) Lower.png]

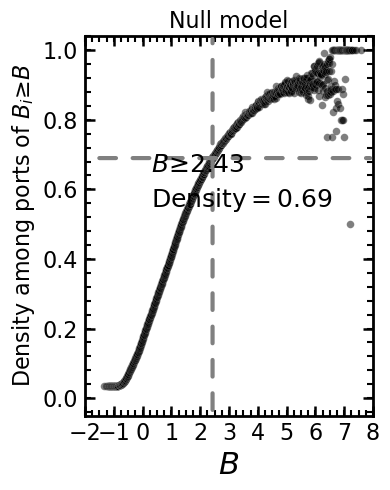

Supplement: Supplementary file 4 — Source data [file 41467_2020_16619_MOESM4_ESM.zip › Structural-core-master/code/Supplementary information code/Expected output/Supplementary Fig. 3 Statistical significance of the structural core in the real GLSN of 2015 (a) Upper Left.png]

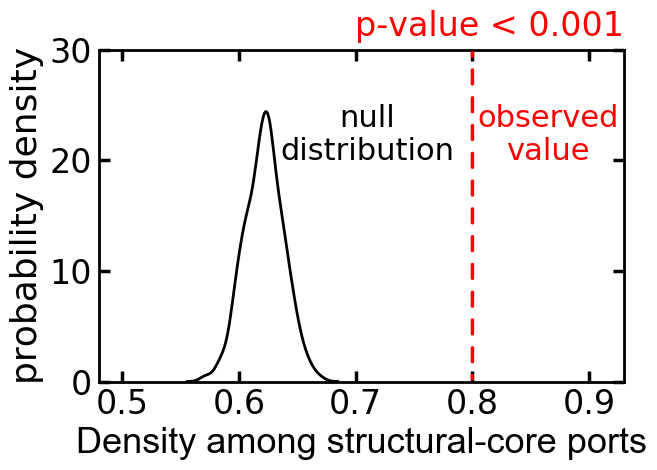

Supplement: Supplementary file 4 — Source data [file 41467_2020_16619_MOESM4_ESM.zip › Structural-core-master/code/Supplementary information code/Expected output/Supplementary Fig. 3 Statistical significance of the structural core in the real GLSN of 2015 (b) Lower.png]

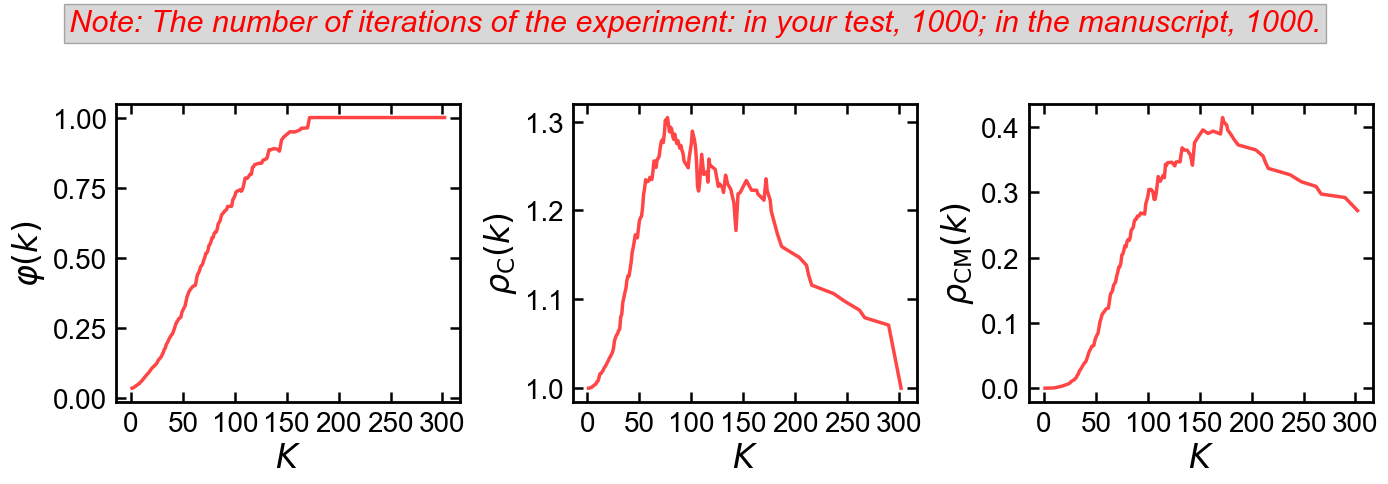

Supplement: Supplementary file 4 — Source data [file 41467_2020_16619_MOESM4_ESM.zip › Structural-core-master/code/Supplementary information code/Expected output/Supplementary Fig. 6 Rich-club coefficients of world ports.png]

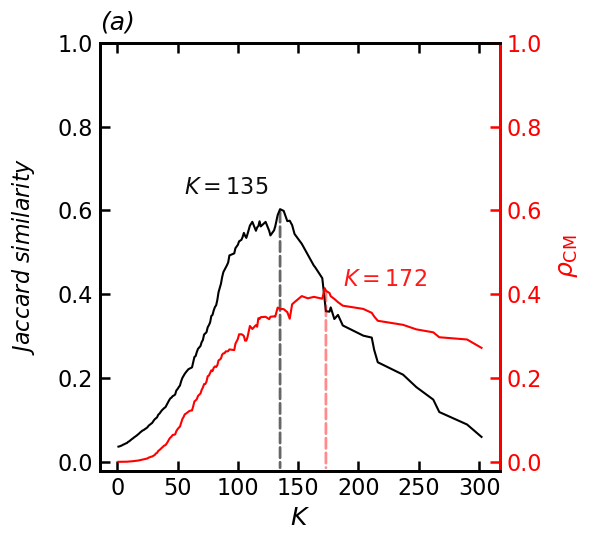

Supplement: Supplementary file 4 — Source data [file 41467_2020_16619_MOESM4_ESM.zip › Structural-core-master/code/Supplementary information code/Expected output/Supplementary Fig. 7 Overlap between the rich club and the structural core of the GLSN (a).png]

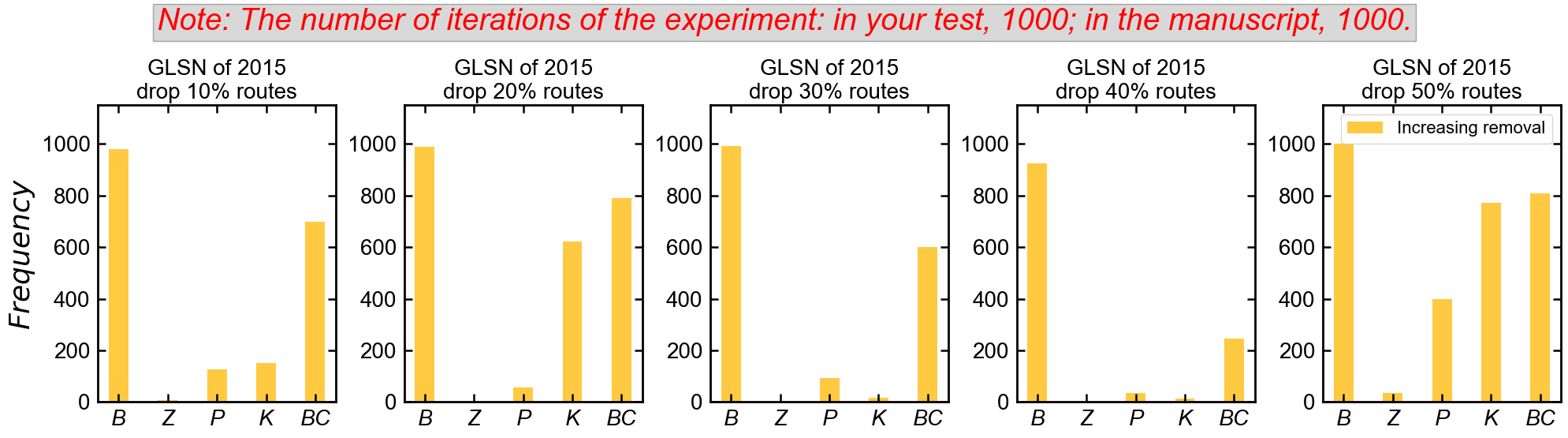

Supplement: Supplementary file 4 — Source data [file 41467_2020_16619_MOESM4_ESM.zip › Structural-core-master/code/Supplementary information code/Expected output/Supplementary note 10_1/Supplementary Fig. 19 Frequency...removed routes-Increasing removal-(a) GLSN of 2015.png]

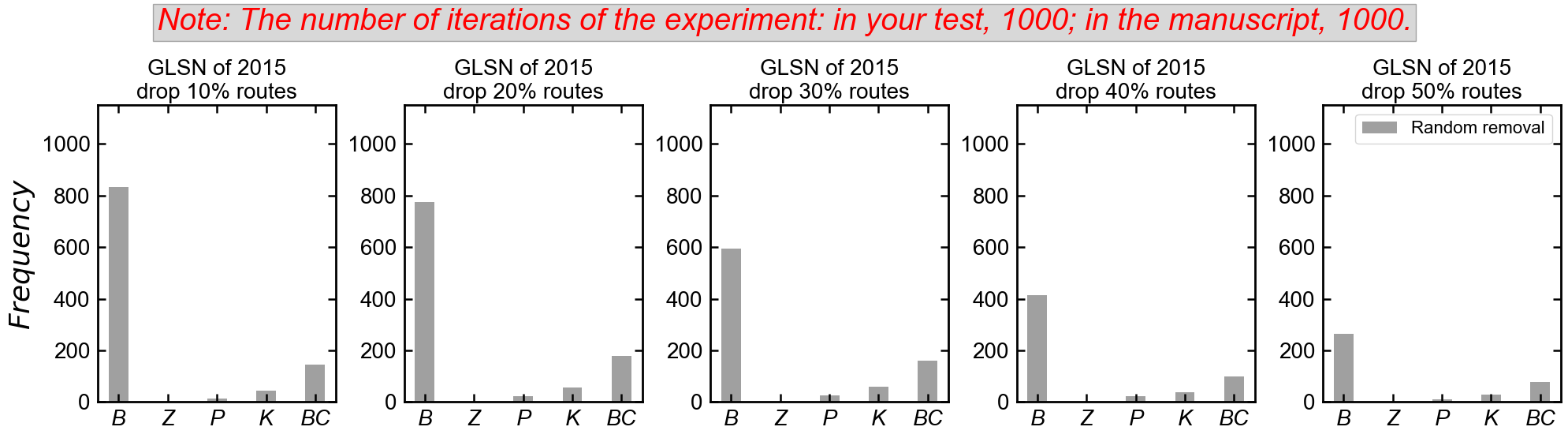

Supplement: Supplementary file 4 — Source data [file 41467_2020_16619_MOESM4_ESM.zip › Structural-core-master/code/Supplementary information code/Expected output/Supplementary note 10_1/Supplementary Fig. 19 Frequency...removed routes-random removal-(a) GLSN of 2015.png]

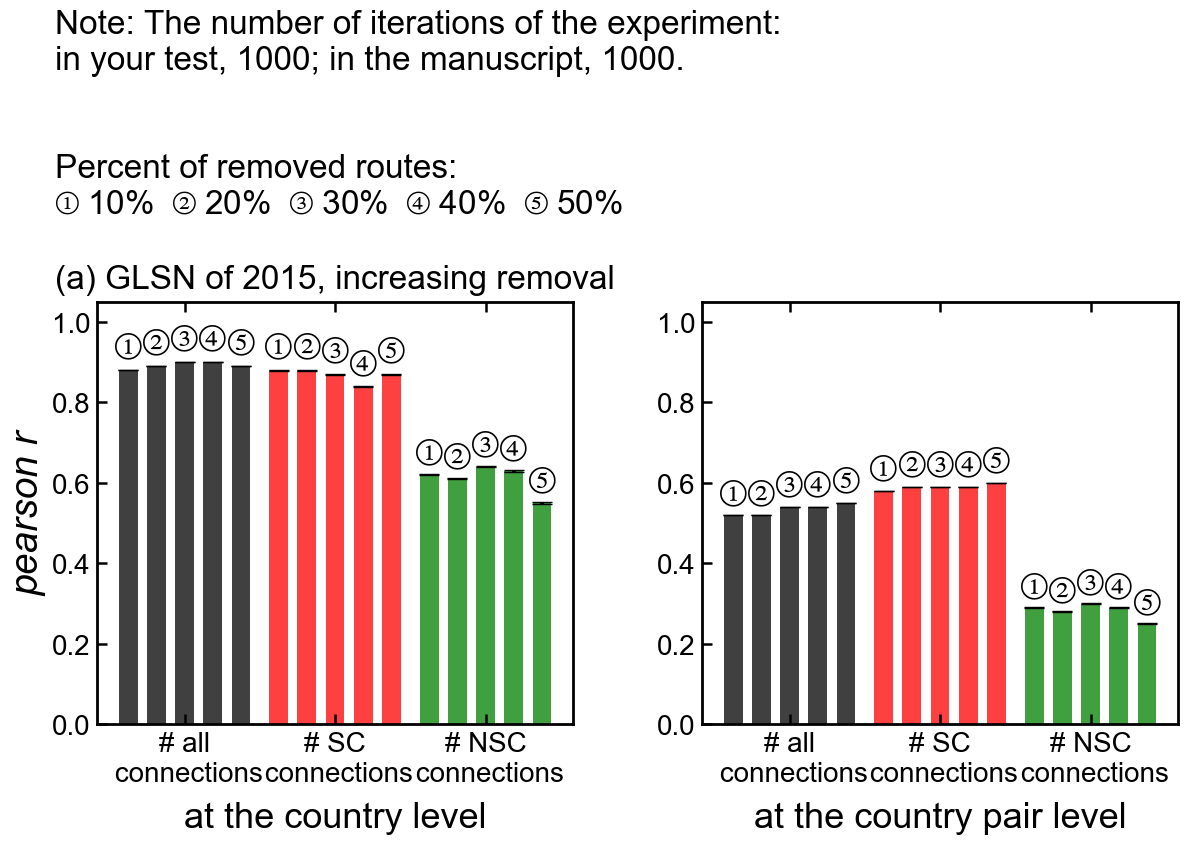

Supplement: Supplementary file 4 — Source data [file 41467_2020_16619_MOESM4_ESM.zip › Structural-core-master/code/Supplementary information code/Expected output/Supplementary note 10_1/Supplementary Fig. 20 Pearson correlation coefficients...removed routes-(a).png]

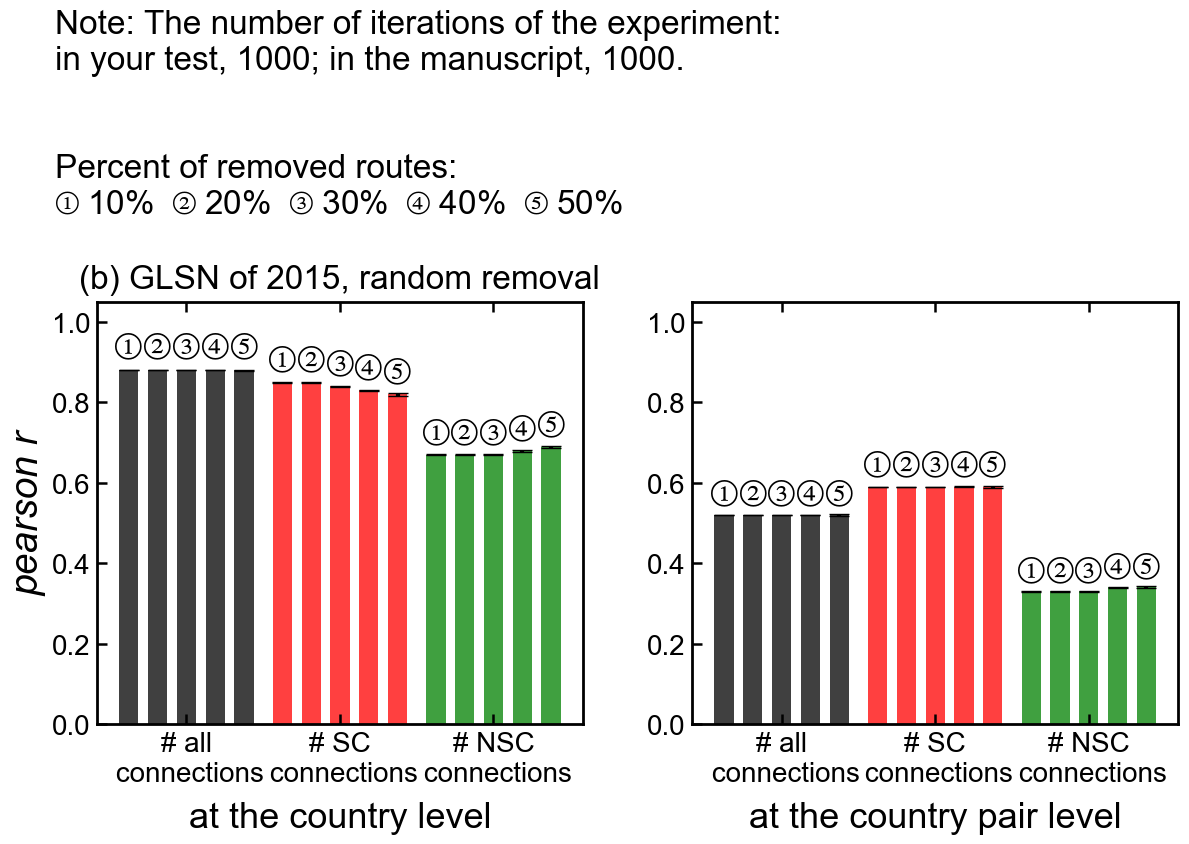

Supplement: Supplementary file 4 — Source data [file 41467_2020_16619_MOESM4_ESM.zip › Structural-core-master/code/Supplementary information code/Expected output/Supplementary note 10_1/Supplementary Fig. 20 Pearson correlation coefficients...removed routes-(b).png]

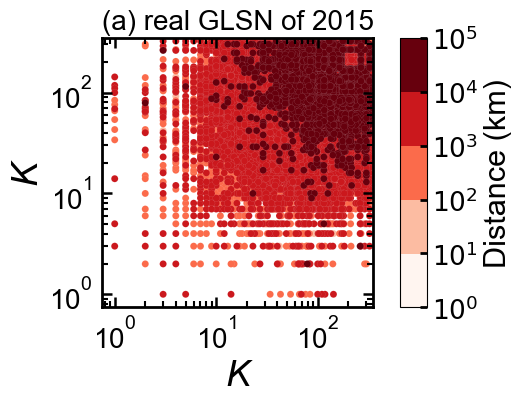

Supplement: Supplementary file 4 — Source data [file 41467_2020_16619_MOESM4_ESM.zip › Structural-core-master/code/Supplementary information code/Expected output/Supplementary note 10_2/Supplementary Fig. 21 Edge distributions...(a).png]

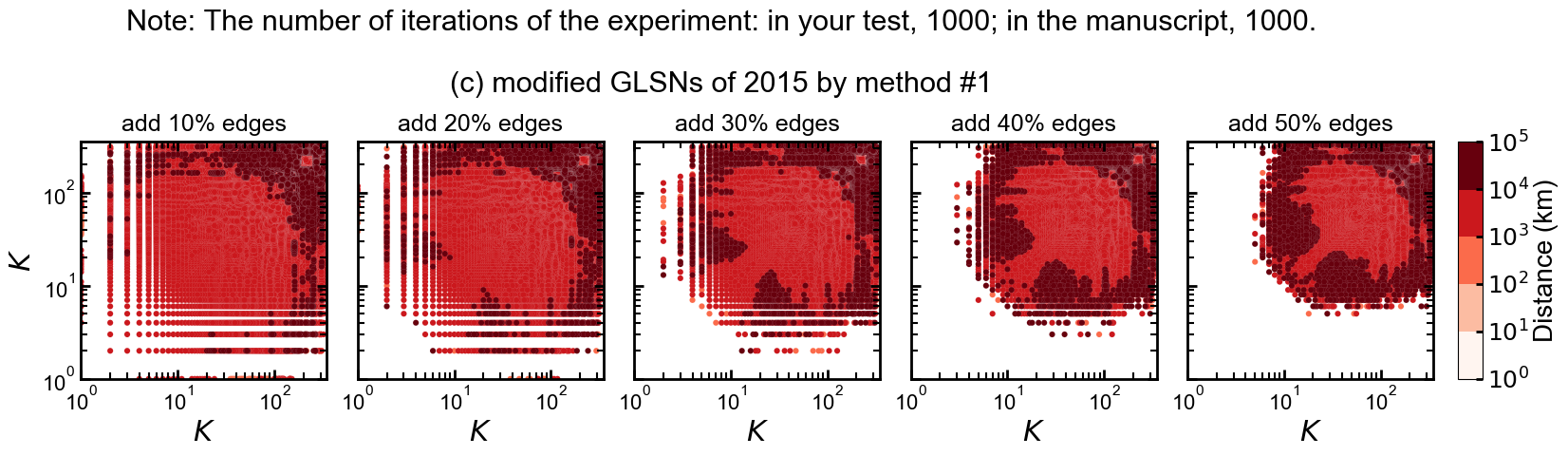

Supplement: Supplementary file 4 — Source data [file 41467_2020_16619_MOESM4_ESM.zip › Structural-core-master/code/Supplementary information code/Expected output/Supplementary note 10_2/Supplementary Fig. 21 Edge distributions...(c).png]

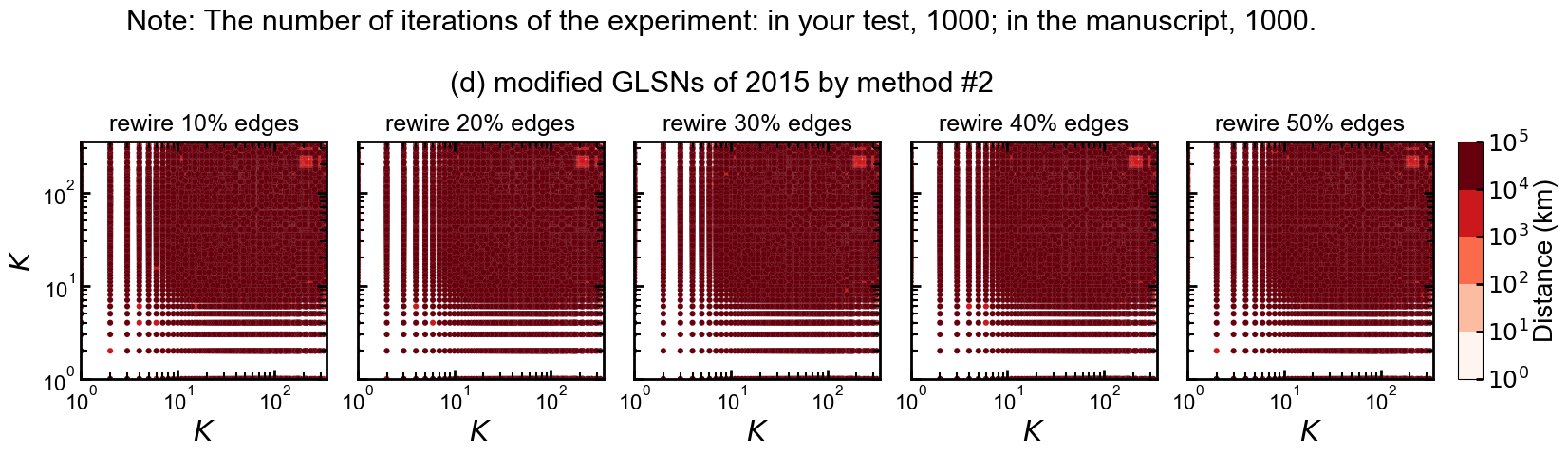

Supplement: Supplementary file 4 — Source data [file 41467_2020_16619_MOESM4_ESM.zip › Structural-core-master/code/Supplementary information code/Expected output/Supplementary note 10_2/Supplementary Fig. 21 Edge distributions...(d).png]

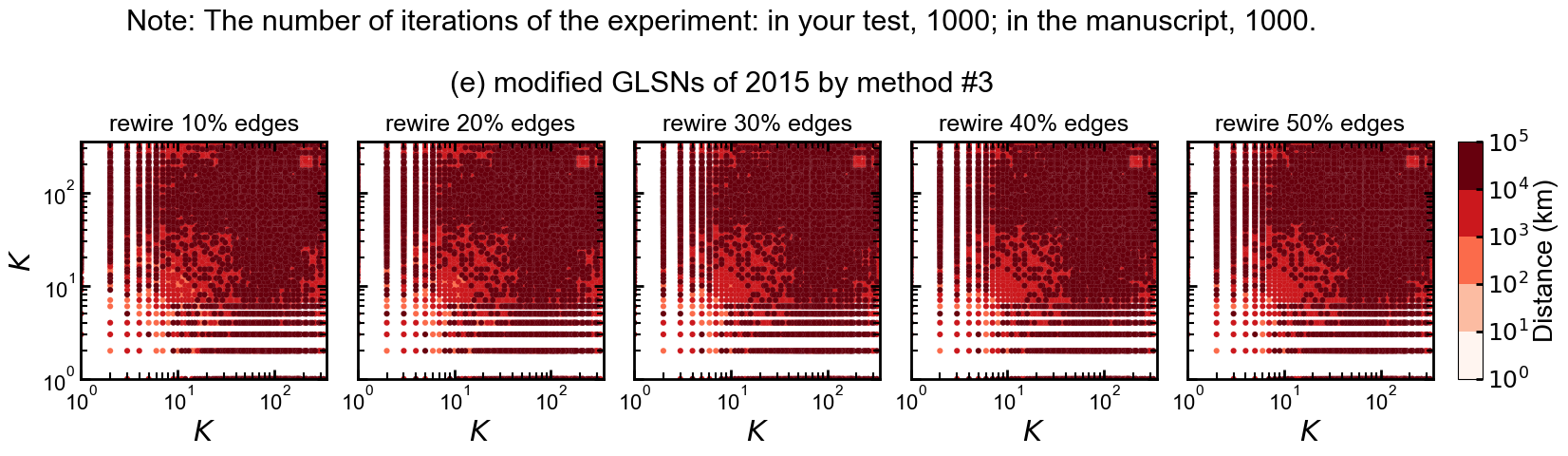

Supplement: Supplementary file 4 — Source data [file 41467_2020_16619_MOESM4_ESM.zip › Structural-core-master/code/Supplementary information code/Expected output/Supplementary note 10_2/Supplementary Fig. 21 Edge distributions...(e).png]

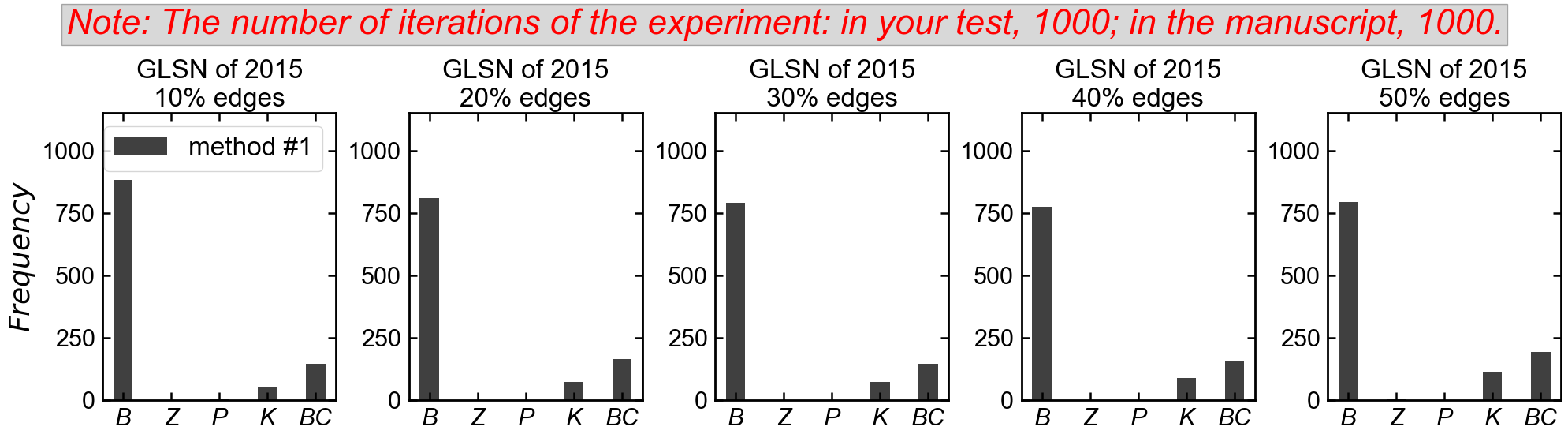

Supplement: Supplementary file 4 — Source data [file 41467_2020_16619_MOESM4_ESM.zip › Structural-core-master/code/Supplementary information code/Expected output/Supplementary note 10_2/Supplementary Fig. 23 Frequency...percent of edges-method #1-(a) GLSN of 2015.png]

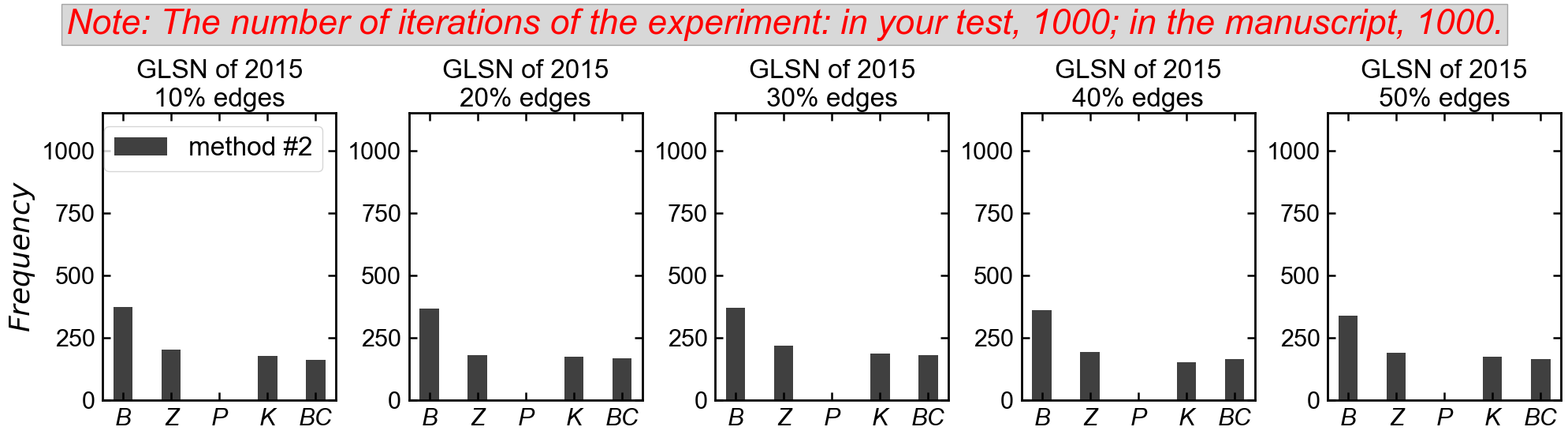

Supplement: Supplementary file 4 — Source data [file 41467_2020_16619_MOESM4_ESM.zip › Structural-core-master/code/Supplementary information code/Expected output/Supplementary note 10_2/Supplementary Fig. 23 Frequency...percent of edges-method #2-(a) GLSN of 2015.png]

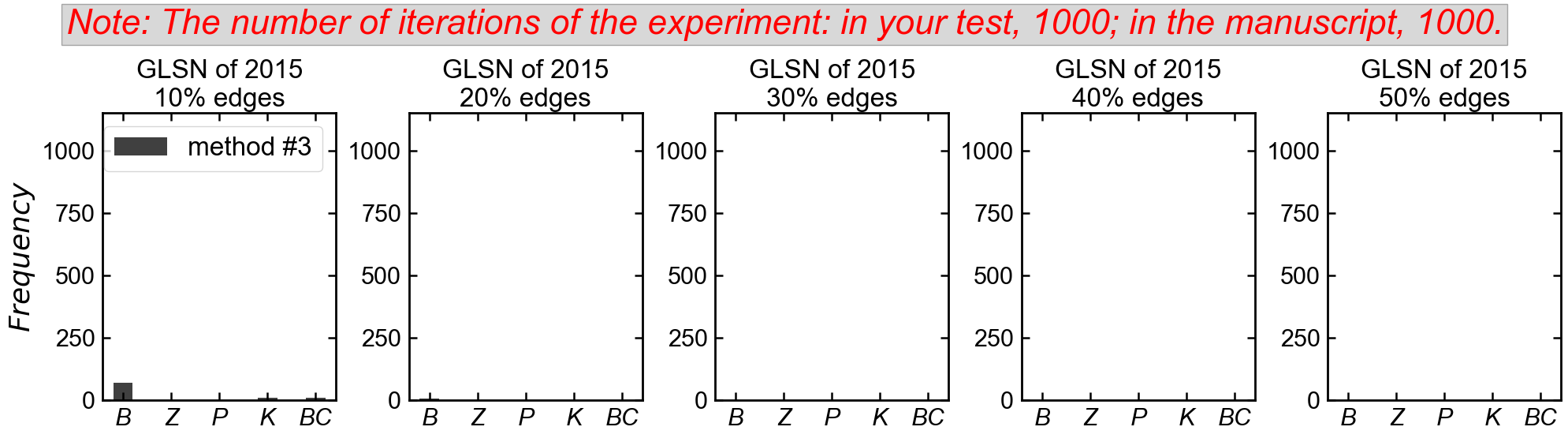

Supplement: Supplementary file 4 — Source data [file 41467_2020_16619_MOESM4_ESM.zip › Structural-core-master/code/Supplementary information code/Expected output/Supplementary note 10_2/Supplementary Fig. 23 Frequency...percent of edges-method #3-(a) GLSN of 2015.png]

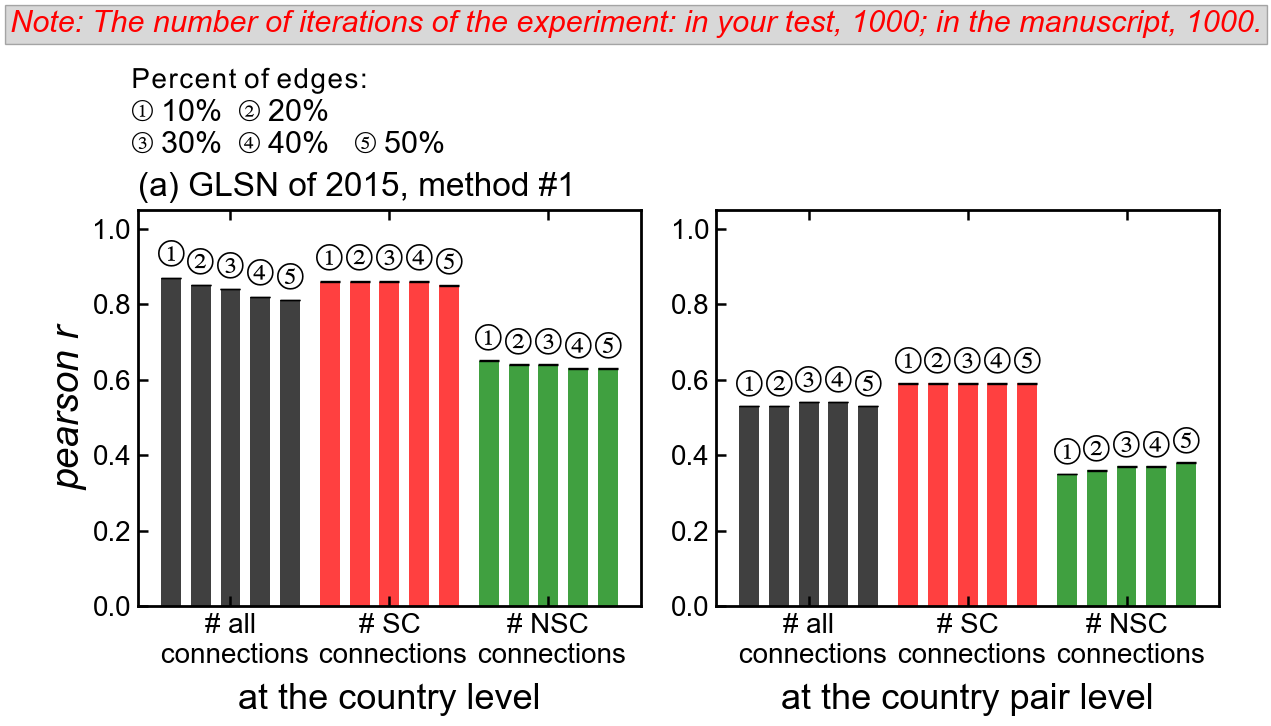

Supplement: Supplementary file 4 — Source data [file 41467_2020_16619_MOESM4_ESM.zip › Structural-core-master/code/Supplementary information code/Expected output/Supplementary note 10_2/Supplementary Fig. 24 Pearson correlation coefficients...2015 dataset (a).png]

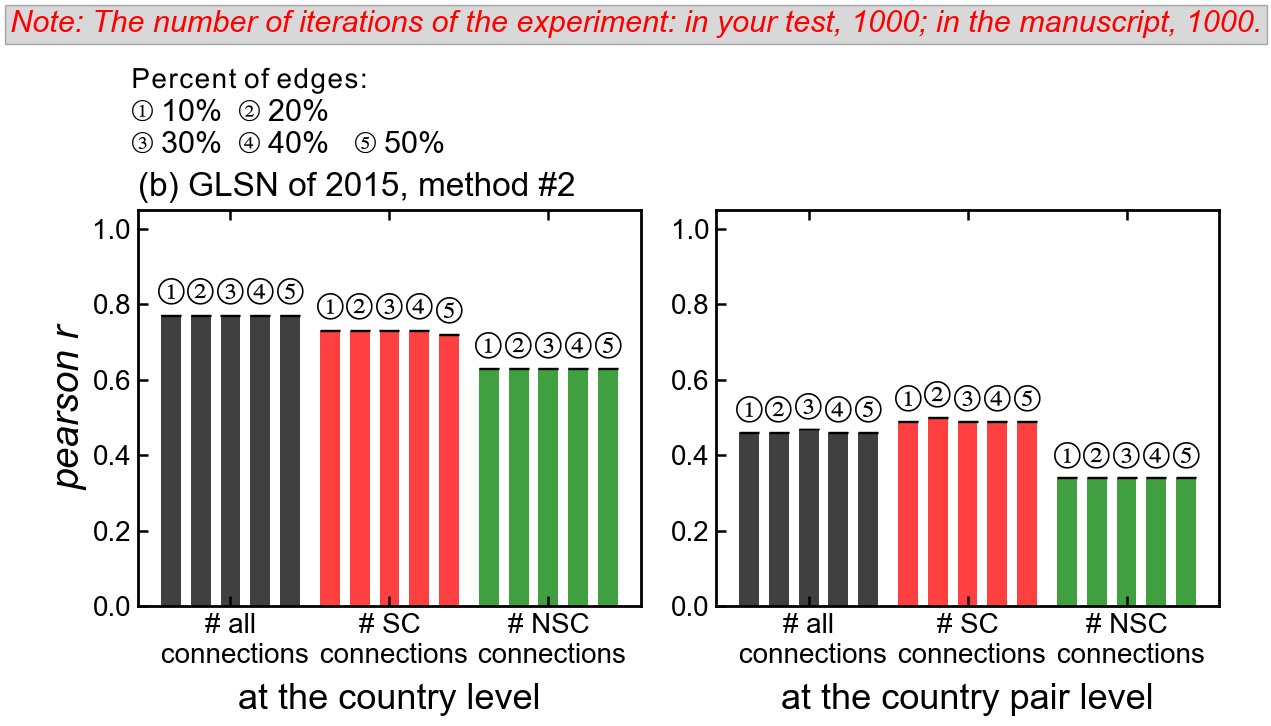

Supplement: Supplementary file 4 — Source data [file 41467_2020_16619_MOESM4_ESM.zip › Structural-core-master/code/Supplementary information code/Expected output/Supplementary note 10_2/Supplementary Fig. 24 Pearson correlation coefficients...2015 dataset (b).png]

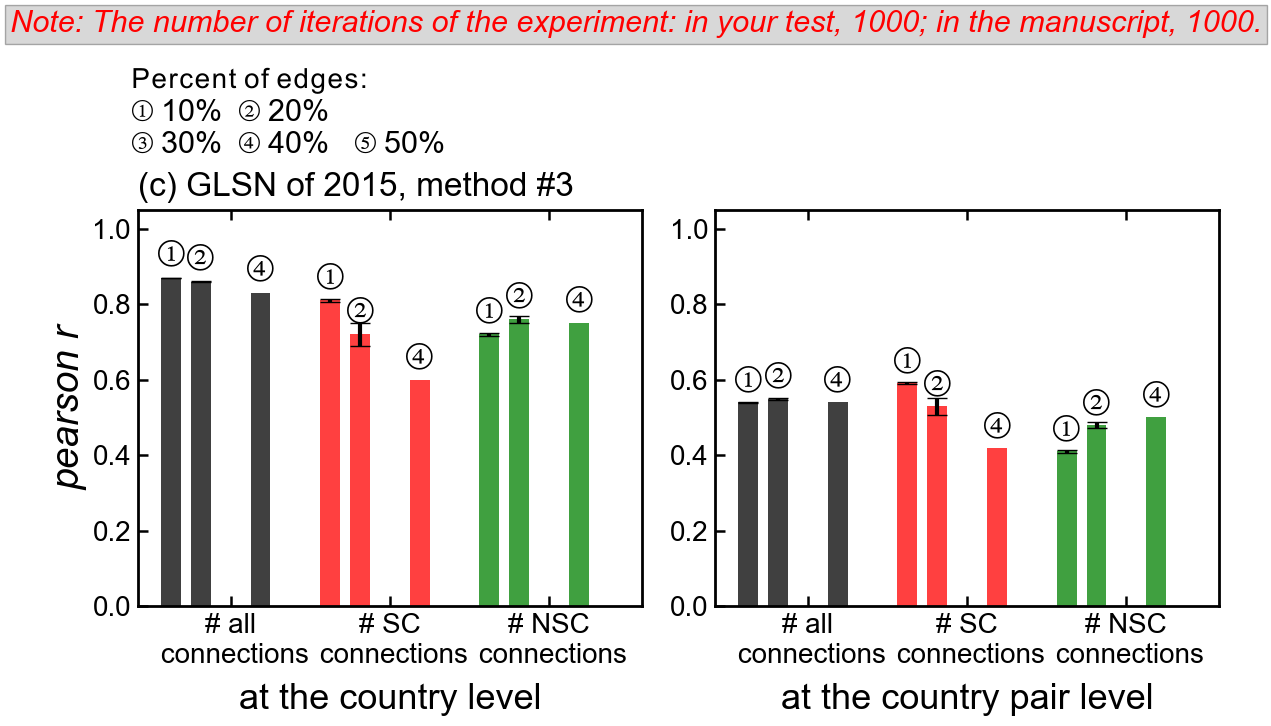

Supplement: Supplementary file 4 — Source data [file 41467_2020_16619_MOESM4_ESM.zip › Structural-core-master/code/Supplementary information code/Expected output/Supplementary note 10_2/Supplementary Fig. 24 Pearson correlation coefficients...2015 dataset (c).png]

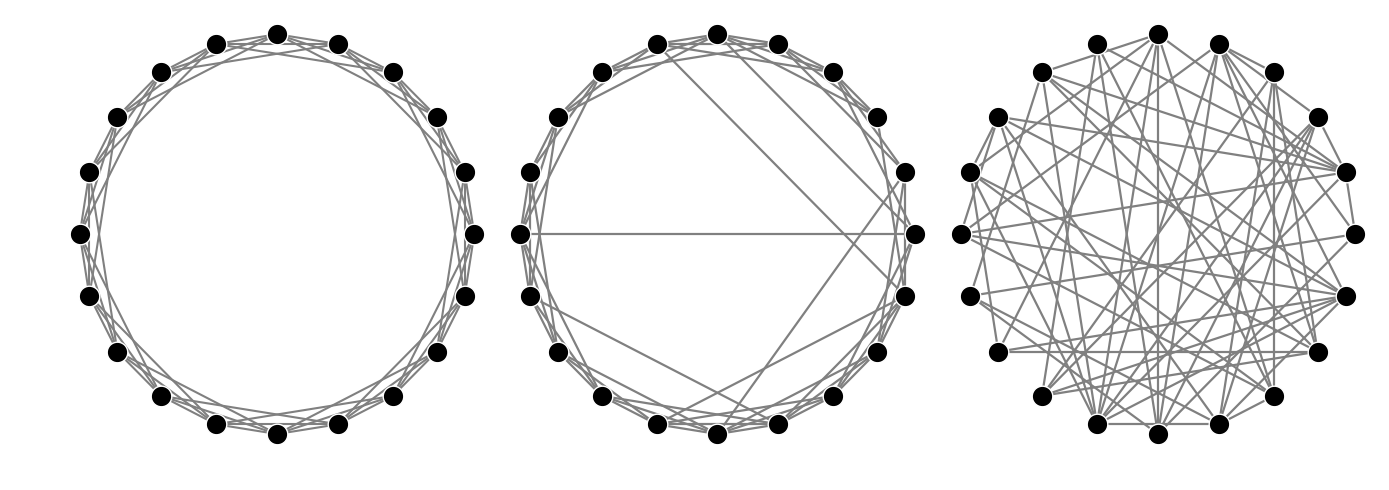

Supplement: Supplementary file 4 — Source data [file 41467_2020_16619_MOESM4_ESM.zip › Structural-core-master/code/Supplementary information code/Expected output/Supplementary note 11/Supplementary Fig. 26 Small-world networks generated by adoption of the Watts-Strogatz...(a).png]

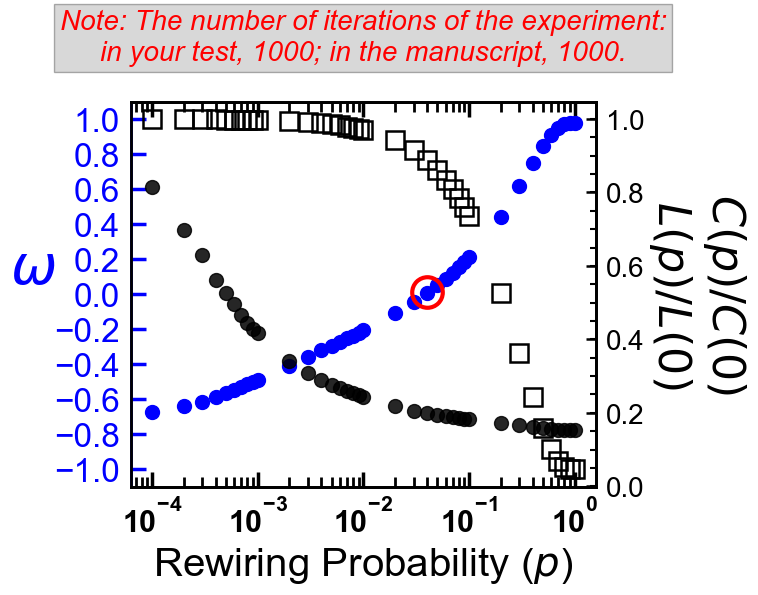

Supplement: Supplementary file 4 — Source data [file 41467_2020_16619_MOESM4_ESM.zip › Structural-core-master/code/Supplementary information code/Expected output/Supplementary note 11/Supplementary Fig. 26 Small-world networks generated by adoption of the Watts-Strogatz...(b).png]

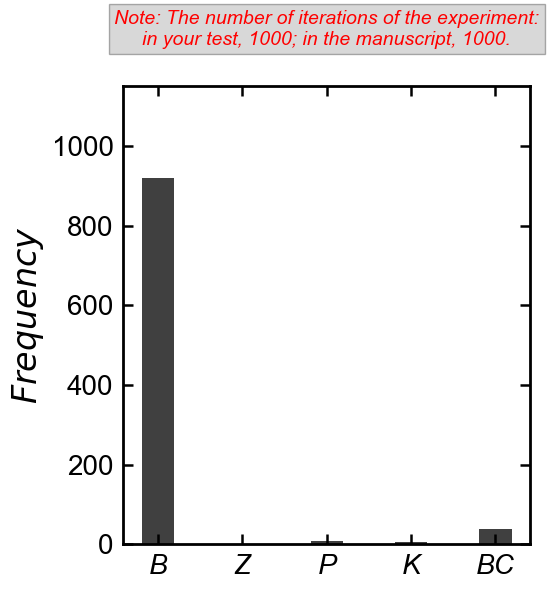

Supplement: Supplementary file 4 — Source data [file 41467_2020_16619_MOESM4_ESM.zip › Structural-core-master/code/Supplementary information code/Expected output/Supplementary note 5/Supplementary Fig. 8 Frequency with which a structural core was detected...over 1000 realisations.png]

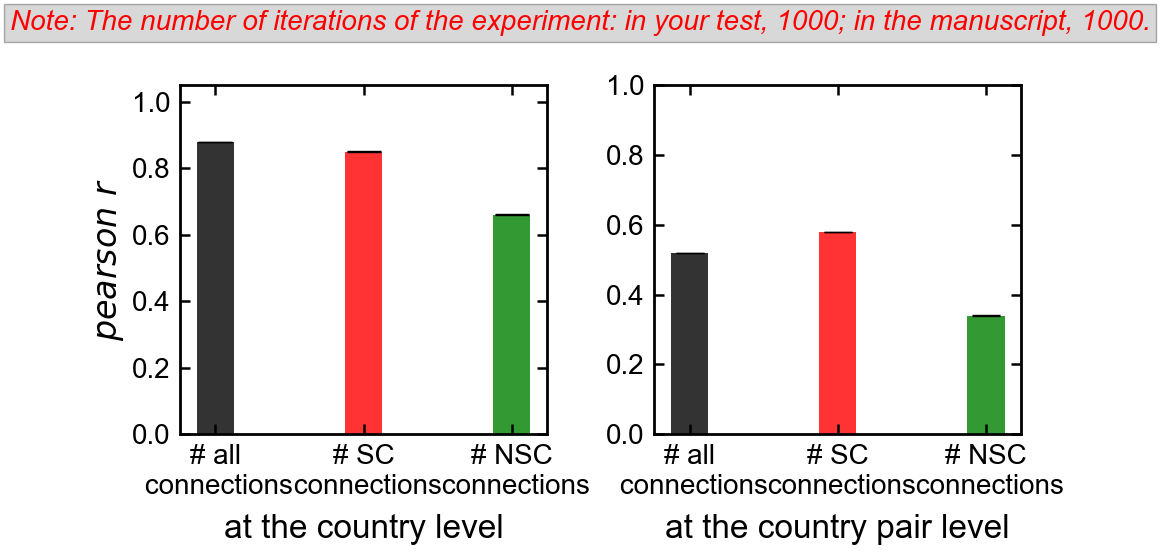

Supplement: Supplementary file 4 — Source data [file 41467_2020_16619_MOESM4_ESM.zip › Structural-core-master/code/Supplementary information code/Expected output/Supplementary note 5/Supplementary Fig. 9 Pearson correlation coefficients...averaged over 1000 repetitions of the experiment.png]

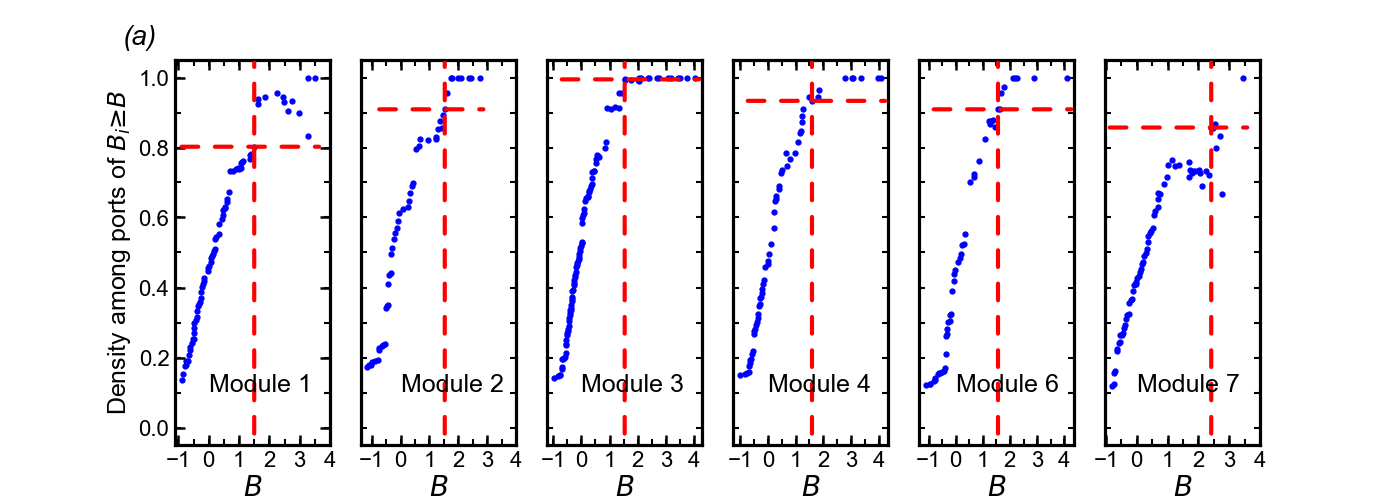

Supplement: Supplementary file 4 — Source data [file 41467_2020_16619_MOESM4_ESM.zip › Structural-core-master/code/Supplementary information code/Expected output/Supplementary note 6/Supplementary Fig. 11 Results for the structural-core...submodular gateway hubs (a).png]

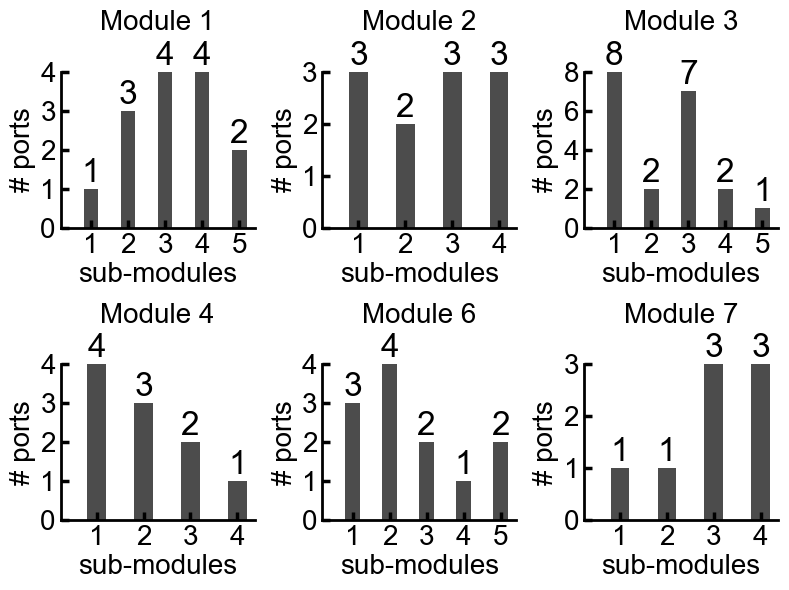

Supplement: Supplementary file 4 — Source data [file 41467_2020_16619_MOESM4_ESM.zip › Structural-core-master/code/Supplementary information code/Expected output/Supplementary note 6/Supplementary Fig. 11 Results for the structural-core...submodular gateway hubs (b).png]

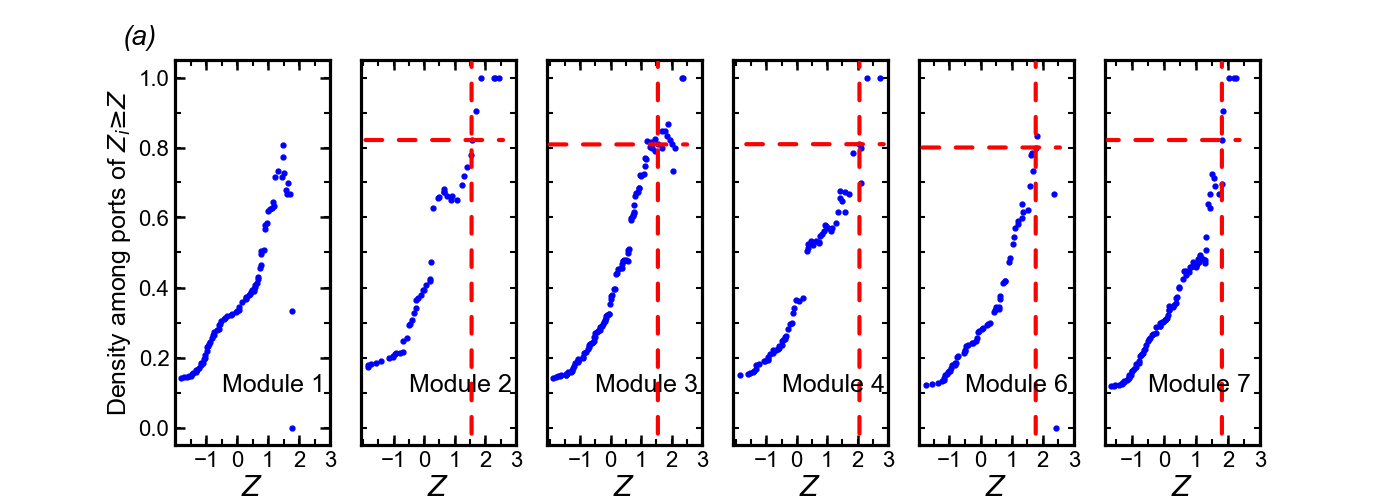

Supplement: Supplementary file 4 — Source data [file 41467_2020_16619_MOESM4_ESM.zip › Structural-core-master/code/Supplementary information code/Expected output/Supplementary note 6/Supplementary Fig. 12 Results for the structural-core...submodular provincial hubs (a).png]

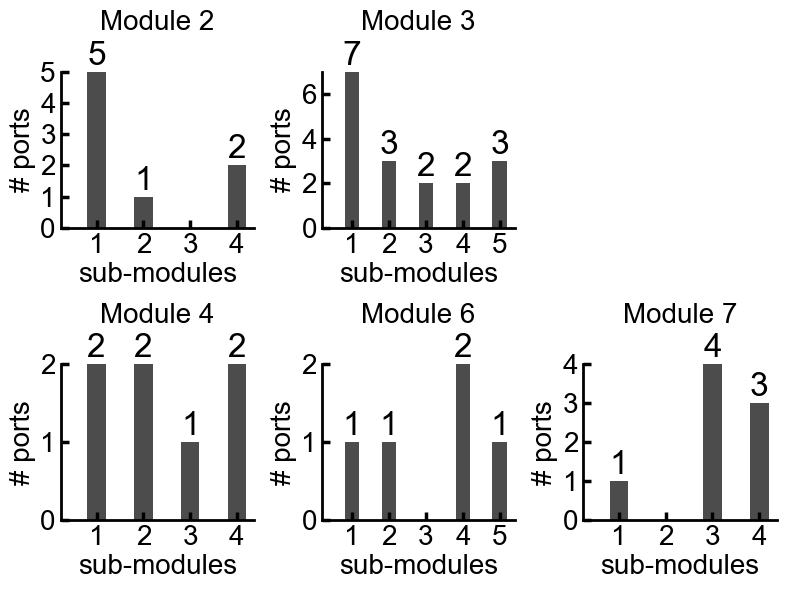

Supplement: Supplementary file 4 — Source data [file 41467_2020_16619_MOESM4_ESM.zip › Structural-core-master/code/Supplementary information code/Expected output/Supplementary note 6/Supplementary Fig. 12 Results for the structural-core...submodular provincial hubs (b).png]

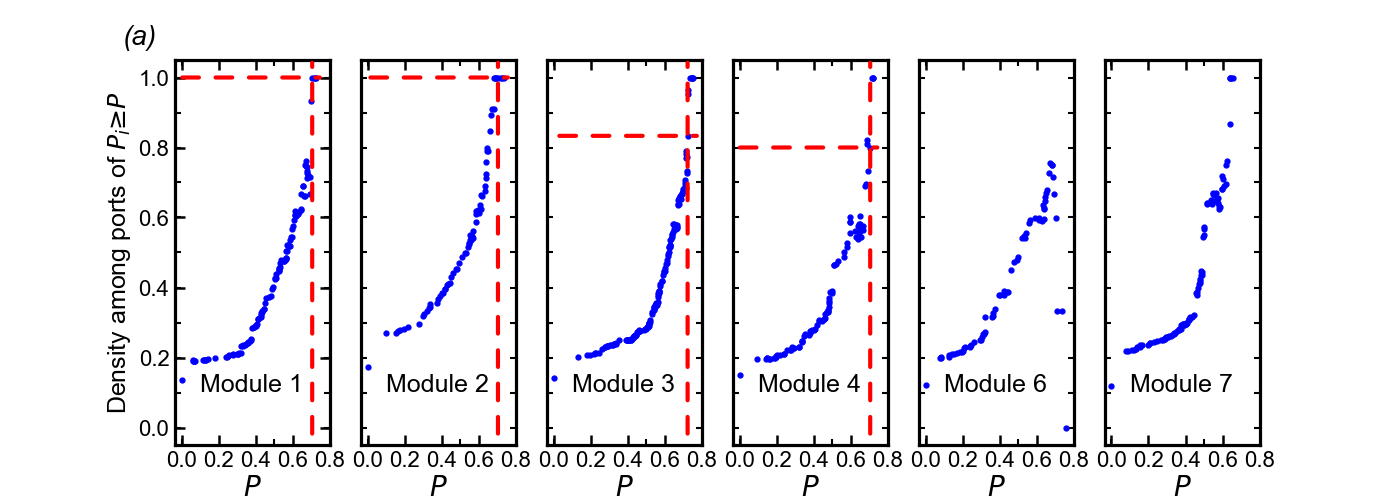

Supplement: Supplementary file 4 — Source data [file 41467_2020_16619_MOESM4_ESM.zip › Structural-core-master/code/Supplementary information code/Expected output/Supplementary note 6/Supplementary Fig. 13 Results for the structural-core...submodular connector hubs (a).png]

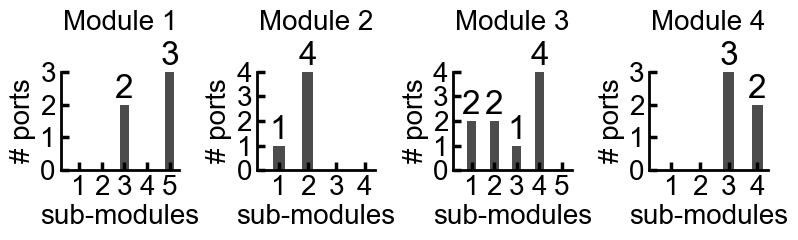

Supplement: Supplementary file 4 — Source data [file 41467_2020_16619_MOESM4_ESM.zip › Structural-core-master/code/Supplementary information code/Expected output/Supplementary note 6/Supplementary Fig. 13 Results for the structural-core...submodular connector hubs (b).png]

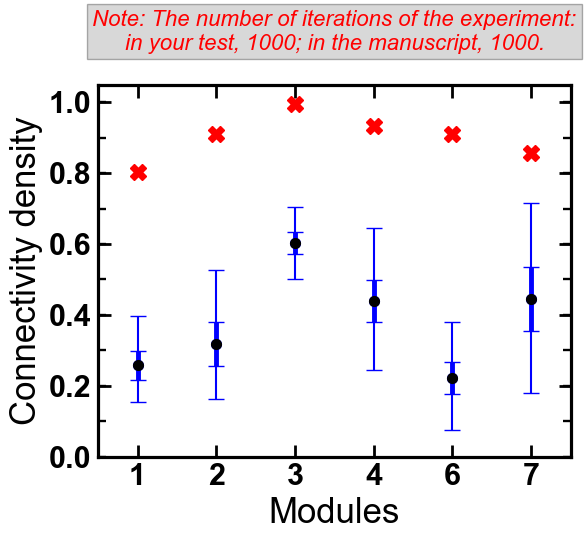

Supplement: Supplementary file 4 — Source data [file 41467_2020_16619_MOESM4_ESM.zip › Structural-core-master/code/Supplementary information code/Expected output/Supplementary note 6/Supplementary Fig. 14 Comparison with permutated networks for the connectivity density...modules.png]

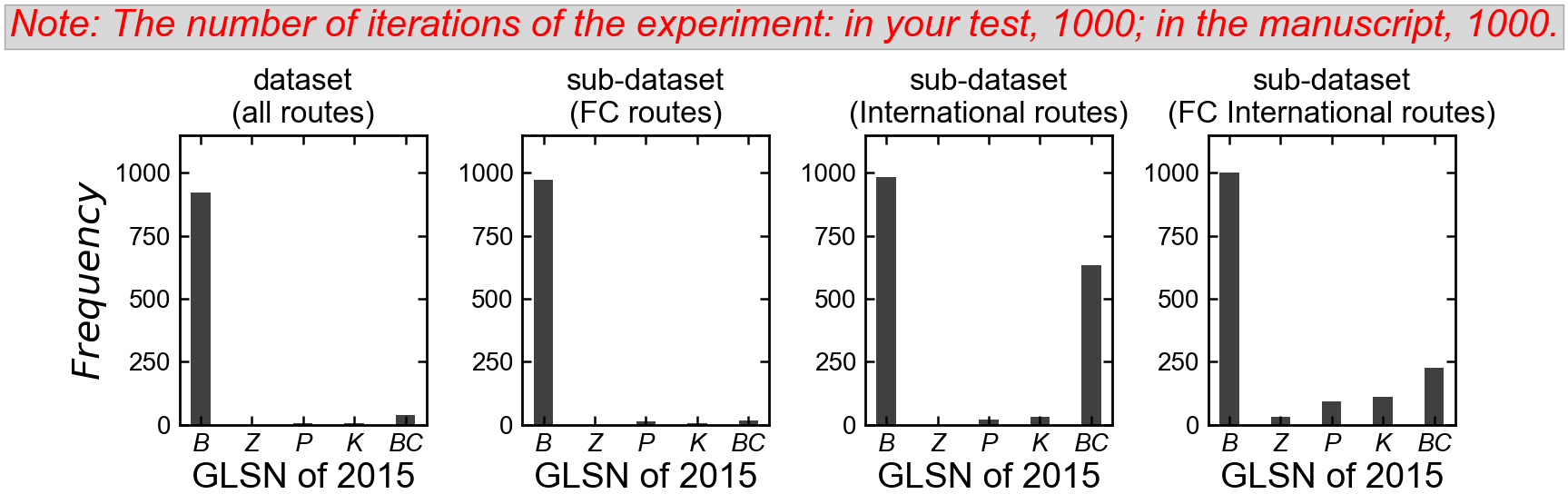

Supplement: Supplementary file 4 — Source data [file 41467_2020_16619_MOESM4_ESM.zip › Structural-core-master/code/Supplementary information code/Expected output/Supplementary note 9/Supplementary Fig. 17 Frequency...across datasets-(a) GLSN of 2015.png]

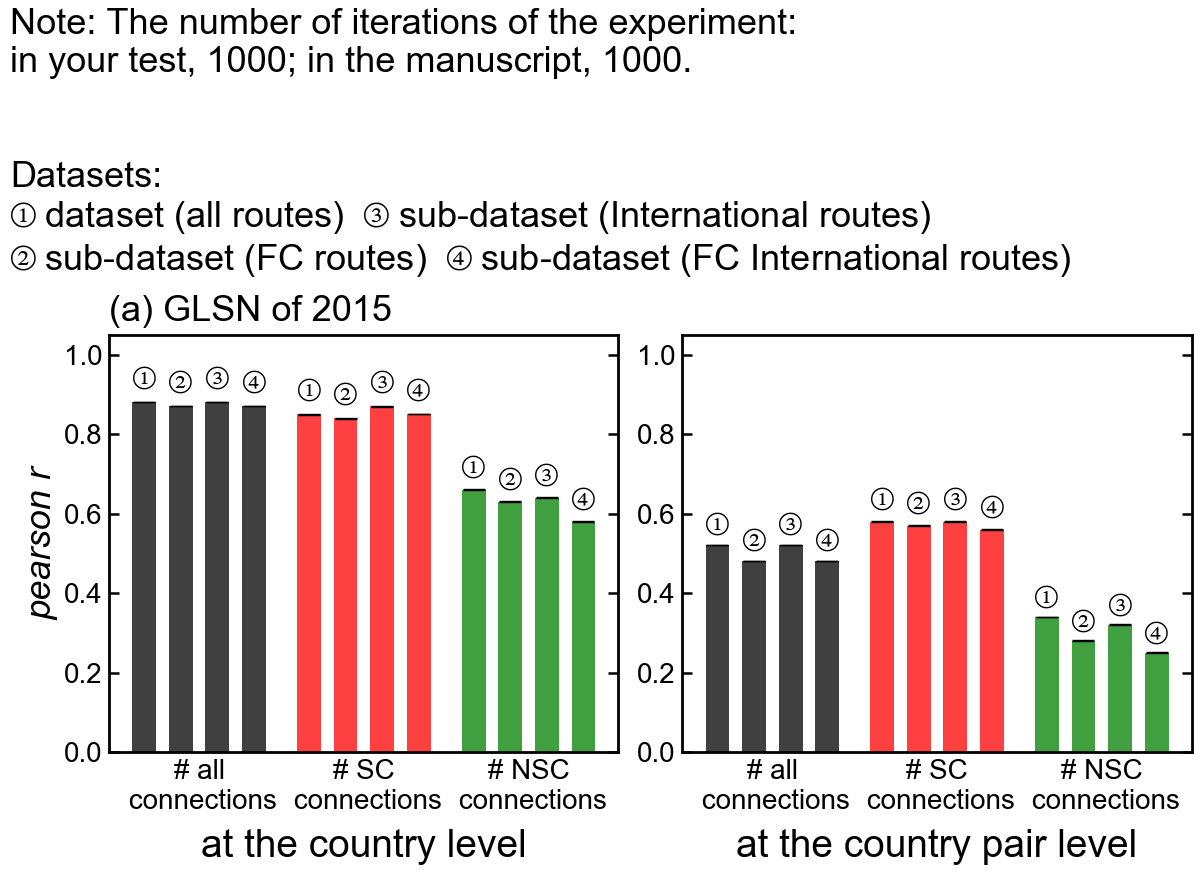

Supplement: Supplementary file 4 — Source data [file 41467_2020_16619_MOESM4_ESM.zip › Structural-core-master/code/Supplementary information code/Expected output/Supplementary note 9/Supplementary Fig. 18 Pearson correlation coefficients...across datasets-(a) GLSN of 2015.png]
